# Integrating Metabolic Modulation and Nanomedicine for Cancer Immunotherapy

**DOI:** 10.1002/advs.202510004

**Published:** 2025-09-12

**Authors:** Xiaosu Zhou, Ruibing Deng, Zunde Liao, Xiaoyu Huang, Junting Huang, Hanlou Yang, Kam W. Leong, Yiling Zhong

**Affiliations:** ^1^ College of Pharmacy, State Key Laboratory of Bioactive Molecules and Druggability Assessment Jinan University Guangzhou Guangdong 511443 China; ^2^ Department of Biomedical Engineering Columbia University New York NY 10027 United States

**Keywords:** immunometabolism, immunotherapy, metabolic reprogramming, nanomedicine, tumor microenvironment

## Abstract

Cancer cells undergo significant metabolic reprogramming to support rapid growth, survival under stress, and resistance to therapies. As our understanding of tumor metabolism and the tumor microenvironment (TME) deepens, there is growing interest in exploiting metabolic vulnerabilities as therapeutic strategies. This review explores key alterations in metabolic pathways, including glucose, amino acid, lipid, nucleotide metabolism, and mitochondrial function, and highlights their impact on tumor progression, the TME, and immune cell function. In addition, the review discusses emerging strategies aimed at targeting these metabolic pathways with a focus on nanomaterial‐based therapies. This includes the use of nanoparticles and drug delivery systems designed to modulate immunometabolism within cancer. These innovative approaches aim to reprogram the TME, enhance immune responses, and improve the targeted delivery of therapeutic agents to tumor sites, offering new ways to overcome conventional therapeutic resistance. Finally, the review also addresses the foreseeable challenges and potential future developments in this field, outlining the opportunities and obstacles that must be addressed for the clinical translation of these strategies in cancer therapy.

## Graphical abstract

Metabolic rewiring in cancer is mapped across glucose, amino acid, lipid, nucleotide, and mitochondrial pathways, with emphasis on immune regulation. The review integrates mechanisms linking these pathways to tumor progression and therapy resistance, and profiles nanomaterial platforms that both deliver therapeutics and actively reprogram immunometabolism. Opportunities, constraints, and future directions are distilled, outlining strategies to overcome resistance and enhance immunotherapy.

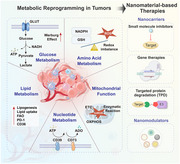

## 1 Introduction

Over the past few decades, the advent of immunotherapies has transformed cancer treatment by expanding the therapeutic options available for managing malignancies.^[^ ^1^, ^2^, ^3^, ^4^, ^5^ ^]^ Immunotherapies, such as immune checkpoint inhibitors (ICIs), adoptive cell therapies, and cancer vaccines, have emerged as promising therapeutic strategies, offering the potential to boost the body's immune response to tumors.^[^ ^2^ ^]^ By enhancing the immune system's ability to recognize and eliminate cancer cells, these therapies have led to remarkable improvements in the survival rates of patients with cancers that were previously considered intractable.^[^ ^6^ ^]^ Despite these successes, many patients either fail to respond initially or develop resistance over time, limiting long‐term benefits.^[^ ^7^ ^]^ These clinical challenges have driven research into the mechanisms underlying the failure of immunotherapy, including the complex interplay between cancer metabolism and immune cell function.^[^ ^8^, ^9^ ^]^ As understanding of this relationship deepens, the field of immunometabolism has emerged, focusing on how metabolic alterations within immune cells influence their functionality and how these metabolic changes shape immune responses, particularly in the context of cancer.^[^ ^7^, ^10^, ^11^ ^]^

The concept of immunometabolism arises from the understanding that immune cells, much like tumor cells, undergo metabolic reprogramming to meet the energetic and biosynthetic demands associated with their activation, differentiation, and function.^[^ ^10^, ^12^, ^13^ ^]^ Immune cells, including T cells, macrophages, and dendritic cells, rely on specific metabolic pathways to respond effectively to inflammatory signals, pathogen invasion, and tumor progression.^[^ ^10^, ^14^ ^]^ These pathways include glucose metabolism, fatty acid oxidation (FAO), amino acid metabolism, and mitochondrial function. In the context of cancer, immune cells are exposed to the altered metabolic environment of the tumor microenvironment (TME), which profoundly impacts their ability to mount effective immune responses.^[^ ^15^, ^16^, ^17^ ^]^ The TME, marked by hypoxia, nutrient deprivation, and acidosis, creates conditions that impair immune cell function and promote immune evasion.^[^ ^18^, ^19^, ^20^ ^]^ Understanding the intersection of tumor metabolism and immune cell metabolism, or “immunometabolism”, is essential for developing strategies to overcome these challenges and improve the effectiveness of cancer immunotherapies.^[^ ^17^, ^21^ ^]^

Cancer metabolism plays a pivotal role not only in sustaining tumorigenesis and promoting survival but also in modulating the immune landscape.^[^ ^17^, ^22^ ^]^ Tumor cells often exhibit altered metabolic pathways that support rapid cell growth and resistance to therapy.^[^ ^9^, ^21^ ^]^ These include increased aerobic glycolysis (the Warburg effect), enhanced fatty acid synthesis and oxidation, and upregulated nucleotide biosynthesis. These metabolic changes enable tumor cells to meet the demands of rapid proliferation and to adapt to the hostile conditions within the TME. However, tumor cells also secrete a variety of metabolites that influence immune cell behavior.^[^ ^23^, ^24^, ^25^ ^]^ For example, lactate, prostaglandin E_2_ (PGE_2_), and arginine are key metabolites that can modulate immune cell function. Lactate, produced during glycolysis, has been shown to inhibit the function of cytotoxic T cells and natural killer (NK) cells to promote immune suppression.^[^ ^26^ ^]^ Similarly, PGE_2_, a product of the cyclooxygenase (COX) pathway, can promote the differentiation of regulatory T cells (Tregs) and suppress effector T cell activity and contribute to immune evasion.^[^ ^27^, ^28^ ^]^ Arginine, which is essential for T cell activation, can be depleted in the TME through the action of ectonucleotidases, such as CD73, leading to immune suppression.^[^ ^29^ ^]^

In addition to the direct metabolic effects on immune cells, the energetic interplay between tumor cells and immune cells creates metabolic competition within the TME.^[^ ^9^ ^]^ Tumor cells consume large amounts of glucose and other nutrients to limit the availability of these resources for immune cells. This nutrient competition is exacerbated by the acidic environment of the TME, which further impairs immune cell function. The presence of high lactate levels and low pH not only suppresses immune effector functions but also promotes the polarization of tumor‐associated macrophages (TAMs) toward an immunosuppressive M2 phenotype.^[^ ^30^ ^]^ This metabolic competition between tumor cells and immune cells is a key mechanism by which tumors evade immune surveillance and promote tumor progression.

Given the critical role of metabolism in immune modulation, targeting cancer cell metabolism presents a novel therapeutic approach to enhance antitumor immunity.^[^ ^22^ ^]^ Recent studies have demonstrated that altering tumor metabolism through small molecules or metabolic inhibitors can significantly impact the immune response within the TME.^[^ ^31^, ^32^, ^33^ ^]^ For example, targeting key metabolic pathways such as glycolysis, glutamine metabolism, and FAO has shown promise in preclinical models. However, there are significant challenges associated with targeting cancer metabolism for immune modulation. One of the major hurdles is the complexity of metabolic pathways in both tumor cells and immune cells. Many metabolic pathways are shared between tumor cells and immune effector cells, such as CD8^+^ T cells, which rely on similar metabolic processes, including glycolysis and oxidative phosphorylation.^[^ ^34^ ^]^ This overlap means that targeting tumor metabolism can inadvertently affect immune cell function, potentially impairing the antitumor immune response. Specifically, CD8^+^ T cells require metabolic reprogramming to differentiate into effector cells capable of killing tumor cells.^[^ ^35^, ^36^ ^]^ Inhibiting key metabolic pathways in tumor cells may also simultaneously limit the metabolic resources available to immune cells, thereby reducing their efficacy.

Another challenge is the development of metabolic modulators that selectively target cancer cells only. Many of the metabolic inhibitors currently under investigation have limited specificity for cancer cells, and their effects on immune cells can be unpredictable.^[^ ^32^ ^]^ Additionally, the TME is highly heterogeneous,^[^ ^37^ ^]^ with varying degrees of metabolic reprogramming across different regions of the tumor. This heterogeneity complicates the development of effective therapies, as the metabolic vulnerabilities of tumors may not be uniform throughout the TME.^[^ ^38^, ^39^ ^]^ Despite these challenges, there is increasing interest in integrating metabolic modulation with immunotherapy to overcome resistance mechanisms and improve treatment outcomes.^[^ ^40^, ^41^ ^]^ For instance, recent studies have evaluated the combination of ICIs with metabolic inhibitors to reprogram immune cell metabolism and strengthen antitumor immune responses.^[^ ^42^, ^43^, ^44^ ^]^ Such strategies can enhance the activity of effector immune cells or mitigate immunosuppressive mechanisms within the TME, offering a promising avenue to increase the clinical efficacy of immunotherapy.

Nanotechnology‐based therapies address key challenges in cancer treatment, including drug resistance, limited bioavailability, and off‐target toxicity.^[^ ^45^, ^46^, ^47^ ^]^ Nanoparticle delivery systems enhance the specificity, solubility, and stability of metabolic inhibitors, improving therapeutic efficacy while minimizing systemic side effects. By enabling targeted delivery to tumor cells, nanoparticles reduce systemic exposure and improve treatment outcomes. They also allow for the co‐delivery of agents, such as metabolic inhibitors and immune modulators, offering a strategy to amplify efficacy and circumvent resistance mechanisms.^[^ ^48^, ^49^ ^]^ Moreover, certain nanoparticles exhibit intrinsic immunometabolic regulatory properties for enhanced immune responses.^[^ ^50^, ^51^, ^52^ ^]^ Advances in nanoparticle design have further optimized the delivery of metabolic therapies, offering a promising path to more precise, effective cancer treatments and improved responses to immunotherapy.^[^ ^53^, ^54^ ^]^

**Figure 1:**
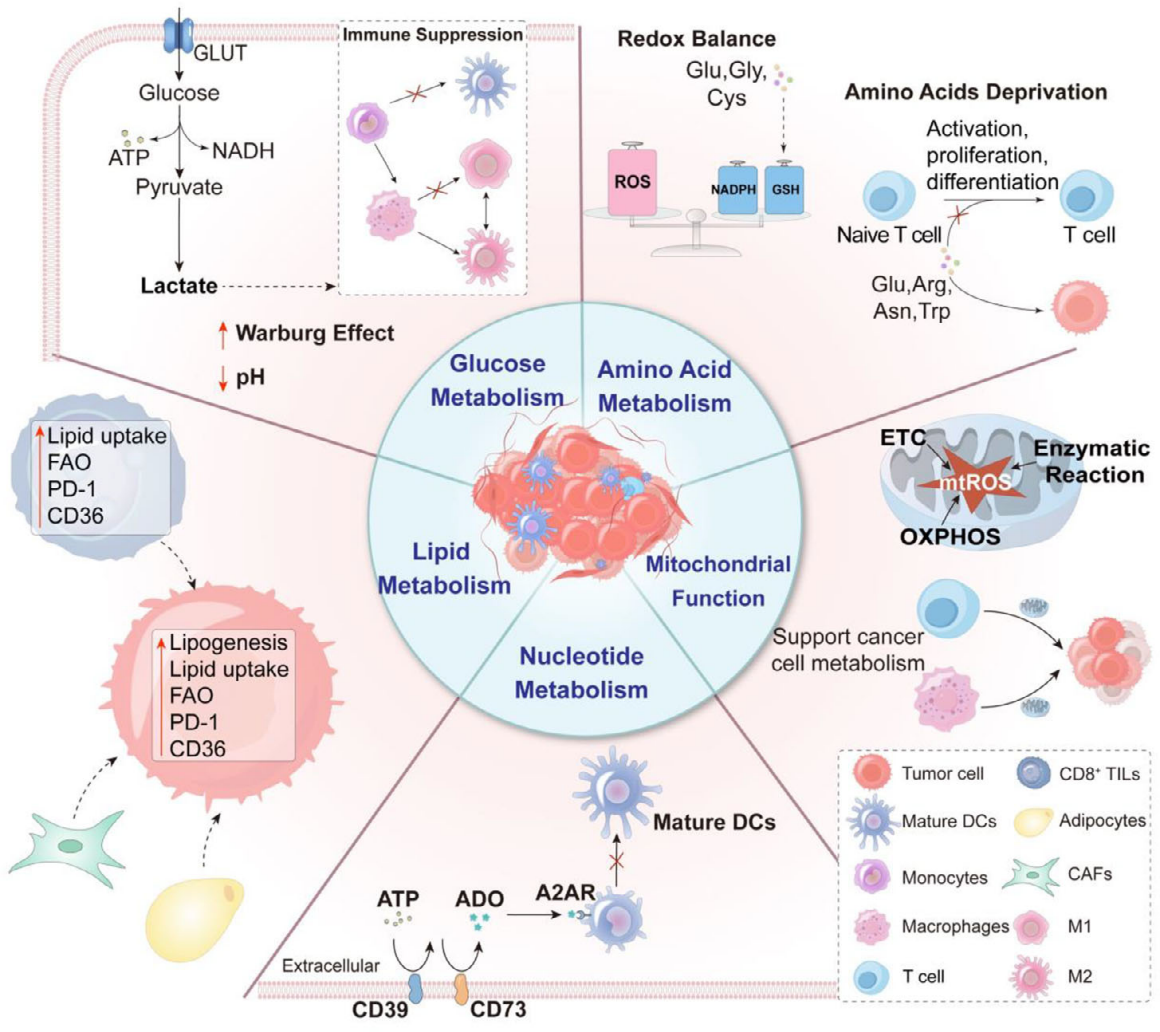
Schematic representation of tumor metabolic reprogramming. This diagram illustrates key alterations in glucose, amino acid, lipid, and nucleotide metabolism, as well as mitochondrial function, that together drive tumor growth, survival, and therapeutic resistance.

This review examines the major metabolic pathways altered in cancer—covering glucose, amino acid, lipid, nucleotide, and mitochondrial metabolism—and their influence on the TME and immune function (**Figure** **1**). Distinct from previous reviews,^[^ ^55^, ^56^, ^57^, ^58^, ^59^, ^60^ ^]^ we provide a comprehensive and integrative analysis of these pathways and highlight their interconnected roles in tumor progression, immune modulation, and therapy resistance. In particular, we emphasize recent advances in nanomaterial‐based strategies, which function both as delivery platforms for small‐molecule inhibitors, gene therapies, and targeted protein degraders, and as active metabolic modulators (“nanomodulators”) capable of reprogramming the TME to enhance antitumor immunity. Finally, we examine the challenges and future directions for the clinical translation of these metabolic‐targeted therapies, offering insights into how these strategies could transform cancer treatment in the near future.

## 2 Metabolic Reprogramming in Tumors

Metabolic reprogramming enables cancer cells to adapt to the hypoxic and nutrient‐deprived TME, supporting their proliferation, survival, and metastasis.^[^ ^10^, ^24^, ^61^ ^]^ Key metabolic pathways—glycolysis, oxidative phosphorylation, amino acid metabolism, and lipid biosynthesis—are commonly altered, with effects extending beyond tumor cells to stromal and immune cells. Dysregulated metabolism not only drives tumor growth but also promotes immune evasion, therapeutic resistance, and disease progression.^[^ ^9^, ^62^ ^]^ A deeper understanding of these alterations is essential for developing therapies that target cancer metabolism and modulate the immune response. This section explores major metabolic pathways implicated in tumor biology and therapeutic strategies.

### 2.1 Glucose Metabolism Alteration

One of the most prominent metabolic alterations in cancer cells is the reprogramming of glucose metabolism, often referred to as the Warburg effect.^[^ ^63^ ^]^ Despite the presence of oxygen, many cancer cells rely predominantly on glycolysis for energy production, a phenomenon that supports rapid cell proliferation and survival in the hypoxic TME. This shift in glucose metabolism is accompanied by an increase in glucose uptake and the conversion of glucose to lactate, even in the presence of oxidative phosphorylation. The altered glucose metabolism not only provides energy for tumor cells but also generates intermediates that contribute to anabolic processes, such as nucleic acid and lipid synthesis.^[^ ^21^, ^64^ ^]^ Additionally, this metabolic shift can influence immune cell function, contributing to immune suppression within the TME.^[^ ^23^ ^]^

#### 2.1.1 Aerobic Glycolysis

Unlike normal cells, cancer cells often upregulate the expression of glucose transporter proteins (GLUTs), enhancing glucose uptake and supporting growth through the energy‐inefficient process of aerobic glycolysis.^[^ ^65^, ^66^, ^67^ ^]^ It occurs in two main stages. First, glucose is converted into pyruvate, generating nicotinamide adenine dinucleotide (NADH) and a small amount of adenosine triphosphate (ATP). Under normal conditions, NADH is typically reoxidized in the mitochondrial electron transport chain, leading to further ATP production via oxidative phosphorylation. However, in cancer cells undergoing aerobic glycolysis, pyruvate is predominantly converted to lactate instead of being fully oxidized, despite the presence of sufficient oxygen.^[^ ^57^ ^]^ This shift results from the increased demand for macromolecule biosynthesis, such as lipids and proteins, rather than focusing solely on energy production. The intermediate products of glycolysis are shunted into several key biosynthetic pathways, including serine metabolism, the hexosamine biosynthetic pathway, and the pentose phosphate pathway.^[^ ^68^ ^]^ These metabolic pathways, often dysregulated in cancer, provide essential metabolites such as reduced nicotinamide adenine dinucleotide phosphate (NADPH), which supports reductive biosynthesis and helps mitigate oxidative stress, as well as S‐adenosyl methionine (SAM), which is critical for methylation reactions and the synthesis of nucleic acids and proteins.^[^ ^69^ ^]^

The shift toward aerobic glycolysis in tumor cells has profound effects on immune cell function.^[^ ^70^ ^]^ Activated CD8⁺ and CD4⁺ T cells rely heavily on glycolysis to fuel mechanistic target of rapamycin complex 1 (mTORC1) signaling, drive Myc‐ and hypoxia‐inducible factor‐1α (HIF‐1α)‐mediated transcriptional programs, and support the biosynthesis necessary for clonal expansion and cytokine production. However, tumor cells often overexpress glucose transporter 1 (GLUT1) and hexokinase 2 (HK2), leading to excessive glucose uptake and depletion of extracellular glucose, which limits mTORC1 activation in effector T cells and impairs their proliferation and interferon‐γ (IFN‐γ) production. Concurrently, elevated levels of tumor‐derived lactate, exported via monocarboxylate transporters (MCTs) such as MCT4, contribute to acidification of the TME. Acidic pH inhibits T cell receptor (TCR) signaling, impairs nuclear translocation of nuclear factor of activated T cells (NFAT), and suppresses perforin and granzyme expression, thereby reducing T cell cytotoxic function.^[^ ^17^, ^71^, ^72^ ^]^ Beyond T cells, metabolic reprogramming in the TME promotes the expansion and suppressive activity of myeloid‐derived suppressor cells (MDSCs) and Tregs.^[^ ^73^, ^74^ ^]^ MDSCs utilize both glycolysis and FAO to sustain the expression of arginase‐1 and inducible nitric oxide synthase (iNOS), which deplete key nutrients such as L‐arginine and inhibit T cell activity.^[^ ^9^, ^17^ ^]^ Similarly, tumor‐derived lactate and adenosine enhance the stability and suppressive capacity of FoxP3⁺ Tregs by promoting epigenetic remodeling and increasing reliance on oxidative phosphorylation.^[^ ^75^, ^76^, ^77^ ^]^ Collectively, these mechanisms demonstrate how tumor‐driven metabolic reprogramming reshapes immune cell metabolism and signaling, leading to impaired antitumor immunity and enhanced immune evasion.

#### 2.1.2 Lactate Accumulation

The accumulation of lactate resulting from the high glycolytic activity of tumor cells is a prominent feature of the TME. The increased lactate concentration has several profound effects on tumor progression, immune suppression, and resistance to treatment.

##### Acidification of the TME

As lactate accumulates in the TME, it dissociates into lactate ions and protons (H^+^), leading to a reduction in pH and the acidification of the microenvironment.^[^ ^78^ ^]^ This acidic condition creates a more favorable environment for tumor cell survival and growth while simultaneously impairing the function of normal, non‐cancerous cells, including immune cells.^[^ ^79^ ^]^ Tumor cells, particularly those undergoing glycolysis, have adapted to function in a low pH environment, whereas immune cells, especially cytotoxic T lymphocytes (CTLs), are highly sensitive to such acidic conditions. The low pH inhibits the activation and proliferation of immune cells, thereby impeding effective immune responses against tumors.^[^ ^26^, ^80^ ^]^

##### Immune Suppression and Impairment of T Cell Function

Lactate directly inhibits T cell function, contributing to immune evasion in TME.^[^ ^81^ ^]^ The acidity in the TME induces an anergic state in tumor‐infiltrating lymphocytes (TILs), reducing their activation and functions. Intracellular acidification has been shown to decrease the expression of NFAT and impair T cell survival.^[^ ^26^ ^]^ The acidic TME diminishes the ability of CD8^+^ cytotoxic T cells to perform key effector functions, such as cytokine production and cytotoxicity. In contrast, Tregs, which play a crucial role in maintaining peripheral tolerance, exhibit a metabolic advantage over effector T cells in the acidic TME. This advantage allows Tregs to thrive in the hostile environment, further promoting immune suppression and hindering effective antitumor immunity.^[^ ^82^ ^]^ Additionally, lactate inhibits TCR signaling, leading to reduced immune activation. Lactate also promotes the expression of immune checkpoint molecules, such as PD‐L1, on tumor cells and immune cells, further suppressing T cell activity.^[^ ^83^ ^]^ This contributes to immune escape and allows tumors to evade immune surveillance despite the presence of active immune cells. Beyond its effects on T cells, lactate acts as a key metabolic and signaling mediator in the TME. It promotes the polarization of TAMs toward an M2‐like, immunosuppressive phenotype, partly through HIF‐1α stabilization and activation of G protein‐coupled receptor 132 (GPR132). This reprogramming leads to upregulation of arginase‐1 and vascular endothelial growth factors (VEGFs), while concurrently suppressing the production of pro‐inflammatory cytokines such as interleukin‐6 (IL‐6) and tumor necrosis factor‐alpha (TNF‐α).^[^ ^76^, ^84^, ^85^, ^86^ ^]^ Together, these mechanisms reinforce immune tolerance and promote tumor progression.

##### Promotion of Tumor Cell Invasion and Metastasis

The acidic TME, driven by lactate accumulation, enhances tumor invasiveness.^[^ ^87^, ^88^ ^]^ The low pH activates genes involved in epithelial‐to‐mesenchymal transition (EMT), a process that enables tumor cells to lose their epithelial characteristics and acquire migratory and invasive properties.^[^ ^89^ ^]^ Lactate also stimulates the secretion of matrix metalloproteinases (MMPs), enzymes that degrade the extracellular matrix and facilitate tumor cell invasion into surrounding tissues.^[^ ^88^ ^]^ This process contributes to metastasis, allowing cancer cells to disseminate to distant organs.

##### Regulation of Cancer‐Associated Fibroblasts (CAFs) and MDSCs

Lactate accumulation in the TME modulates stromal cells, activating CAFs to secrete growth factors and extracellular matrix components that promote tumor growth and metastasis.^[^ ^90^ ^]^ It also enhances the accumulation and immunosuppressive activity of MDSCs,^[^ ^84^ ^]^ further suppressing antitumor immune responses.

##### Modulation of Tumor Angiogenesis

Lactate stimulates angiogenesis by upregulating the expression of hypoxia‐inducible factors (HIFs) and VEGFs.^[^ ^77^, ^91^ ^]^ In the TME, lactate‐induced angiogenesis provides tumors with essential nutrients and oxygen, thereby sustaining their rapid growth. This not only supports tumor progression but also contributes to the dynamic changes in the TME that favor cancer cell survival.

##### Resistance to Therapy

The acidified TME and lactate accumulation can contribute to resistance to standard cancer treatments such as chemotherapy and radiation. The low pH can reduce the efficacy of chemotherapy by promoting the survival of cancer cells that are less sensitive to these therapies. Additionally, lactate‐induced immune suppression can compromise the effectiveness of immunotherapy, as the reduced function of immune cells in the TME hinders the ability of ICIs and other immunotherapeutic strategies to effectively target tumors.

##### Therapeutic Implications

Given the significant role of lactate in promoting tumor progression and immune evasion, targeting lactate metabolism or lactate‐mediated signaling pathways offers promising therapeutic potential.^[^ ^92^, ^93^ ^]^ Strategies to inhibit lactate production, such as targeting lactate dehydrogenase (LDH) or lactate transporter (e.g., MCT1), could reduce tumor growth and increase the sensitivity of cancer cells to chemotherapy and immunotherapy. Neutralization of tumor acidity in vivo has been shown to enhance the efficacy of immune checkpoint blockade (ICB), emphasizing the role of the TME acidity in modulating TIL function.^[^ ^71^ ^]^ Additionally, buffering the acidic TME or modulating lactate's effects on immune cells could restore immune cell function and enhance the effectiveness of cancer treatments.^[^ ^94^ ^]^

### 2.2 Amino Acid Metabolism Alteration

Beyond the rewiring of glucose metabolism, cancer cells extensively reprogram amino acid metabolism to support growth, survival, and immune evasion.^[^ ^95^, ^96^ ^]^ These metabolic shifts supply essential substrates for protein, nucleotide, and lipid synthesis, while also regulating redox homeostasis, epigenetic modifications, and signaling networks.^[^ ^58^, ^97^ ^]^ Key amino acids—including glutamine, arginine, glycine, aspartate, serine, and leucine—are frequently rerouted to support the enhanced anabolic and survival requirements of malignant cells. Furthermore, altered amino acid metabolism within the TME impairs immune cell function, exacerbating immune suppression and tumor progression.^[^ ^95^ ^]^ This section explores the mechanisms behind amino acid metabolism alterations in cancers, their role in tumor biology, and their potential as therapeutic targets to improve cancer treatment outcomes.

#### 2.2.1 Amino Acid Metabolism

Amino acids are vital for tumor growth and for the remodeling of stromal and vascular components in the inflamed TME during tumor progression.^[^ ^98^ ^]^ Additionally, amino acids act as key fuels supporting cancer cell development. For example, glutamine, a critical amino acid in many tumors, is primarily anaplerotic, providing nitrogen and carbon to replenish intermediates in the tricarboxylic acid (TCA) cycle,^[^ ^99^ ^]^ thereby supporting cellular metabolism and energy production. Besides glutamine, other amino acids, such as alanine and aspartate, can serve as alternative fuel sources, supporting cellular processes in tumor cells.^[^ ^100^ ^]^

Amino acids also contribute to various biosynthetic pathways critical for cancer cell proliferation and survival. For instance, branched‐chain amino acids are metabolized to acetyl‐CoA, a key precursor in lipogenesis, which fuels the synthesis of cellular membranes required for the rapid growth of cancer cells.^[^ ^101^ ^]^ Additionally, amino acids play a significant role in nucleotide biosynthesis, which is crucial for cell division.^[^ ^102^ ^]^ Glycine, glutamine, and aspartate provide carbon and nitrogen for purine synthesis,^[^ ^103^ ^]^ while glycine, serine, and methionine contribute one‐carbon units for nucleobase formation through the methionine‐folate cycle.^[^ ^69^, ^104^ ^]^ These pathways enable the synthesis of the nucleotides required for DNA and RNA production in proliferating tumor cells.

Cancer cell proliferation leads to the accumulation of reactive oxygen species (ROS), which can damage cellular macromolecules and induce cell death. To counteract oxidative stress, cancer cells rely on the synthesis of glutathione (GSH) from glutamate, glycine, and cysteine, a process that helps maintain cellular redox balance.^[^ ^105^ ^]^ Glutamine plays a vital role in sustaining redox homeostasis through several mechanisms. Metabolites generated during the TCA cycle provide precursors for NADPH, a key reducing agent that supports antioxidant defenses. While NADPH is commonly associated with redox balance, a significant portion is also produced by the folate cycle, driven primarily by one‐carbon units derived from serine.^[^ ^106^ ^]^ Furthermore, the exchange of intracellular glutamate through the transporter SLC7A11 facilitates cystine uptake, which is then reduced to cysteine, the rate‐limiting precursor for GSH biosynthesis.^[^ ^107^ ^]^ Both NADPH and GSH are central regulators of cellular redox status, enabling tumor cells to cope with oxidative stress and maintain their proliferative capabilities.^[^ ^108^ ^]^

#### 2.2.2 Impact of Amino Acid Metabolism Alteration in the TME

The TME imposes significant constraints on amino acid availability, thereby modulating immune cell function through defined metabolic pathways.^[^ ^36^, ^109^, ^110^, ^111^ ^]^ Effector T cells depend on amino acids such as glutamine, serine, and tryptophan to sustain mTORC1 signaling, support nucleotide and lipid biosynthesis, and maintain redox homeostasis—processes essential for clonal expansion and cytokine production.^[^ ^110^, ^111^, ^112^ ^]^ Glutamine acts as a key anaplerotic substrate for the TCA cycle and is required for mTORC1 activation and c‐Myc‐driven transcriptional programs that support T cell growth and effector function. Serine contributes to the one‐carbon metabolism pathway, providing carbon units for purine and thymidine synthesis and supporting GSH generation to buffer ROS. Tryptophan, on the other hand, is catabolized by tumor‐ and myeloid‐expressed indoleamine 2,3‐dioxygenase 1 (IDO1), resulting in local tryptophan depletion and accumulation of kynurenine (Kyn), an immunosuppressive metabolite. Kyn activates the aryl hydrocarbon receptor (AhR) in T cells, leading to T cell anergy, impaired cytotoxicity, and the induction of Treg differentiation.^[^ ^113^ ^]^ Unlike effector T cells, Tregs exhibit greater metabolic adaptability and can maintain their suppressive function in nutrient‐deprived environments by relying more heavily on oxidative phosphorylation and utilizing alternative substrates such as fatty acids or tumor‐derived lactate.^[^ ^114^ ^]^ Moreover, AhR signaling in Tregs enhances FoxP3 stability and reinforces their immunosuppressive phenotype. Collectively, these amino acid‐driven metabolic adaptations reprogram immune cell fate and function within the TME, attenuating antitumor immunity while promoting immune tolerance and tumor progression.^[^ ^17^, ^115^ ^]^

Altered amino acid metabolism supports tumor cell proliferation and survival by providing critical biosynthetic precursors and maintaining redox homeostasis. For example, glutamine replenishes TCA cycle intermediates and donates nitrogen for the synthesis of nucleotides and proteins.^[^ ^99^ ^]^ Rapidly proliferating cancer cells exhibit increased demand for glutamine, serine, and glycine to fuel anabolic processes essential for DNA replication, protein translation, and membrane biosynthesis.^[^ ^116^ ^]^ This metabolic reprogramming enables sustained nucleotide, lipid, and protein biosynthesis, all of which are essential for tumor growth.^[^ ^95^, ^111^, ^115^ ^]^ In addition, branched‐chain amino acids, particularly leucine, activate mTORC1 signaling, which promotes protein synthesis, cell growth, and proliferation.^[^ ^117^ ^]^ As tumors grow, they generate excessive ROS, which can damage cellular structures and threaten viability. To maintain redox balance, tumor cells depend on amino acids such as glutamine, glycine, and cysteine to synthesize GSH, a major intracellular antioxidant. Amino acid metabolism also contributes to NADPH generation, particularly through serine‐driven one‐carbon metabolism, which helps neutralize ROS.^[^ ^108^ ^]^ These adaptations enable tumor cells to survive oxidative stress within the TME. However, this altered redox landscape has immunological consequences. CTLs and NK cells are highly sensitive to oxidative stress and rely on antioxidants like GSH to maintain their effector functions.^[^ ^118^, ^119^ ^]^ Tumor‐driven reprogramming of amino acid metabolism can deplete precursors for antioxidant synthesis, thereby impairing immune cell function and contributing to immune evasion.

Altered amino acid metabolism contributes to tumor cell migration and invasion by modulating signaling pathways and enzymatic processes involved in EMT and extracellular matrix (ECM) remodeling.^[^ ^120^ ^]^ Amino acids such as glutamine, serine, and glycine influence EMT‐related signaling cascades and supply metabolic intermediates required for the synthesis of enzymes that degrade the ECM. For example, glutamine metabolism supports the expression and activity of MMPs, which break down ECM components and facilitate tumor invasion.^[^ ^121^, ^122^ ^]^ In addition, leucine and other branched‐chain amino acids activate mTORC1 signaling,^[^ ^123^ ^]^ which regulates cytoskeletal reorganization, promotes cell motility, and enhances adhesion—key processes that drive metastatic dissemination.

Altered amino acid metabolism also impacts stromal cells in the TME, particularly CAFs.^[^ ^95^ ^]^ Amino acids such as glutamine and serine activate metabolic pathways in CAFs that enhance their pro‐tumorigenic functions, including the secretion of growth factors, cytokines, and ECM components. Specifically, glutamine metabolism in CAFs promotes the production of fibronectin and other ECM proteins that facilitate tumor cell migration and tissue remodeling.^[^ ^112^, ^124^ ^]^

Altered amino acid metabolism also contributes to tumor angiogenesis through the regulation of key signaling and biosynthetic pathways.^[^ ^112^ ^]^ Serine and glutamine serve as primary sources of one‐carbon units that support the folate and methionine cycles, which are essential for nucleotide biosynthesis and methylation‐dependent gene regulation. These metabolic processes promote the expression of VEGFs, which are key regulators of tumor‐associated angiogenesis and vascular remodeling. Glutamine metabolism, in particular, activates the mTORC1 pathway, which promotes HIF‐1α stabilization under hypoxic conditions, leading to upregulation of VEGF transcription. Through these mechanisms, amino acid‐driven signaling supports the formation of new blood vessels, enabling tumor growth and metastatic dissemination.^[^ ^125^ ^]^

Reprogramming of amino acid metabolism is a key contributor to therapeutic resistance in cancer.^[^ ^112^ ^]^ Tumor cells frequently depend on amino acids such as glutamine and serine to counteract the cytotoxic effects of chemotherapy and radiotherapy, which primarily induce ROS accumulation and DNA damage. Glutamine supports GSH biosynthesis and anaplerotic entry into the TCA cycle, while serine contributes to one‐carbon metabolism and NADPH production via the folate cycle, together enhancing antioxidant capacity, promoting DNA repair, and suppressing apoptosis. These metabolic adaptations enable cancer cells to withstand conventional therapies.

In addition, amino acid metabolism activates key stress‐response pathways, including NRF2, which induces the expression of antioxidant genes,^[^ ^126^ ^]^ and mTORC1, which promotes cell survival, anabolic metabolism, and proliferation. Within the TME, these alterations further facilitate immune evasion and sustain a pro‐tumorigenic niche, collectively driving tumor progression and resistance to therapy.^[^ ^111^, ^127^ ^]^

### 2.3 Other Metabolic Reprogramming

In addition to alterations in glucose and amino acid metabolism, cancer cells undergo reprogramming of several other key metabolic pathways to sustain their rapid growth, survival, and ability to evade therapeutic interventions. These metabolic changes include shifts in lipid metabolism,^[^ ^128^, ^129^ ^]^ nucleotide metabolism,^[^ ^31^, ^130^ ^]^ and the regulation of mitochondrial function,^[^ ^131^, ^132^ ^]^ all of which support the anabolic and bioenergetic demands of proliferating tumor cells. Lipid metabolism, for example, provides essential components for membrane biogenesis and signaling, while nucleotide metabolism is crucial for DNA and RNA synthesis in rapidly dividing cells. Furthermore, mitochondrial reprogramming enables cancer cells to adapt to varying oxygen conditions and optimize energy production. This section explores the broader landscape of metabolic reprogramming in cancer, highlighting the diverse metabolic pathways that contribute to tumorigenesis and their potential as therapeutic targets.

#### 2.3.1 Lipid Metabolism

In rapidly proliferating tumor cells, lipid synthesis is reprogrammed to meet the increased demand for cell membrane components during cell division.^[^ ^133^ ^]^ Key enzymes involved in lipid metabolism, such as fatty acid synthase (FASN) and acyl‐CoA synthetase, are often upregulated in various cancers, promoting the de novo synthesis of fatty acids and phospholipids.^[^ ^134^, ^135^, ^136^, ^137^ ^]^ These lipids are vital not only for membrane structure but also for their role in cellular signaling pathways. Lipid‐derived second messengers, such as phosphoinositides, sphingolipids, and diacylglycerol, regulate crucial cellular processes like survival, migration, invasion, and metastasis, thereby supporting tumor progression.^[^ ^138^ ^]^

Lipid metabolic reprogramming in the TME not only supports tumor cell survival and growth under metabolic stress but also profoundly influences immune cell function. Tumor cells upregulate lipid biosynthesis via mTORC1‑SREBP1 signaling, while simultaneously suppressing lipid catabolism by downregulating AMPK activity. This results in the accumulation of lipids, including fatty acids and cholesterol, which act as both energy sources and signaling molecules that facilitate resistance to apoptosis and metabolic adaptation under hypoxia or nutrient deprivation.^[^ ^128^, ^139^ ^]^ Within the TME, tumor‐derived lipids and metabolic byproducts are taken up by infiltrating immune cells through transporters such as CD36 and fatty acid‐binding proteins (FABP4/5).^[^ ^140^ ^]^ In CD8⁺ T cells, excess lipid uptake impairs mitochondrial function and promotes lipid peroxidation, leading to T cell exhaustion and reduced cytotoxicity.^[^ ^141^ ^]^ Lipid overload also disrupts TCR signaling by altering membrane lipid rafts. In macrophages, lipid‐rich environments skew polarization toward the M2‐like (immunosuppressive) phenotype, partly via PPARγ activation, which supports tumor‐promoting inflammation and suppresses antigen presentation.^[^ ^142^ ^]^ Moreover, stromal cells such as cancer‐associated adipocytes and CAFs actively contribute to lipid remodeling by releasing free fatty acids and modulating extracellular matrix stiffness, respectively.^[^ ^140^ ^]^ These lipid‐rich signals reinforce immunosuppression and tumor progression.^[^ ^143^, ^144^ ^]^ Together, these metabolic alterations establish a lipid‐driven immunosuppressive niche, highlighting lipid metabolism as a promising therapeutic target to restore antitumor immunity.^[^ ^145^ ^]^

#### 2.3.2 Nucleotide Metabolism

Nucleotide metabolism plays a central role not only in supporting tumor proliferation and DNA repair but also in modulating immune responses within the TME.^[^ ^31^, ^146^ ^]^ In cancer cells, de novo purine and pyrimidine biosynthesis is upregulated to meet the elevated demand for DNA and RNA precursors. Key enzymes such as thymidylate synthase (TYMS) and ribose‐5‐phosphate isomerase are frequently overexpressed, facilitating continuous nucleotide production for rapid cell division.^[^ ^147^, ^148^, ^149^, ^150^, ^151^ ^]^ These pathways depend on amino acids, including glutamine, glycine, aspartate, and serine, which supply carbon and nitrogen atoms for nucleotide backbone synthesis.^[^ ^152^, ^153^ ^]^

Beyond their roles in cancer cells, nucleotide metabolism also critically influences immune cell function. Upon activation, T cells require robust nucleotide biosynthesis to support DNA replication, RNA transcription, and cytokine production during clonal expansion. Impaired purine metabolism can limit T cell proliferation, while one‐carbon metabolism, driven by serine and methionine, is essential for epigenetic remodeling and effector gene expression.^[^ ^152^, ^154^ ^]^ Within the TME, extracellular ATP released by dying or stressed tumor cells is rapidly hydrolyzed to adenosine through the sequential actions of CD39 and CD73 ectonucleotidases. Accumulated adenosine activates the A2A receptor (A2AR) on T cells, suppressing TCR signaling, reducing IFN‐γ production, and promoting T cell exhaustion and Treg induction—constituting a major axis of immunosuppression.^[^ ^155^, ^156^ ^]^ Additionally, nucleotide pool imbalances in immune cells under nutrient stress or tumor‐induced metabolic competition can lead to DNA replication stress and ATR/Chk1 pathway activation, culminating in cell cycle arrest or apoptosis.^[^ ^157^ ^]^ These processes compromise effector T cell function and facilitate immune escape.^[^ ^158^ ^]^

In summary, dysregulated nucleotide metabolism in tumors not only supports malignant growth and therapy resistance but also contributes to immune evasion by disrupting T cell proliferation, function, and survival. Targeting nucleotide synthesis or adenosine signaling pathways, therefore, represents a promising strategy to enhance the efficacy of immunotherapy by reprogramming the immunometabolic landscape.^[^ ^31^ ^]^

#### 2.3.3 Regulation of Mitochondrial Function

Mitochondria are central regulators of cellular metabolism, integrating energy production, redox homeostasis, and cell fate decisions in both tumor and immune cells.^[^ ^131^, ^132^, ^159^ ^]^ In cancer cells, mitochondrial reprogramming facilitates anabolic biosynthesis, maintains redox balance, and confers resistance to apoptosis.^[^ ^160^, ^161^, ^162^ ^]^ Although tumors often exhibit reduced oxidative phosphorylation in favor of aerobic glycolysis, mitochondria remain indispensable for generating TCA cycle intermediates that support macromolecule synthesis and survival under metabolic stress.^[^ ^21^ ^]^

In effector T cells, mitochondrial metabolism is essential for sustaining clonal expansion, cytokine production, and memory differentiation.^[^ ^163^, ^164^ ^]^ Upon activation, CD8⁺ T cells upregulate oxidative phosphorylation and increase mitochondrial biogenesis to meet heightened energy demands. Disruption of mitochondrial integrity caused by hypoxia, nutrient deprivation, or elevated ROS compromises mitochondrial membrane potential (ΔΨm), thereby promoting T cell exhaustion and apoptosis.^[^ ^165^ ^]^ Moreover, immunosuppressive metabolites in the TME, such as lactate and adenosine, inhibit mitochondrial respiration and FAO in T cells, diminishing their cytotoxic capacity. Mitochondrial dynamics—namely, the balance between fission and fusion—also play a crucial role in immune cell function.^[^ ^166^ ^]^ Mitochondrial fusion enhances cristae structure and oxidative phosphorylation efficiency and is required for memory T cell formation, whereas sustained mitochondrial fission is associated with T cell senescence and dysfunction. In the TME, competition for oxygen and nutrients perturbs these dynamics in TILs, further undermining immune responses. Innate immune cells, such as macrophages and dendritic cells, also depend on mitochondrial metabolism for functional polarization. In M1 macrophages, impaired electron transport chain (ETC) activity results in succinate accumulation, which stabilizes HIF‐1α and drives pro‐inflammatory IL‐1β production. Conversely, chronic mitochondrial stress or altered dynamics favor M2‐like polarization, reinforcing immune suppression within the TME.^[^ ^167^ ^]^

In summary, mitochondrial metabolism and dynamics not only sustain tumor cell viability but also govern immune cell activation, persistence, and effector function. Targeting mitochondrial pathways—by enhancing mitochondrial fitness in T cells or disrupting oxidative phosphorylation in tumor cells—offers a promising strategy to reprogram the TME and potentiate antitumor immunity.^[^ ^168^, ^169^ ^]^

### 2.4 Impact of Metabolic Reprogramming on the TME

**Figure 2:**
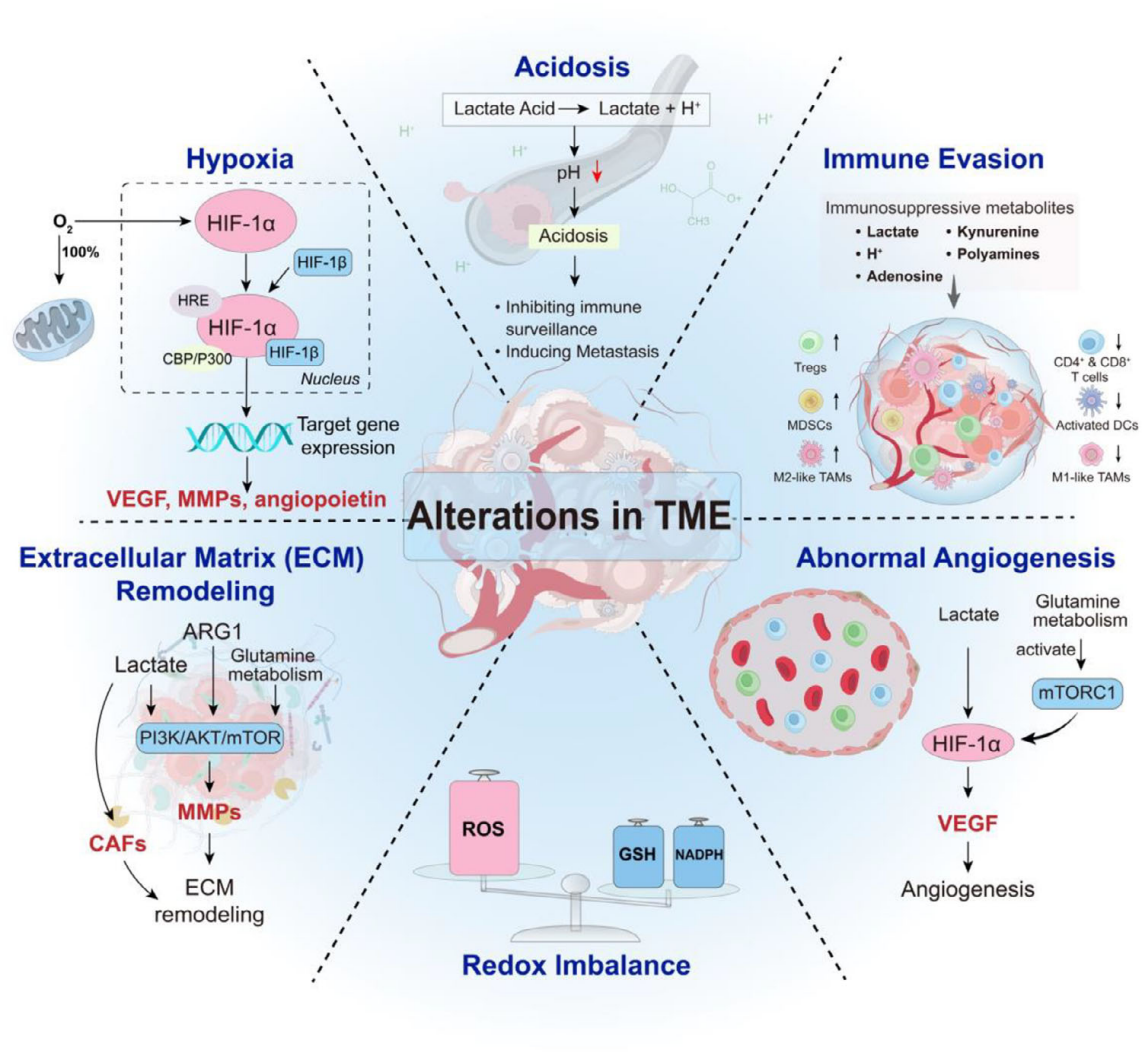
Key Alterations in the TME. This figure illustrates the major changes within the TME, including hypoxia, acidosis, redox imbalance, ECM remodeling, abnormal angiogenesis, and immune evasion. Together, these changes drive tumor progression, promote immune suppression, and contribute to resistance against therapeutic interventions.

Metabolic reprogramming in tumor cells profoundly reshapes the TME, promoting immunosuppression and therapeutic resistance.^[^ ^24^, ^109^, ^170^ ^]^ Oncogenic signaling pathways such as PI3K/Akt/mTOR, MYC, and KRAS drive enhanced aerobic glycolysis, glutamine uptake, de novo lipid synthesis, and mitochondrial remodeling,^[^ ^171^ ^]^ leading to aggressive consumption of nutrients like glucose, glutamine, serine, and arginine. This nutrient depletion impairs the metabolic fitness and effector functions of infiltrating immune cells, particularly CTLs.^[^ ^10^, ^172^ ^]^ Simultaneously, rapid tumor proliferation often exceeds vascular supply, resulting in hypoxia and stabilization of HIFs, which further amplify glycolysis and lactate production. The accumulation of lactate and the resulting acidosis suppress CTL and NK cell activity, facilitate the recruitment and functional stability of Tregs, and drive TAM polarization toward an immunosuppressive M2‐like phenotype.^[^ ^26^, ^88^ ^]^ Additional mechanisms, including glutamine catabolism,^[^ ^95^, ^127^ ^]^ arginine depletion by ARG1‐expressing myeloid cells,^[^ ^98^ ^]^ and lipid metabolic reprogramming,^[^ ^128^, ^138^ ^]^ further impair immune surveillance. Lipid accumulation within TAMs promotes their polarization toward an immunosuppressive M2‐like phenotype, thereby facilitating tumor progression.^[^ ^173^, ^174^ ^]^ Concurrently, lipid‐derived mediators, notably PGE_2_, impede the maturation and functional capacity of dendritic cells,^[^ ^175^ ^]^ leading to compromised antigen presentation and attenuated T cell activation. Moreover, immunosuppressive metabolites—such as lactate, adenosine (via CD39/CD73), and Kyn (via IDO1/TDO2)—engage receptors including GPCRs and AhR to drive T cell exhaustion and myeloid suppression.^[^ ^17^, ^71^, ^172^ ^]^ As illustrated in **Figure** **2**, key consequences include hypoxia, acidosis, redox imbalance, ECM remodeling, abnormal angiogenesis, and immune evasion. Targeting tumor‐specific metabolic vulnerabilities—such as GLUT1, glutaminase (GLS), FASN, or immunosuppressive metabolites—offers a promising strategy to remodel the TME and restore antitumor immunity.^[^ ^176^ ^]^ However, due to overlapping metabolic dependencies between tumor and immune cells,^[^ ^17^ ^]^ the development of selective delivery systems (e.g., nanoparticle‐based platforms) and rational combination therapies is essential.

## 3 Nanomaterial‐Based Therapies to Modulating Immunometabolism

Recent advances in cancer therapy have positioned nanomaterial‐based strategies as powerful tools for modulating the tumor immune microenvironment and reprogramming immunometabolic responses.^[^ ^59^ ^]^ By targeting key metabolic pathways within tumor cells and immune cells, these therapies aim to restore or enhance antitumor immunity.^[^ ^10^, ^172^ ^]^ Nanomaterails offer distinct advantages in drug delivery due to their ability to encapsulate diverse therapeutic agents, selectively accumulate in diseased tissues, and enable controlled or stimuli‐responsive release.^[^ ^53^, ^228^ ^]^

**Figure 3:**
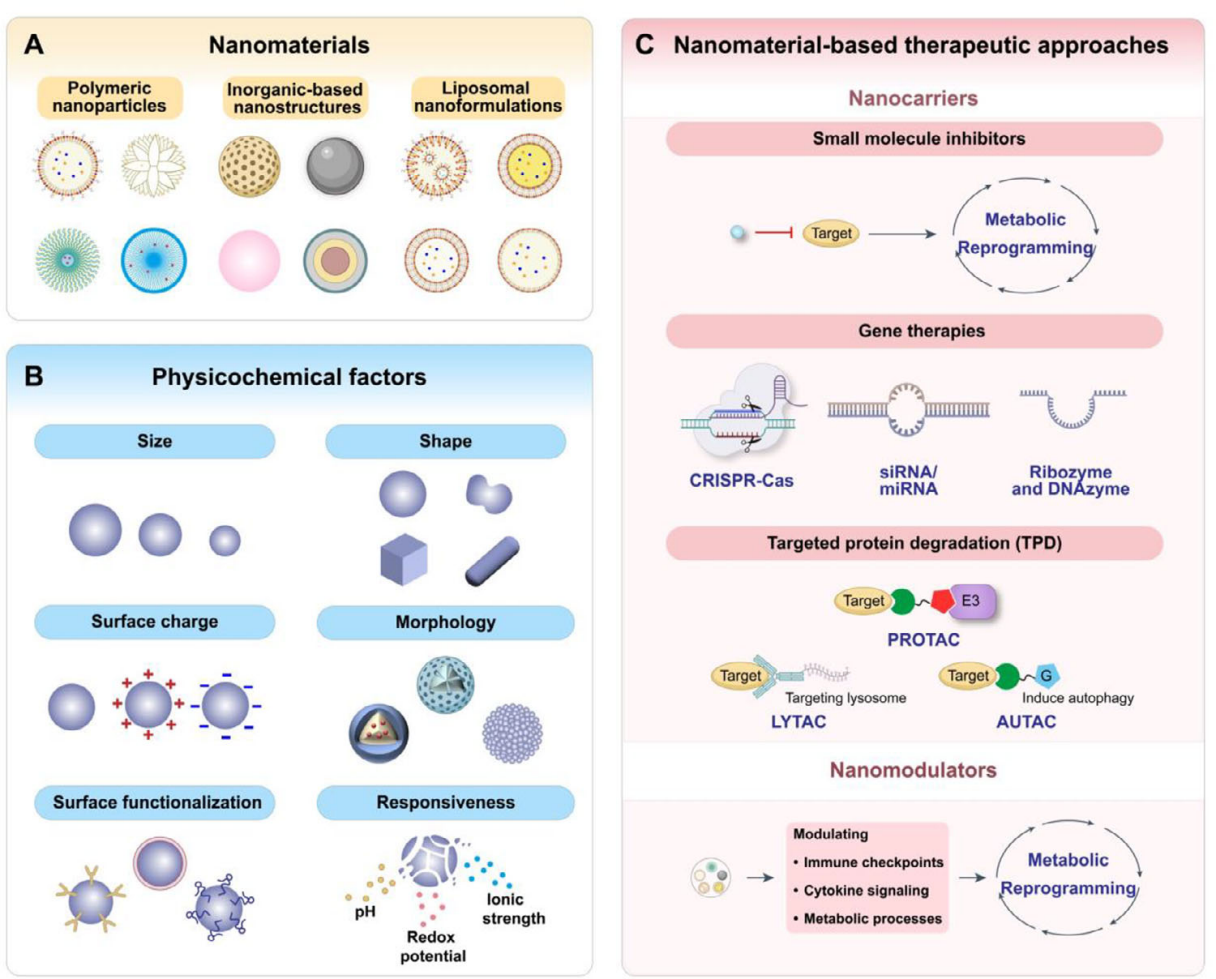
A) Representative classes of nanomaterials used to modulate immunometabolism, including polymeric nanoparticles, inorganic‐based nanostructures, and liposomal nanoformulations. B) Key physicochemical parameters of nanomaterials, such as size, shape, surface charge, morphology, surface functionalization, and responsiveness to environmental stimuli, can be rationally engineered to support immunometabolism‐targeted therapeutic strategies. C) Overview of nanomaterial‐based therapeutic approaches aimed at reprogramming the TME to enhance antitumor immune responses.

Engineered nanomaterials can influence immune metabolism directly or indirectly by modulating immune checkpoints, cytokine signaling, and metabolic processes such as glycolysis and oxidative phosphorylation. Moreover, combining nanomaterial‐based strategies with chemotherapy, radiotherapy, or immunotherapy offers a promising route to overcome immune suppression and tumor‐driven metabolic reprogramming.^[^ ^53^, ^59^ ^]^ Representative classes of nanomaterials used for immunometabolic modulation include polymeric nanoparticles, inorganic‐based nanostructures, and liposomal nanoformulations (**Figure** **3**). Their biological activity and therapeutic efficacy are governed by a range of physicochemical properties, such as particle size, shape, surface charge, morphology, and core composition, as well as surface functionalization, hydrophobicity/hydrophilicity, and responsiveness to environmental stimuli (e.g., pH, redox potential, ionic strength).^[^ ^47^, ^229^ ^]^ In addition to these inherent features, rational design strategies—such as core–shell architectures, stimuli‐responsive linkers, and ligand‐directed surface modifications—are frequently employed to enhance cell targeting and metabolic pathway specificity. These design elements can be optimized to improve biodistribution, cellular internalization, TME penetration, and selective uptake by immune subsets. Common targeting mechanisms include receptor‐mediated endocytosis (e.g., via CD44) and environmentally responsive release systems, enabling spatiotemporal control over therapeutic delivery within the TME. Comprehensive design frameworks for immunometabolism‐targeting nanomaterials have been systematically described in prior reviews.^[^ ^59^, ^60^, ^230^ ^]^ This section explores the expanding field of nanomaterial‐based immunometabolic modulation, with a focus on how these strategies are reshaping cancer therapy. In particular, we highlight recent advances in nanomaterials that function not only as delivery vehicles for small‐molecule inhibitors, gene therapies, and targeted protein degraders, but also as nanomodulators capable of reprogramming the TME to enhance antitumor immunity.

### 3.1 Regulation of Glucose and Lactate Metabolism Pathways in Tumors

**Figure 4:**
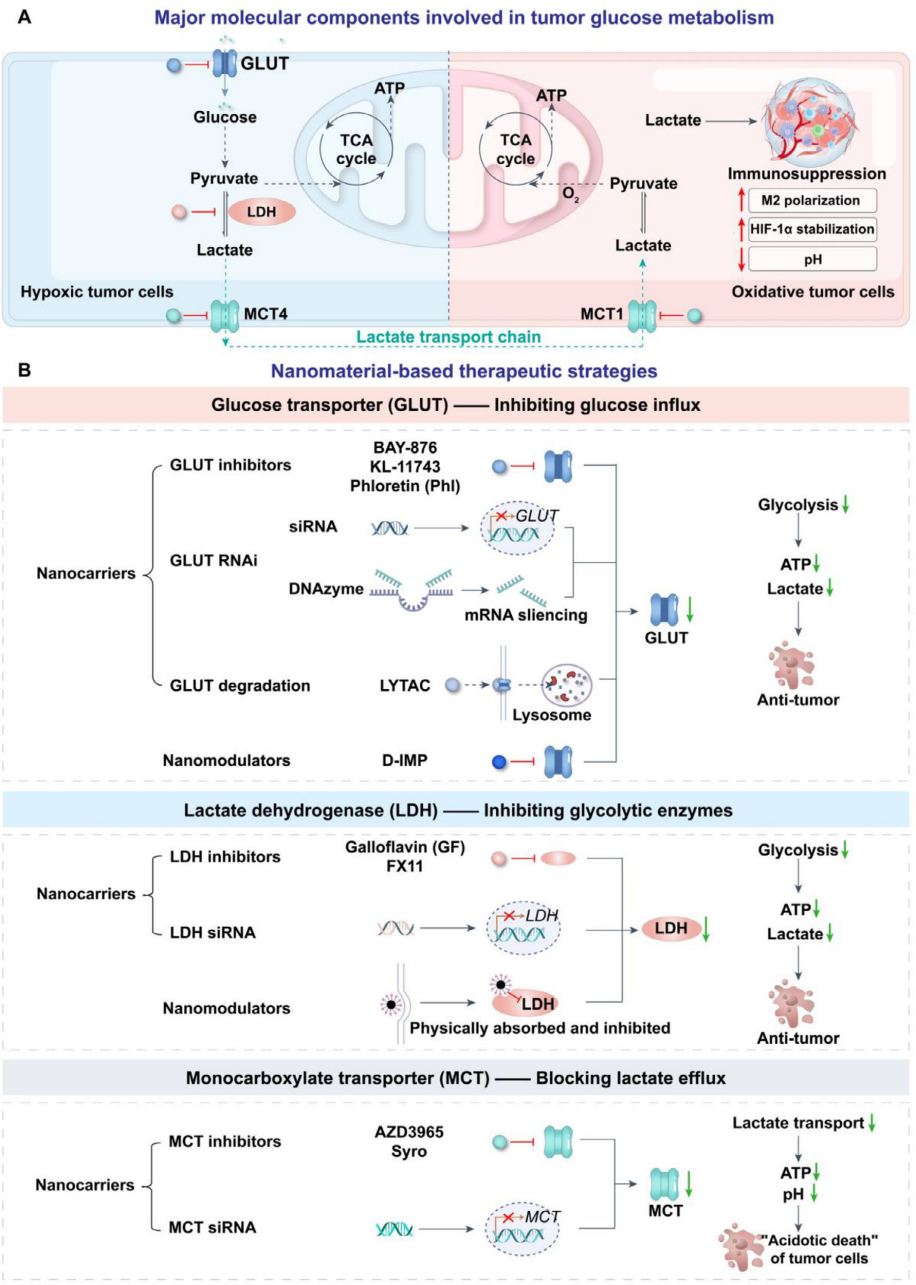
A) Major molecular components involved in tumor glucose metabolism, including GLUTs, LDH, and MCTs. B) Nanomaterial‐based therapeutic strategies targeting these components.

Tumor glucose metabolism is primarily governed by GLUTs, LDH, and MCTs, which together maintain metabolic homeostasis in the TME.^[^ ^75^, ^96^ ^]^ GLUTs facilitate glucose uptake, LDH catalyzes the conversion of pyruvate to lactate, and MCTs mediate lactate efflux and pyruvate import. This coordinated network supports tumor cell proliferation, redox balance, and adaptability under metabolic stress. However, dysregulation of this pathway, particularly the accumulation of lactate, promotes immunosuppression by impairing T cell and NK cell function and contributes to resistance to chemotherapy, radiotherapy, and immunotherapy.^[^ ^88^ ^]^ Recent CRISPR screens have revealed that tumor‐intrinsic aerobic glycolysis plays a pivotal role in immune evasion.^[^ ^231^, ^232^ ^]^ In co‐culture systems with CTLs, knockout of key glycolytic genes such as GLUT1 (Slc2a1) and GPI1 (glucose‐6‐phosphate isomerase) rendered tumor cells more vulnerable to T cell‐mediated killing. Mechanistically, this was linked to a metabolic shift toward oxidative phosphorylation and elevated mitochondrial ROS, which enhanced TNF‐α‐induced apoptosis. Notably, GLUT1 inhibition sensitized tumors to T cell attack and synergized with anti‐PD‐1 therapy in vivo through a TNF‐α‐dependent mechanism. These findings emphasize that high glucose uptake in tumors not only supports anabolic growth but also enables immune resistance by depleting nutrients and generating immunosuppressive metabolites, such as lactate. Accordingly, glucose metabolism is now recognized as a central driver of tumor immune escape. Therapeutically, targeting key regulators—GLUTs, LDH, and MCTs—represents a promising strategy to disrupt metabolic‐immune crosstalk, relieve immunosuppression, and improve treatment response.^[^ ^76^ ^]^ This section highlights nanomaterial‐based strategies for modulating GLUTs, LDH, and MCTs to reprogram tumor metabolism, enhance antitumor immunity, and optimize therapeutic efficacy (**Table** **1**, **Figure** **4**).

**Table 1:** Representative Strategies for Modulating Key Components in Glucose and Amino Acid Metabolic Pathways.

Metabolism Pathway	Targets	Nano type	Nanomodulators	Key components	Treatment Modality	Tumor types	Ref.
Glucose Metabolism	Glucose transporter (GLUT)	Polymeric nanoparticles	SPCP/CCP@Bay	Bay‐876	IO, SDT	Bladder cancer	[177]
			D‐MIP	D‐MIP	TT, MT	Breast cancer	[178]
			PG	PG	TT	Colon cancer	[179]
		Inorganic‐based nanostructures	NPs@GlcA	BAY‐876	MHT, MT	Glioblastoma	[180]
			Phl‐CaCO_3_@Lut‐Cu NPs	Phloretin	IO, MT	Melanoma	[181]
			IRF/H‐GDz/Ca	H‐GDz	GT, TT, MT	Gastric cancer	[182]
		Liposomal nanoparticles	T‐AsiG‐CPL	Glut1 siRNA, TPCA‐1	GT, TT, MT	Pancreatic cancer	[183]
	Monocarboxylate transporter (MCT)	Polymeric nanoparticles	CASN	AZD3965	PDT, IO, MT	Triple‐negative breast cancer	[184]
			NP2	Syro	CT, IO	Osteosarcoma	[185]
			PIMDQ/Syro‐RNP	Syro	IO, TT, MT	Breast cancer	[186]
			GHB NPs	siBsg	IMT, GT	Melanoma	[187]
		Inorganic‐based nanostructures	PMS	Syro	CDT, IO	Colon cancer	[188]
	Lactate dehydrogenase (LDH)	Polymeric nanoparticles	PAPEI/LDHA‐siRNA	siLDHA	CT, GT	Colorectal cancer	[189]
		Inorganic‐based nanostructures	PCGF	Galloflavin	CT, PDT, IMT	Breast cancer	[190]
			ZIAMH	ArBu	PTT, CDT, CT, MT	Hepatocellular carcinoma	[191]
			Pt@TAT/sPEG	Pt@TAT	CT, TT	Anaplastic thyroid cancer	[192]
			RM‐CDC@FX11&Ava	FX11	IO, MT	Mastadenoma, melanoma	[193]
Amino acid Metabolism (Amino Acid Transporter,Glutamine Metabolism, Arginine Metabolism, Tryptophan Metabolism)	SLC7A5/LAT1	Polymeric nanoparticles	FT‐BL@P	JPH203	GT, TT	Hepatocellular carcinoma	[194]
			LJ@AA‐NPs	JPH203	TT, MT	Breast cancer	[195]
	SLC7A11/xCT	Polymeric nanoparticles	G‐PA4/E Nanoparticles	Erastin	IO, TT, MT	Pancreatic cancer	[196]
			ECIN	Erastin	PTT, PDT	Hepatocellular carcinoma	[197]
		Inorganic‐based nanostructures	PDA‐MOF‐E‐M	Erastin	PTT, MT	Osteosarcoma	[198]
			CM‐Fe‐siR	siSLC7A11	GT, CDT	Oral squamous cell carcinoma	[199]
	SLC6A14/ATB^0,+^	Polymeric nanoparticles	LJ@AA‐NPs	AA‐NPs	TT, MT	Breast cancer	[195]
		Inorganic‐based nanostructures	αMM@PLTs	α‐MT	SDT, MT	Breast cancer	[200]
	Glutaminase (GLS)	Polymeric nanoparticles	PCB	BPTES	IO, MT	Breast cancer	[201]
			IRCB@M	CB‐839	PDT	Gastric cancer	[202]
			GelMA‐CJCNPs	C968	PDT, IO, MT	Melanoma	[203]
			FA‐DCNPs	DON	IO, TT, MT	Ovarian cancer	[204]
			PUREG4‐LA12‐anti‐GLS1‐siRNA	siGLS1	GT	Glioblastoma	[205]
		Inorganic‐based nanostructures	LDH/siRNA nanoparticles	siGLS1	GT	Pancreatic cancer	[206]
	Arginase	Polymeric nanoparticles	GDNPs	GDNPs	IO, MT	Colon cancer	[207]
			CXCR2‐NP	Ruxolitinib	IO, TT	Pancreatic ductal adenocarcinoma	[208]
		Inorganic‐based nanostructures	HN‐HFPA	L‐Nor	PDT, IO, MT	Triple‐negative breast cancer	[209]
	Ornithine decarboxylase (ODC)	Polymeric nanoparticles	Man‐NPs	DFMO	IO, TT, MT	Osteosarcoma	[210]
		Inorganic‐based nanostructures	DFMO CDs	DFMO	TT, MT	Neuroblastoma	[211]
Amino acid Metabolism (Amino Acid Transporter, Glutamine Metabolism, Arginine Metabolism, Tryptophan Metabolism)	Indoleamine 2,3‐dioxygenase (IDO)	Polymeric nanoparticles	BN@HM‐OVA	NLG919	IO, TT	T‐cell lymphoma	[212]
			NCSNPs	NLG919	IO	Melanoma	[213]
			SPN_DN_H	NLG919	SDT, IO	Pancreatic cancer	[214]
			FPND	NLG919	CT, IO	Pancreatic ductal adenocarcinoma	[215]
			PSMT NPs	1MT	CT, IO	Breast cancer	[216]
			PTX@PoxMTP NP	1MT	CT, IO	Breast cancer	[217]
			SNP	IDOi	CT, IO	Colon cancer	[218]
			LPNs	LPNs	PDT, IO	Colon cancer	[219]
			GM NPs	GN	IO, TT	Breast cancer	[220]
			HPCD	d‐ss‐DO	PDT, IO	Breast cancer, Melanoma	[221]
		Inorganic‐based nanostructures	CGDMRR	1MT	CT, CDT, ST, IMT	Breast cancer	[222]
			UCNP@MOF	BMS‐986205	PDT, IO	Breast cancer	[223]
			CDs‐IND	IND	IMT, TT	Breast cancer	[224]
			BMS‐SNAP‐MOF	BMS‐986205	IO, TT	Triple‐negative breast cancer	[225]
			CC@SiO_2_‐PLG	NLG919	PDT, IO	Breast cancer	[226]
		Liposomal nanoparticles	pDNA + siRNA LNPs	siIDO1	GT, IO	Melanoma	[227]

CDT: Chemo dynamic Therapy; CT: Chemo Therapy; GT: Gene Therapy; IO: Immunotherapy; IMT: Immunometabolic Therapy; MHT: Magnetic Hyperthermia Therapy; MT: Metabolic Therapy; PDT: Photodynamic Therapy; PTT: Photothermal Therapy; RT: Radiation Therapy; SDT: Sonodynamic Therapy; ST: Starvation Therapy; TT: Targeted Therapy

#### 3.1.1 From GLUT Inhibition to Integrated Nanotherapeutics

GLUTs are membrane proteins that transport glucose and other monosaccharides across cell membranes, playing a central role in cellular energy homeostasis.^[^ ^233^ ^]^ In cancer, GLUTs—especially GLUT1 and GLUT3—are frequently overexpressed to meet the heightened glucose demands of rapidly proliferating cells.^[^ ^62^, ^96^, ^234^, ^235^ ^]^ Elevated GLUT expression correlates with poor prognosis and increased tumor aggressiveness.

Given the central role of GLUT1 in tumor metabolism, inhibitors targeting this transporter have emerged as promising anticancer agents.^[^ ^62^ ^]^ By blocking glucose uptake, these inhibitors aim to starve tumor cells of critical energy and biosynthetic precursors, limiting proliferation and survival. Small‐molecule GLUT1 inhibitors such as WZB117, BAY‐876, and KL‐11743 have been extensively evaluated in preclinical studies.^[^ ^236^, ^237^, ^238^ ^]^ Monoclonal antibodies targeting GLUT1 are also under investigation; they bind to the transporter's extracellular domain, blocking glucose transport and potentially enhancing immune‐mediated tumor killing.^[^ ^239^ ^]^ However, GLUT1 inhibition alone may not suffice, as cancer cells can shift to alternative metabolic pathways.^[^ ^240^ ^]^ Consequently, combination strategies are being pursued to enhance therapeutic outcomes, with growing interest in integrating nanotechnology to improve delivery and efficacy.

Numerous studies have explored the integration of nanomaterials, including inorganic‐based nanostructures, polymeric nanoparticles, and liposomal nanoparticles, with GLUT1 small‐molecule inhibitors to enhance the efficacy of combination cancer therapies (Table 1).^[^ ^177^, ^180^, ^241^ ^]^ For example, a nanomedicine (SPCP/CCP@Bay) was developed by combining a degradable sonodynamic pseudo‐conjugated polymer (SPCP) with a cystine‐containing polymer (CCP) to co‐deliver the GLUT1 inhibitor Bay‐876. Upon ultrasound activation, SPCP/CCP@Bay facilitates the controlled release of Bay‐876, disrupts intracellular redox homeostasis, and liberates cystine from CCP, collectively inducing disulfidptosis. In addition, this system triggers immunogenic cell death (ICD) and synergizes with anti‐PD‐1 therapy to enhance antitumor immunity.^[^ ^177^ ^]^ Another example is a dual‐template molecularly imprinted polymer (D‐MIP) designed to target both GLUT1 and HK2. The polymer's high specificity (imprinting factors of 2.1 and 2.5 for GLUT1 and HK2, respectively) allows it to inhibit glucose uptake and phosphorylation, suppressing glycolysis. In vitro and in vivo studies, including MCF‐7 cell models and tumor‐bearing mice, demonstrated D‐MIP's capacity to reduce glycolytic flux, lower downstream metabolites like glucose‐6‐phosphate (6PG) and lactate (LA), induce apoptosis, and achieve potent antitumor effects with good biocompatibility.^[^ ^178^ ^]^

Beyond direct inhibition, strategies targeting GLUT biosynthesis or degradation are gaining attention.^[^ ^179^, ^242^, ^243^, ^244^ ^]^ For example, a programmable nanosystem was developed to address both pancreatic ductal adenocarcinoma (PDAC) cells and pancreatic stellate cells (PSCs). PDAC cells overexpress GLUT1 and transferrin receptor (CD71), while PSCs support the tumor metabolically. A TME‐responsive liposome co‐delivering the NF‐κB inhibitor TPCA‐1 and CD71 aptamer‐conjugated GLUT1 siRNA reprogrammed PSCs, reduced their metabolic support, and enhanced siRNA delivery. This dual‐targeted approach shifted PDAC metabolism from glycolysis toward oxidative phosphorylation, improving therapeutic efficacy in an orthotopic xenograft model.^[^ ^183^ ^]^ Additionally, a glycopeptide‐based strategy used a glycosylated peptide (PG) with enhanced cancer cell permeability to induce GLUT1 degradation. PG accumulated in lysosomes, self‐assembled into aggregates, and triggered GLUT1 degradation, ultimately leading to cancer cell death. In vivo, PG significantly inhibited tumor growth with minimal toxicity to major organs.^[^ ^179^ ^]^ Another promising approach involves the use of metal–nucleic acid hybrid nanostructures (MNFs). For example, a HER2‐targeted DNAzyme nanodevice was developed by integrating a HER2‐specific aptamer with a Ca^2^⁺‐dependent, GLUT1‐cleaving DNAzyme. Through Ca^2^⁺‐assisted self‐mineralization, this system generated interferon regulatory factor‐1 (IRF‐1)‐loaded mineralized nanoflowers (MNFs) capable of selectively targeting HER2‐positive gastric cancer cells and silencing GLUT1 mRNA. Silencing GLUT1 effectively inhibited glucose uptake, disrupting the pentose phosphate pathway and reducing NADPH production. This metabolic disruption impaired cysteine biosynthesis and depleted GSH, thereby disturbing redox homeostasis and promoting ROS accumulation. The resulting oxidative stress, further intensified by mitochondrial calcification, led to significant DNA damage. Concurrently, the delivery of IRF‐1 via MNFs downregulated RAD51 expression, a key component of the homologous recombination (HR) DNA repair pathway. When combined with GLUT1 silencing‐induced ATP depletion, this dual strategy further compromised DNA repair capacity and sensitized tumor cells to ROS‐mediated cytotoxicity. Overall, this approach synergistically disrupted GSH/ROS balance, inhibited HR‐dependent DNA repair, and amplified oxidative DNA damage, achieving tumor inhibition rates of up to 90% in preclinical models.^[^ ^182^ ^]^

The strategies discussed hold strong potential to advance personalized cancer therapy by targeting tumor metabolism, thereby limiting disease progression and reducing metastasis and recurrence. However, further investigation into GLUT1 regulation through nanomaterial‐based approaches—particularly in combination with chemotherapy, radiotherapy, or immunotherapy—is essential for enhancing therapeutic efficacy, overcoming resistance, and improving patient outcomes.

#### 3.1.2 Extending Metabolic Disruption: LDH as a Central Node

LDH catalyzes the final glycolytic step, converting pyruvate to lactate while regenerating NAD⁺, and plays a key role in supporting tumor proliferation under aerobic glycolysis.^[^ ^63^, ^88^, ^93^ ^]^ In tumors, LDH is upregulated, promoting anaerobic glycolysis even in oxygen‐rich conditions and contributing to tumor progression.^[^ ^245^, ^246^ ^]^

LDH exists in five isoforms (LDH1 to LDH5) formed by different combinations of two subunits, LDH‐A and LDH‐B.^[^ ^93^ ^]^ These isoforms exhibit tissue‐specific expression patterns. Tumors often have elevated levels of LDH‐A, which is associated with glycolytic activity and lactate production.^[^ ^247^ ^]^ In contrast, LDH‐B is more prevalent in tissues relying on oxidative phosphorylation, such as the heart and muscle. LDH‐A inhibition has garnered more attention in cancer therapy due to its central role in aerobic glycolysis. Inhibiting LDH‐A can reduce lactate production, thereby decreasing the acidic environment in the TME and reducing cancer cell survival.^[^ ^88^ ^]^ LDH‐B, however, has been implicated in lactate utilization and energy production in some tissues, making selective inhibition of LDH‐A more appealing for therapeutic purposes.^[^ ^245^, ^248^, ^249^ ^]^ Recent developments also include PROTAC‐based degraders to further suppress LDH activity.^[^ ^246^ ^]^ While preclinical studies show LDH inhibition slows tumor growth and enhances therapy response, challenges remain, including selectivity, toxicity, and patient stratification. To address these, nanomaterial‐based strategies are increasingly explored to improve delivery and optimize combination treatments (Table 1).^[^ ^250^, ^251^ ^]^

Galloflavin (GF), a natural polyphenol and LDH inhibitor, suppresses the conversion of pyruvate to lactate, thereby reducing lactic acid production.^[^ ^252^ ^]^ A metal–phenolic coordination‐based nanocomplex loaded with GF was developed to impair LDH activity and attenuate lactic acid accumulation, ultimately alleviating the acidic and immunosuppressive TME. Additionally, the nanocomplex co‐delivered carnosic acid (a natural polyphenol) and PEG‐Ce6, a synthetic polyphenol‐derived photosensitizer. Upon laser irradiation, these agents induced ICD, further activating the immune system and enhancing immune cell recruitment and infiltration into tumor tissues. In a murine breast cancer model, this nanocomplex‐based combination therapy effectively remodeled the TME and elicited robust antitumor immune responses.^[^ ^190^ ^]^ In another study, a copper‐coordinated nanoassembly (Cu‐GM) was developed by integrating the LDH inhibitor GF and the immune checkpoint inhibitor myricetin (MY) to enhance cuproptosis‐based immunotherapy. Cu‐GM was activated by elevated intracellular GSH, triggering the release of Cu⁺, which induced the aberrant aggregation of lipoylated proteins and the degradation of iron–sulfur cluster proteins, leading to proteotoxic stress and cuproptosis. Concurrently, GF inhibited glycolysis, further amplifying cuproptosis and reducing lactate accumulation, thereby alleviating lactate‐mediated immunosuppression in the TME. The resulting tumor cell death induced ICD, promoting antitumor immune activation, which was further potentiated by MY‐mediated immune checkpoint blockade.^[^ ^253^ ^]^ Additionally, researchers employed vesicular cationic lipid‐assisted nanoparticles to deliver siRNA targeting the LDHA gene in tumors. LDHA silencing significantly reduced lactate production and neutralized tumor acidity, leading to an increased intratumoral CD8⁺ T cell to Treg ratio. This strategy shows promise for enhancing T cell‐mediated antitumor immunity.^[^ ^250^ ^]^ Inorganic‐based nanoparticles have been shown to disrupt the structural integrity and biological activity of natural enzymes through various physical interactions, including hydrophobic effects, π–π stacking, and electrostatic forces.^[^ ^254^, ^255^ ^]^ Based on this principle, researchers developed a pH‐responsive, nucleus‐targeting platinum nanocluster (Pt@TAT/sPEG) to simultaneously inhibit LDH activity and enhance DNA damage. Pt@TAT/sPEG physically adsorbed and suppressed LDH activity, thereby reducing lactate production and downregulating lactate‐mediated nucleotide excision repair (NER) signaling, leading to increased apoptosis in anaplastic thyroid carcinoma (ATC) cells. In addition, Pt@TAT/sPEG directly inhibited the NER pathway, further promoting tumor cell death. In an orthotopic ATC xenograft model, Pt@TAT/sPEG demonstrated improved tumor inhibition compared to Pt@sPEG and cisplatin. This nanoplatform offers a promising strategy for concurrently targeting glycolysis and DNA repair, facilitating metabolic reprogramming and enhancing chemotherapy efficacy.^[^ ^192^ ^]^

Together, these nanotechnology‐based LDH‐targeting approaches, whether through small‐molecule inhibitors, siRNA, or multifunctional nanoplatforms, show substantial potential in preclinical models, effectively suppressing tumor growth, enhancing immune infiltration, and sensitizing tumors to immunotherapy. However, challenges such as limited tumor selectivity, heterogeneous LDH expression, and inefficient nanoparticle delivery within the TME hinder consistent therapeutic outcomes. Moreover, adaptive resistance via metabolic compensation and immune‐mediated clearance of nanocarriers further complicates clinical translation.

#### 3.1.3 Linking Lactate Transport Control: MCT as a Therapeutic Target

MCTs facilitate the bidirectional transport of lactate, pyruvate, and ketone bodies across cell membranes, playing essential roles in energy metabolism and reprogramming.^[^ ^256^ ^]^ In tumors, MCT1 and MCT4 are critical for maintaining lactate and pyruvate balance, sustaining glycolytic flux, and supporting the Warburg effect.^[^ ^96^, ^257^ ^]^ MCT‐mediated lactate efflux prevents acid buildup in glycolytic tumors and regulates metabolic exchanges between tumor and stromal cells, contributing to tumor progression and immune evasion. Given their central metabolic role, MCTs have emerged as promising targets for improving cancer therapies.^[^ ^96^, ^257^ ^]^

Several MCT inhibitors (including MCT1/MCT2 inhibitor AR‐C155858,^[^ ^257^ ^]^ MCT1 inhibitor SR13800,^[^ ^258^ ^]^ and AZD3965^[^ ^259^ ^]^) have demonstrated promising preclinical activity. AZD3965, a well‐known MCT1 inhibitor, impairs pyruvate uptake, disrupting oxidative metabolism in tumor cells, leading to cell death and sensitizing tumors to chemotherapy and radiotherapy. It has shown efficacy in solid tumors and lymphomas and is currently in clinical trials.^[^ ^260^, ^261^, ^262^ ^]^ Syrosingopine (Syro) is an MCT1/4 inhibitor that blocks lactate efflux, causing intracellular acidification and tumor cell death.^[^ ^188^, ^263^ ^]^ In addition to their direct cytotoxic effects, MCT inhibitors may modulate the tumor immune microenvironment. Specifically, MCT4 inhibition limits lactate export from tumor cells, thereby reducing extracellular lactate accumulation. High lactate levels in the TME suppress effector T cell activity and promote the polarization of TAMs toward an M2‐like immunosuppressive phenotype, facilitating immune evasion. By alleviating lactate‐induced immunosuppression, MCT4 inhibition may enhance the efficacy of immunotherapies, including immune checkpoint blockade.^[^ ^264^, ^265^ ^]^ Collectively, these findings highlight MCTs as attractive therapeutic targets. Inhibition of MCTs, particularly in combination with chemotherapy, radiotherapy, or immunotherapy, offers a promising strategy to disrupt tumor metabolism and overcome immune resistance.^[^ ^264^, ^266^, ^267^, ^268^ ^]^

To address challenges related to selectivity, tumor heterogeneity, and resistance mechanisms, nanomaterial‐based strategies have been developed to enhance the delivery and efficacy of MCT inhibitors while minimizing off‐target effects.^[^ ^251^, ^269^ ^]^ For example, triple‐negative breast cancer (TNBC), often resistant to ICB due to a hyperacidic, immunosuppressive TME, was targeted using a carrier‐free photodynamic bioregulator (CASN) composed of Chlorin e6 and AZD3965. CASN‐mediated photodynamic therapy (PDT) not only inhibited primary tumor growth and induced ICD but also suppressed MCT1‐dependent lactate efflux, reducing Treg generation and M2 macrophage polarization. Combined with ICB, this approach markedly enhanced CTL recruitment, improving antitumor immunity and controlling TNBC growth and metastasis.^[^ ^184^ ^]^ Similarly, ROS‐sensitive nanoparticles co‐loaded with Syro^[^ ^185^, ^186^, ^188^ ^]^ and a cisplatin(IV) prodrug were designed for osteosarcoma treatment. These nanoparticles accumulate in tumor cells, where ROS generation amplifies the delivery and effects of both agents, inducing endoplasmic reticulum (ER) stress, triggering ICD, and stimulating adaptive immunity.^[^ ^185^ ^]^ Beyond small‐molecule inhibitors, nanocarrier‐based delivery of MCT4‐targeting siRNA has emerged as an alternative strategy.^[^ ^270^, ^271^ ^]^ For example, phenylboronic acid‐ and pyridine‐modified poly(amidoamine) dendrimer/copper(II) complexes (D–Cu complexes) were developed to deliver MCT4‐targeting siRNA (siMCT4) and disrupt the tumor lactate shuttle. These D–Cu complexes exhibited a copper(II)‐mediated chemodynamic effect and demonstrated T1‐weighted magnetic resonance imaging capability (*r*_1_ relaxivity = 1.19 mM^−1^·s^−1^), enabling efficient intracellular siMCT4 delivery to inhibit lactate efflux in tumor cells. In combination with a CD11b immune agonist, treatment with D–Cu/siMCT4 polyplexes in a murine breast tumor model alleviated local TME immunosuppression and significantly inhibited both primary tumor growth and lung metastasis.^[^ ^271^ ^]^

Despite the therapeutic promise of targeting MCTs, nanomaterial‐based strategies face several challenges that must be addressed for successful clinical translation. First, achieving selective delivery of MCT inhibitors or siRNA to tumor cells, while sparing normal tissues, remains difficult due to tumor heterogeneity and the dynamic nature of the TME. Second, nanocarriers must overcome multiple physiological barriers, including abnormal tumor vasculature, elevated interstitial fluid pressure, and acidic extracellular pH, to ensure effective intratumoral accumulation and cellular internalization. Third, compensatory mechanisms such as the upregulation of alternative transporters and metabolic adaptation to lactate retention may undermine the sustained efficacy of MCT‐targeted therapies. Finally, integrating MCT inhibition with immunotherapy requires precise spatial and temporal control of drug release to reprogram the TME without compromising beneficial immune responses. Addressing these limitations through rational nanocarrier design, stimulus‐responsive release systems, and combinatorial treatment strategies will be essential to unlock the full clinical potential of MCT‐targeted nanomedicine.

### 3.2 Regulation of Amino Acid Metabolism Pathways in Tumors

Key components of the amino acid metabolic network, including amino acid transporters, GLS, arginine‐metabolizing enzymes (e.g., arginase and ornithine decarboxylase), and the IDO1–Kyn–AhR axis, serve as critical regulators of tumor metabolism and immune modulation.^[^ ^111^, ^272^, ^273^, ^274^ ^]^ Amino acid transporters facilitate the uptake of essential amino acids into tumor cells, providing building blocks for protein synthesis, nucleic acid production, and energy metabolism. These transporters not only fuel tumor cell proliferation but also regulate the availability of amino acids necessary for immune cell function, particularly T cells.^[^ ^127^, ^275^ ^]^ Dysregulation of amino acid transporters in tumors often results in an altered metabolic environment, impairing T cell activation and reducing T cell‐mediated immune responses.^[^ ^276^, ^277^ ^]^ GLS catalyzes the conversion of glutamine to glutamate, a crucial source of energy and metabolic intermediates for rapidly proliferating tumor cells.^[^ ^278^ ^]^ GLS activity also impacts immune cell metabolism by modulating glutamine availability,^[^ ^110^ ^]^ a key fuel for T cell activation and expansion. Increased glutamine metabolism in tumors can suppress T cell proliferation and function, contributing to immune evasion within the TME.^[^ ^279^ ^]^ Arginine‐related metabolic enzymes, including Arginase and Ornithine Decarboxylase, regulate arginine levels, which are vital for immune cell activation. Arginase depletes extracellular arginine, limiting its availability to T cells, which depend on arginine for activation and proliferation.^[^ ^280^, ^281^ ^]^ Ornithine Decarboxylase, involved in polyamine synthesis, influences immune cell differentiation and modulates inflammatory responses.^[^ ^282^, ^283^ ^]^ Both enzymes contribute to the immunosuppressive TME, hindering effective T cell‐mediated antitumor immunity. The IDO1–Tyn–AhR Axis is a critical pathway regulating tryptophan metabolism and immune modulation.^[^ ^273^, ^284^ ^]^ IDO1 catabolizes tryptophan into Kyn, suppressing T cell activation and promoting T cell exhaustion. This pathway is frequently upregulated in tumors to suppress immune surveillance and maintain immune tolerance.^[^ ^232^, ^285^ ^]^

**Figure 5:**
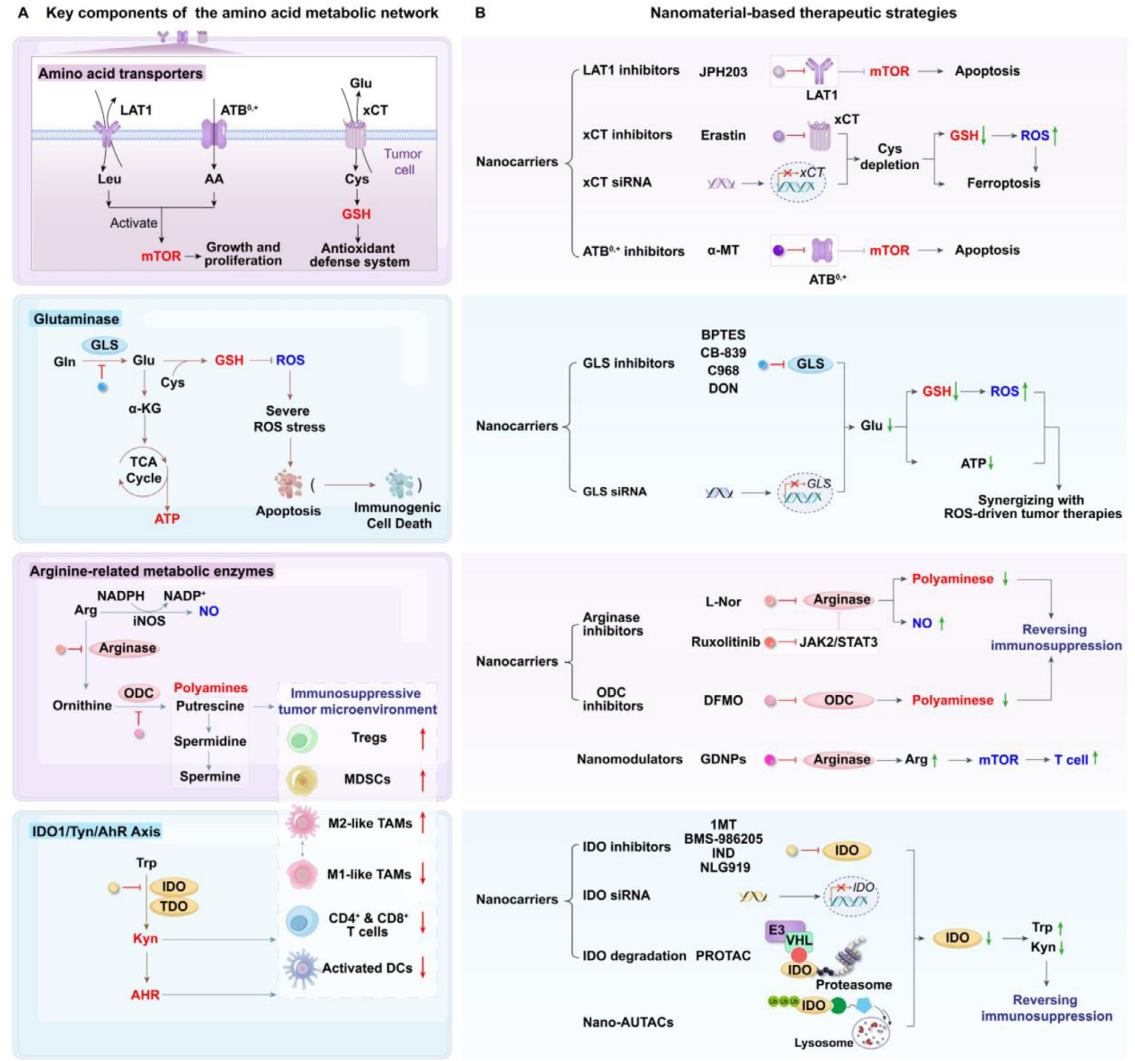
A) Key components of the amino acid metabolic network, including amino acid transporters, GLS, arginine‐metabolizing enzymes, and the IDO1–Kyn–AhR axis, function as critical regulators of tumor metabolism and immune modulation. B) Nanomaterial‐based therapeutic strategies targeting these metabolic pathways and components.

Overall, amino acid metabolism critically shapes T cell function within the TME, influencing tumor progression, immune escape, and treatment resistance. Targeting these metabolic pathways offers a promising strategy to enhance T cell‐based immunotherapy and improve clinical outcomes. **Figure** **5** summarizes nanomaterial‐based approaches for modulating amino acid transporters, GLS, arginine‐related enzymes, and the IDO1–Kyn–AhR axis to strengthen tumor therapies.

#### 3.2.1 Disrupting Amino Acid Uptake through Transporter Targeting

Tumor cells upregulate amino acid transporters—including System L (LAT1, LAT2), System X^−^ (xCT), and System Y⁺ (SLC7A1)—to meet biosynthetic and energy demands, while indirectly suppressing immune function by depleting essential nutrients in the TME.^[^ ^107^, ^127^, ^275^ ^]^

For instance, LAT1/2 mediate leucine uptake for mTORC1 activation; xCT exchanges glutamate and cysteine to maintain redox balance; and SLC7A1 controls arginine uptake, critical for T cell activation.^[^ ^107^, ^127^, ^275^, ^286^ ^]^

Inhibiting these transporters disrupts tumor metabolism and restores immune activity. Small‐molecule inhibitors (e.g., JPH203, BCH‐101, sulfasalazine), monoclonal antibodies, and gene editing approaches have shown promise, although challenges remain regarding specificity and resistance.^[^ ^287^, ^288^, ^289^, ^290^, ^291^ ^]^ Nanomaterial‐based delivery systems are emerging as potent tools to improve transporter targeting and therapeutic precision.^[^ ^200^, ^292^ ^]^ For example, in hepatocellular carcinoma, co‐delivering the LAT1 inhibitor JPH203 with a pDNA‐CHRDL‐1‐loaded nanoplatform (FT‐BL@P) synergistically suppressed tumor growth.^[^ ^194^ ^]^ Another nanoplatform (LJ@Trp‐NPs) was developed to target two key amino acid transporters—SLC6A14 (ATB⁰^,^⁺) and SLC7A5 (LAT1)—which support amino acid metabolism in many cancers. Tryptophan was used as a targeting ligand for SLC6A14‐mediated delivery of JPH203, a selective SLC7A5 inhibitor, which also led to downregulation of SLC6A14. This strategy enhanced the efficacy of the kinase inhibitor lapatinib by disrupting mTOR signaling, inducing apoptosis, and suppressing metastasis, illustrating how amino acid starvation can potentiate anticancer therapies.^[^ ^195^ ^]^ In addition, siRNA‐loaded nanoplatforms targeting SLC7A11 (also known as xCT) have been developed to induce ferroptosis. For example, a cancer cell membrane–camouflaged iron–siRNA nanohybrid (CM‐Fe‐siR) suppressed cystine uptake, leading to GSH depletion and glutathione peroxidase 4 inactivation. Concurrently, iron‐mediated Fenton reactions generated ROS, and the combined effects amplified lipid peroxidation and ferroptotic cell death, resulting in potent antitumor efficacy.^[^ ^199^ ^]^

Although nanomaterial‐based strategies targeting amino acid transporters hold significant therapeutic promise, several transporter‐specific challenges impede clinical translation. Transporters such as LAT1, xCT, and SLC6A14 are also expressed in normal proliferative tissues, increasing the risk of off‐target toxicity. Additionally, intratumoral heterogeneity and dynamic regulation of transporter expression can compromise targeting specificity and treatment consistency. Tumor cells may further evade transporter inhibition by activating compensatory amino acid uptake pathways or metabolic rewiring. These limitations underscore the need for highly selective delivery systems and a deeper mechanistic understanding of transporter biology in both cancer and immune cells to improve therapeutic precision and durability.

#### 3.2.2 Blocking Glutamine Addiction with GLS Inhibition

GLS converts glutamine to glutamate, fueling the TCA cycle, supporting biosynthesis, and maintaining redox balance. Many cancers show “glutamine addiction,” making GLS a prime therapeutic target.^[^ ^95^, ^99^, ^110^, ^278^, ^279^, ^293^ ^]^ Tumors primarily upregulate GLS1 (kidney‐type), while GLS2 (liver‐type) often plays a tumor‐suppressive role.^[^ ^294^, ^295^ ^]^

GLS inhibition deprives tumor cells of a key nutrient, leading to metabolic collapse and cell death. Several inhibitors—such as CB‐839 (in clinical trials NCT03163667, NCT03428217, and NCT03831932), BPTES, and DON—have been evaluated in preclinical models, alongside RNAi and CRISPR strategies to downregulate GLS1 expression.^[^ ^62^, ^96^, ^296^, ^297^, ^298^, ^299^, ^300^, ^301^, ^302^ ^]^ However, toxicity, heterogeneity, and delivery limitations remain significant challenges.

Nanotechnology offers solutions to improve GLS‐targeted therapy.^[^ ^201^, ^202^, ^203^, ^230^, ^303^ ^]^ For example, a biomimetic Cu‐doped polypyrrole nanoparticle system (CuP), loaded with the GLS1 inhibitor BPTES to form PCB, was developed to promote cuproptosis and modulate the tumor immune microenvironment. The PCB nanosystem consists of a BPTES/CuP core cloaked with a platelet membrane, enabling active tumor targeting following intravenous administration. PCB effectively inhibited GLS1 activity, resulting in reduced intracellular GSH levels. Western blot analysis confirmed marked depletion of Fe–S cluster proteins, including GLS, in PCB‐treated tumors. In parallel, CuP catalyzed the generation of intracellular hydrogen peroxide, exacerbating oxidative stress and triggering Cu^2^⁺‐mediated oligomerization of dihydrolipoyl transacetylase, thereby inducing cuproptosis. Notably, PCB not only suppressed primary tumor growth but also exhibited abscopal effects on distant tumors, with significantly reduced in situ GLS expression observed in treated tissues.^[^ ^201^ ^]^ Additionally, nanomaterial‐based strategies have been explored to inhibit glutamine metabolism by delivering siRNAs targeting GLS1.^[^ ^205^, ^206^ ^]^ In one study, researchers proposed a dual‐targeting approach to concurrently inhibit glucose and glutamine metabolism by co‐delivering siRNAs against mutant Kras and GLS1, thereby circumventing the toxicity associated with conventional chemotherapeutics. This strategy addresses the compensatory metabolic interplay between glucose and glutamine, resulting in enhanced antitumor efficacy. Mg–Al layered double hydroxide (LDH) nanosheets were used as siRNA carriers due to their superior delivery efficiency compared to Lipofectamine 2000. The LDH nanosheets adsorbed siKras and siGLS1 via electrostatic interactions and facilitated endosomal escape through a proton sponge effect, enhancing siRNA stability and gene silencing efficiency. The released siRNAs downregulated Kras, GLS1, and other glycolytic enzymes, leading to reduced ATP production and metabolic activity. This biocompatible LDH/siRNA nanoplatform effectively suppressed pancreatic tumor xenograft growth by inhibiting cancer cell proliferation, demonstrating strong potential as a dual‐targeted metabolic therapy.^[^ ^206^ ^]^

Despite encouraging preclinical outcomes, nanomaterial‐based strategies targeting GLS, particularly GLS1, encounter several challenges specific to this metabolic axis. Although frequently overexpressed in tumors, GLS1 is also active in normal proliferative tissues such as activated lymphocytes and intestinal epithelium, raising concerns about on‐target, off‐tumor toxicity. Moreover, metabolic redundancy enables tumor cells to circumvent GLS1 inhibition through alternative glutamine utilization pathways, including transaminase‐mediated anaplerosis and enhanced glutamate uptake, thereby sustaining TCA cycle flux and redox balance. Tumor heterogeneity further complicates therapeutic application, as variation in glutamine dependence and differential expression of GLS1 versus GLS2 across cancer types limits the generalizability of treatment responses and underscores the need for biomarker‐driven patient stratification. Finally, effective co‐targeting of compensatory pathways (e.g., glycolysis or NADPH‐generating systems) imposes additional design demands on nanocarriers, requiring precise co‐delivery and synchronized release kinetics to avoid additive toxicity. Addressing these GLS‐specific limitations is critical for advancing glutamine‐targeted nanotherapies toward clinical translation.

#### 3.2.3 Modulating Arginine Metabolism to Reprogram the TME

Arginine metabolism plays a critical role in tumor progression, immune regulation, and therapeutic resistance.^[^ ^102^, ^111^ ^]^ Within the TME, both tumor cells and immunosuppressive cells—including MDSCs and TAMs—frequently reprogram arginine metabolism to support their proliferative needs and suppress antitumor immune responses.^[^ ^29^, ^304^ ^]^ Two key enzymes in this pathway, arginase and ornithine decarboxylase (ODC), have emerged as important therapeutic targets in cancer, particularly when combined with immunotherapy or chemotherapy.

Arginase, especially the ARG1 isoform, catalyzes the conversion of L‐arginine into ornithine and urea. Although essential for nitrogen disposal, its pathological upregulation depletes extracellular arginine, impairing TCR signaling, T cell proliferation, and cytokine production.^[^ ^272^ ^]^ High ARG1 activity correlates with immune checkpoint inhibitor resistance and poor prognosis across cancers.^[^ ^111^ ^]^ Arginase inhibitors such as CB‐1158 (INCB001158) are under clinical investigation for restoring T cell function and enhancing PD‐1/PD‐L1 blockade efficacy, with preclinical studies showing synergy when combined with ICIs or chemotherapy.^[^ ^304^, ^305^, ^306^, ^307^ ^]^ ODC is the rate‐limiting enzyme in the polyamine biosynthetic pathway, converting ornithine (a product of arginase activity) into putrescine, which is subsequently converted into spermidine and spermine. These polyamines are essential for DNA replication, cell cycle progression, and cell proliferation, making ODC a critical driver of tumor growth.^[^ ^283^ ^]^ Overexpression of ODC has been observed in multiple cancers, including colorectal cancer, breast cancer, and neuroblastoma, and is often associated with poor clinical outcomes. In addition to promoting tumor growth, polyamine accumulation negatively affects antitumor immunity by impairing dendritic cell maturation and T cell activation.^[^ ^308^, ^309^ ^]^ Difluoromethylornithine (DFMO, eflornithine), an irreversible ODC inhibitor, has demonstrated anticancer activity in both preclinical and clinical settings.^[^ ^283^ ^]^ DFMO has been evaluated in clinical trials for colorectal cancer chemoprevention and in combination with other therapies.^[^ ^310^ ^]^ Current strategies pair DFMO with polyamine transport inhibitors, chemotherapy, or ICB to overcome metabolic compensation and improve therapeutic efficacy.

Targeting arginine‐related metabolic enzymes, particularly ARG1 and ODC, represents a compelling strategy to modulate the immunosuppressive TME, inhibit tumor proliferation, and enhance the efficacy of immunotherapy. Both preclinical and early‐phase clinical data support the potential of these approaches to restore arginine availability, limit polyamine biosynthesis, and boost T cell‐mediated immunity. With the advancement of nanotechnology, nanoparticle‐based delivery systems are being actively explored to improve the bioavailability, tumor‐specific accumulation, and safety profiles of arginase and ODC inhibitors. These delivery platforms offer the potential to minimize systemic toxicity, enhance tumor penetration, and facilitate co‐delivery with synergistic therapeutics such as ICIs (Table 1), thereby optimizing clinical outcomes.^[^ ^207^, ^311^, ^312^ ^]^

Nω‐hydroxy‐L‐norarginine (nor‐NOHA), a selective ARG1 inhibitor, was encapsulated in stealth liposomes to improve its pharmacokinetics and overcome limitations such as rapid clearance and low bioavailability. The liposomal formulation demonstrated sustained release, prolonged plasma half‐life, and enhanced tumor accumulation, resulting in extended ARG1 inhibition both in vitro and in vivo.^[^ ^313^ ^]^ Additionally, a hollow nanoplatform (HN‐HFPA) was engineered to enhance post‐photodynamic therapy antitumor immunity in TNBC by reprogramming TAM arginine metabolism. L‐arginine (L‐Arg), encapsulated within the hollow core, served as both a nitric oxide (NO) precursor and a substrate for arginine metabolism, while L‐norvaline (L‐Nor), an ARG1 inhibitor, was grafted onto a hyaluronic acid (HA) coating to enable TAM‐targeted delivery. In the TME, HA was degraded by hyaluronidase, and elevated GSH levels triggered nanoparticle disassembly, leading to GSH depletion and, under light irradiation, the generation of ROS. This process induced ICD and released L‐Arg, which was enzymatically converted to NO, contributing to both tumor cell killing and real‐time ultrasound imaging via gas formation. Concurrently, L‐Nor inhibited ARG1 in M2‐polarized TAMs, promoting their repolarization to an M1 phenotype, enhancing CD8⁺ T‐cell infiltration, and suppressing metastasis.^[^ ^209^ ^]^

Researchers also synthesized fluorescent carbon dots directly from DFMO, creating nanoscale, drug‐doped particles that achieved ∼60‐fold greater antitumor potency in neuroblastoma cells compared to free DFMO, due to improved uptake, nuclear accumulation, and DNA interaction.^[^ ^211^ ^]^ Another approach co‐loaded DFMO and the angiogenesis inhibitor regorafenib into mannose‐decorated PLGA–PEG nanoparticles, selectively targeting CD206⁺ M2‐like TAMs in osteosarcoma. Mannosylation enhanced M2 TAM uptake, increasing local drug concentrations. In an orthotopic model, free DFMO or regorafenib monotherapy reduced tumor growth by ∼23% and 39%, respectively, while free drug combination achieved ∼57% inhibition; in contrast, the dual‐loaded nanoparticle achieved ∼70% suppression, reprogrammed TAMs to a pro‐inflammatory M1 phenotype, suppressed angiogenesis, and minimized systemic toxicity.^[^ ^210^ ^]^

Although nanomaterial‐based strategies targeting arginine metabolism—particularly ARG1 and ODC—have demonstrated potential in reprogramming the immunosuppressive TME and augmenting immunotherapeutic responses, several target‐specific challenges persist. The physiological expression of ARG1 and ODC in nonmalignant tissues, including hepatocytes and activated immune cells, raises concerns about on‐target, off‐tumor toxicity, particularly in the absence of tumor‐restricted delivery. Moreover, tumors may develop resistance by activating compensatory pathways, such as alternative polyamine biosynthesis or upregulation of amino acid transporters, which can undermine the durability of metabolic blockade. Achieving selective delivery to M2‐like TAMs remains complex due to their phenotypic and functional similarity to reparative macrophages in healthy tissues, increasing the risk of unintended immunomodulation. Furthermore, co‐delivery of ARG1 or ODC inhibitors alongside immunotherapeutic or redox‐active agents necessitates precise spatiotemporal control of drug release to maximize synergy while minimizing adverse effects. Addressing these limitations will require the development of rationally engineered nanocarriers with tunable release kinetics, improved TAM‐targeting specificity, and enhanced compatibility with combination treatment regimens to fully leverage the therapeutic potential of arginine metabolism modulation in cancer.

#### 3.2.4 Disrupting IDO1‐Kyn‐AhR Axis to Overcome Tumor Immune Evasion

The IDO1–Kyn–AhR axis represents a key immunoregulatory pathway across multiple tumor types.^[^ ^113^ ^]^ In this pathway, IDO1—and in some cancers, tryptophan 2,3‐dioxygenase (TDO2)—catalyzes the oxidative degradation of the essential amino acid tryptophan into Kyn. Accumulated Kyn acts as a high‐affinity ligand for AhR, a transcription factor expressed in various immune cell subsets. Activation of the Kyn–AhR pathway drives the differentiation of Tregs and tolerogenic myeloid cells, while suppressing the effector functions of CD8⁺ T cells and NK cells.^[^ ^113^ ^]^ Clinically, overexpression of IDO1 and hyperactivation of the Kyn–AhR axis are associated with immune evasion, resistance to PD‐1/PD‐L1 checkpoint blockade, and poor prognosis in cancers such as melanoma, lung, colorectal, and glioma. To counteract this immunosuppressive cascade, multiple therapeutic strategies have been pursued, including small‐molecule inhibitors, biologics, and genetic interventions.^[^ ^285^, ^314^ ^]^

Several small‐molecule IDO1 inhibitors have been designed to block tryptophan catabolism and reprogram the TME.^[^ ^285^, ^314^, ^315^ ^]^ Notable examples include: Epacadostat, Navoximod (GDC‐0919), and BMS‐986205 (Linrodostat). These agents showed encouraging immunostimulatory activity in preclinical models and early‐phase clinical trials. However, their efficacy in large phase III trials, especially as monotherapies, has been limited—likely due to pathway redundancy (e.g., TDO2 compensation), incomplete enzymatic blockade, and lack of patient stratification.^[^ ^316^ ^]^ An alternative approach involves tryptophan mimetics such as indoximod (1‐methyl‐D‐tryptophan),^[^ ^317^ ^]^ which does not directly inhibit IDO1 activity but instead reverses tryptophan depletion‐induced immunosuppression by restoring mTOR signaling in T cells and enhancing their effector functions.

Moreover, AhR antagonists are being developed to inhibit the downstream transcriptional effects of Kyn. Preclinical studies have demonstrated that AhR inhibition can reduce Treg differentiation, suppress M2 macrophage polarization, and delay tumor progression, especially in IDO1‐ or TDO2‐expressing tumors.^[^ ^113^ ^]^ Newer strategies under investigation include dual inhibitors targeting both IDO1 and TDO2 or combining AhR blockade with ICIs to achieve more durable immune activation and tumor regression.^[^ ^318^ ^]^ Biological interventions provide additional means of targeting the IDO1–Kyn–AhR axis:^[^ ^319^, ^320^ ^]^ Therapeutic vaccines directed against IDO1‐expressing cells have been shown to induce cytotoxic immune responses and yield favorable outcomes in early clinical studies. Enzyme therapies, such as kynureninase, act as metabolic sinks that deplete Kyn levels within the TME. In preclinical models, this approach enhances CD8⁺ T cell activity, reduces immunosuppressive myeloid populations, and leads to synergistic antitumor effects when combined with ICIs.^[^ ^321^ ^]^ Gene‐editing and silencing technologies provide strong mechanistic evidence for the therapeutic relevance of this axis: CRISPR‐Cas9‐mediated knockout of IDO1 in tumor cells or immune cells increases effector T cell infiltration and reduces Treg and myeloid‐derived suppressor cell (MDSC) populations.^[^ ^322^ ^]^ siRNA/shRNA‐mediated knockdown of IDO1 or AhR also enhances antitumor immunity in vivo.^[^ ^323^, ^324^ ^]^ While clinical translation of these approaches faces delivery and safety challenges, they offer invaluable insights and support the rationale for further drug development, such as proteolysis‐targeting chimeras (PROTACs) or metabolically resistant cell therapies.

Despite early clinical setbacks, particularly with IDO1 enzyme inhibitors like epacadostat,^[^ ^314^, ^316^ ^]^ current research focuses on overcoming redundancy and heterogeneity through biomarker‐guided patient selection and rational combination therapies, especially with ICIs. Nanotechnology offers powerful tools to enhance delivery and precision, addressing challenges such as poor solubility, rapid clearance, and off‐target toxicity.^[^ ^325^ ^]^ Nanoparticle systems also enable co‐delivery of synergistic agents—such as IDO1 inhibitors and AhR antagonists—within a single platform, maximizing efficacy while minimizing systemic side effects.^[^ ^213^, ^218^, ^326^ ^]^ For example, copper–cerium peroxide nanoparticles (CGDMRR) were developed to co‐deliver 1‐methyltryptophan, glucose oxidase, and doxorubicin, while alleviating tumor hypoxia. In 4T1 tumor models, CGDMRR suppressed tumor growth, enhanced antitumor immune responses, and, when combined with anti‐PD‐L1 antibody (aPD‐L1), improved systemic tumor control at both local and metastatic sites.^[^ ^327^ ^]^ Additionally, recent studies have explored nanomaterial‐based strategies for targeted degradation of IDO to alleviate tumor‐induced immunosuppression.^[^ ^219^, ^220^ ^]^ For example, a light‐activated PROTAC nanoassembly (LPN), composed of a PROTAC, a cathepsin B‐cleavable peptide linker, and a photosensitizer, was developed without additional carrier materials. Following intravenous administration, LPNs preferentially accumulated in tumor tissues via the EPR effect. Tumor‐associated cathepsin B cleaved the peptide linker, releasing the active PROTAC, while light irradiation triggered the photosensitizer to induce ICD and activate effector T cells. Concurrent IDO degradation suppressed tryptophan metabolism‐mediated Treg immunosuppression, collectively leading to effective inhibition of tumor growth, metastasis, and recurrence.^[^ ^219^ ^]^ In a separate study, researchers designed supramolecular artificial Nano‐AUTACs (GM NPs) by assembling an IDO‐targeting AUTAC molecule (GN) with the nucleoside analog methotrexate (MTX) via noncovalent interactions. These nanostructures enabled tumor‐specific delivery, where the acidic intracellular environment disrupted the supramolecular complex, releasing MTX to kill tumor cells, modulate tumor‐associated macrophages, activate dendritic cells, and induce autophagy. Autophagy in turn, enhanced GN‐mediated IDO degradation, boosting effector T cell responses and suppressing tumor progression.^[^ ^220^ ^]^

Although nanomaterial‐based strategies targeting the IDO1–Kyn–AhR axis hold significant therapeutic potential, several pathway‐specific challenges impede their clinical translation. First, the immunosuppressive effects mediated by this axis originate from multiple enzymes—including IDO1, TDO2, and IL4I1—raising the possibility of functional redundancy and necessitating broader or combinatorial inhibition strategies.^[^ ^328^ ^]^ Second, the heterogeneous expression of IDO1 and AhR across tumor types and immune cell subsets complicates patient stratification and may limit the efficacy of untargeted approaches. Third, the co‐delivery of multiple agents, such as IDO1 inhibitors and AhR antagonists, within a single nanoplatform requires precise spatial and temporal control of release kinetics and intracellular trafficking to ensure effective disruption of the pathway. Additionally, the immunosuppressive TME, which is characterized by high Kyn levels, hypoxia, and limited immune infiltration, poses a barrier to nanoparticle penetration and functional delivery. Finally, advanced nanoplatforms incorporating modalities such as PROTACs, enzyme therapies, or autophagy‐inducing constructs face challenges related to in vivo stability, immunogenicity, and potential off‐target effects, particularly given the physiological relevance of tryptophan metabolism in normal tissues. Addressing these mechanistic and delivery‐specific limitations will be essential to unlock the full potential of nanotechnology‐enabled modulation of the IDO1–Kyn–AhR axis in cancer immunotherapy.

### 3.3 Regulation of Lipid Metabolism Pathways in Tumors

Lipid metabolism plays a crucial role in tumor progression, survival, and therapy resistance.^[^ ^329^ ^]^ Tumor cells reprogram lipid pathways—upregulating lipid synthesis, altering FAO, and changing membrane composition—to support rapid proliferation and to reshape the TME.^[^ ^138^ ^]^ Consequently, targeting lipid metabolism has emerged as a promising therapeutic strategy in cancer treatment.^[^ ^330^ ^]^ A key enzyme in de novo lipid synthesis, FASN, is frequently overexpressed in cancers, including breast, prostate, and liver.^[^ ^134^ ^]^ Mechanistically, FASN supports membrane biosynthesis and energy storage under metabolic stress and contributes to immunosuppression. Recent in vivo CRISPR screens have identified FASN as a tumor‑intrinsic immunometabolic target.^[^ ^232^, ^331^, ^332^ ^]^ In a Braf/Pten‐driven melanoma model, FASN deletion impaired tumor growth in immunocompetent but not T cell‐deficient mice, indicating its specific role in immune evasion rather than proliferation. Tumors lacking FASN exhibited increased CD8⁺ T cell infiltration, enhanced IFN‐γ and TNF‐α production, and conversion from an immune‐cold to an immune‐hot phenotype. Corroboratively, bioinformatic analysis across multiple tumors revealed that high FASN expression inversely correlates with markers of cytolytic activity and immune infiltration, supporting its role in immune escape.^[^ ^331^ ^]^ Despite promising preclinical data, FASN inhibitors such as orlistat have yet to enter clinical trials for cancer due to limited specificity and toxicity.^[^ ^333^ ^]^ More potent drugs like TVB‑2640 (denifanstat) are under clinical investigation but show variable monotherapy efficacy, often limited by compensatory upregulation of FAO or exogenous lipid uptake.^[^ ^334^, ^335^, ^336^, ^337^ ^]^ Similarly, targeting FAO, such as with CPT1 inhibitors (e.g., etomoxir), has demonstrated preclinical tumor inhibition but faces clinical challenges due to off‐target toxicity.^[^ ^338^, ^339^, ^340^ ^]^ The convergence of CRISPR screening, mechanistic insight, and emerging pharmacology underscores lipid synthesis and FAO as high‐value immunometabolic axes in cancer. Disrupting these pathways, particularly when combined with immunotherapy, holds promise for reprogramming the TME and improving therapeutic efficacy.

Lipid droplets (LDs) are dynamic organelles that store neutral lipids and regulate metabolic homeostasis within tumor cells. They play a significant role in supporting tumor cell survival under metabolic stress, such as nutrient deprivation or hypoxia—common features of the TME.^[^ ^62^ ^]^ Recent studies suggest that targeting LD‐associated proteins or modulating lipid droplet metabolism could sensitize tumors to chemotherapy and radiation.^[^ ^140^, ^341^, ^342^ ^]^ Several small‐molecule inhibitors targeting LD formation and stabilization are currently in preclinical stages, although their clinical applications are still under development.^[^ ^343^, ^344^ ^]^ Cholesterol, a critical component of cellular membranes, particularly in lipid rafts, is involved in regulating signaling pathways that control cell proliferation, survival, and migration.^[^ ^32^ ^]^ HMG‐CoA reductase inhibitors (statins), which impede cholesterol biosynthesis via the mevalonate pathway, have been explored as potential adjuvants in cancer therapy.^[^ ^345^ ^]^ While statins have demonstrated modest anticancer effects in preclinical studies, their clinical efficacy remains a subject of ongoing investigation.^[^ ^346^, ^347^ ^]^ Recent research suggests that combining statins with ICIs may enhance antitumor immunity by modulating lipid metabolism and altering immune cell function within the TME.^[^ ^348^ ^]^ Tumor cells often manipulate lipid metabolism to evade immune surveillance and suppress immune responses.^[^ ^62^ ^]^ For example, lysophosphatidic acid (LPA), a bioactive lipid, has been shown to promote immune suppression by increasing the accumulation of MDSCs and Tregs in the TME. Inhibition of LPA receptors has been explored as a strategy to enhance antitumor immunity, with promising results in preclinical models.^[^ ^349^ ^]^ Additionally, enzymes such as phospholipase A2 (PLA2), which regulate the release of fatty acids from phospholipids, are being targeted to modulate lipid signaling pathways in cancer immunotherapy.^[^ ^350^ ^]^

Despite the promising preclinical data, inhibiting lipid metabolism in cancer therapy faces several challenges, including resistance mechanisms and the development of compensatory pathways. To enhance the efficacy of lipid metabolism inhibitors, combination therapies targeting multiple metabolic pathways and biomarker‐guided patient stratification are critical. Nanoparticle‐based delivery systems have transformed the targeting of lipid metabolism in cancer.^[^ ^351^ ^]^ By encapsulating agents such as FASN inhibitors, CPT1 inhibitors, and statins, these nanocarriers improve drug solubility and protect therapeutic payloads from degradation.

**Figure 6:**
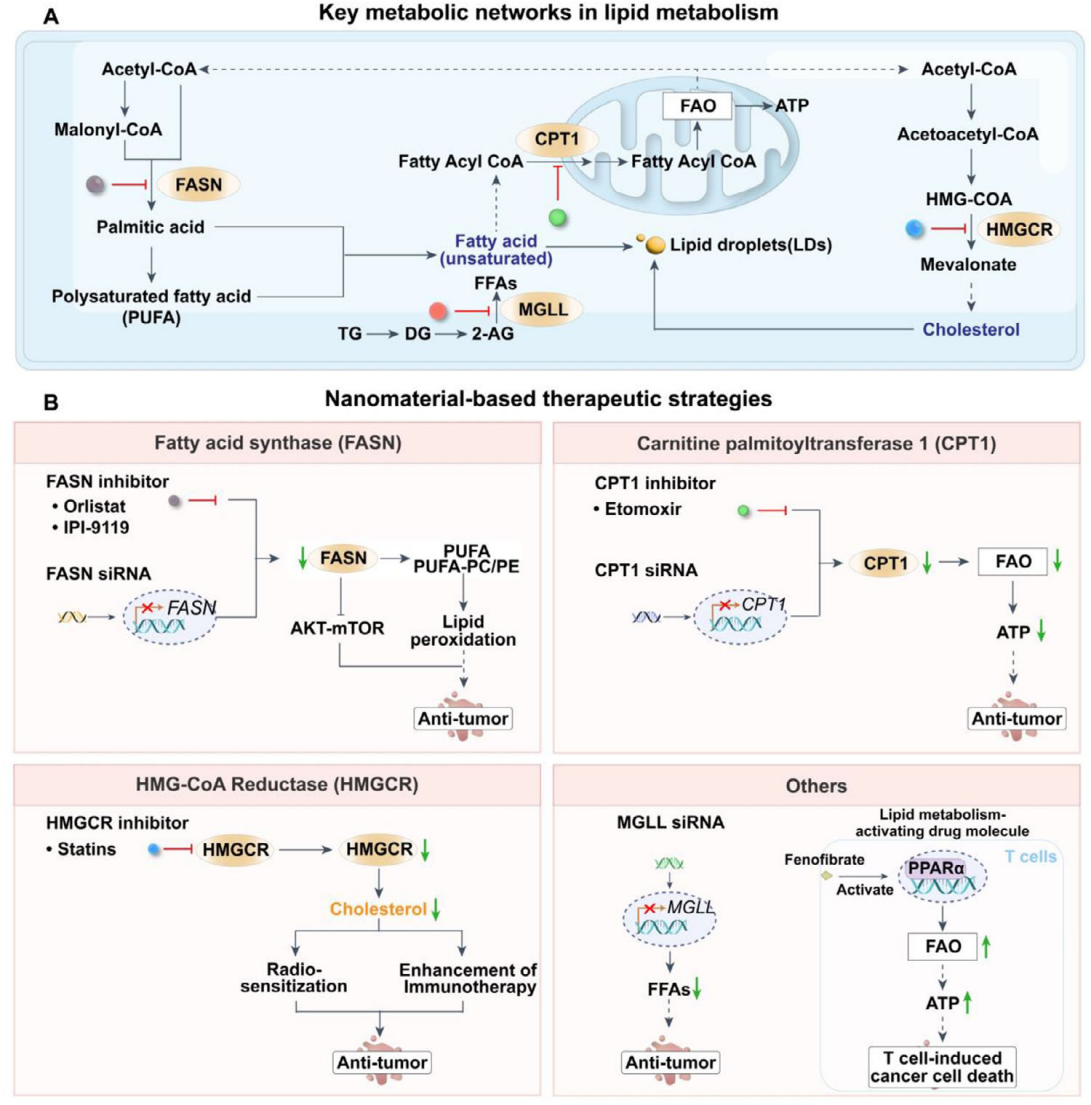
A) Schematic representation of key metabolic networks in lipid metabolism, illustrating major pathways and regulators, including FAO, FASN, carnitine palmitoyltransferase 1 (CPT1), lipid droplets (LDs), HMG‐CoA reductase (HMGCR), and monoacylglycerol lipase (MGLL). B) Nanomaterial‐based therapeutic strategies targeting these lipid metabolic pathways and components.

A growing body of preclinical research has focused on the development of nanoparticle‐based delivery systems designed to modulate aberrant lipid metabolism in tumors, aiming to enhance therapeutic efficacy and reduce systemic toxicity (**Table** **2**, **Figure** **6**).^[^ ^48^, ^53^, ^352^, ^353^, ^354^ ^]^ For example, a biomimetic nanovesicle, FiFe@RBM, was developed by cloaking superparamagnetic iron oxide nanoparticles (SPIONs) and a FASN inhibitor within red blood cell membranes. Following intravenous administration, FiFe@RBM selectively accumulated in PC‐3 castration‐resistant prostate cancer xenografts and enabled T_1_/T_2_‐weighted magnetic resonance imaging guidance. Within tumor cells, SPIONs released Fe^2^⁺/Fe^3^⁺ ions, catalyzing Fenton reactions to generate ROS, disrupt mitochondrial function, and inhibit the AKT–mTOR pathway. Simultaneously, FASN inhibition reprogrammed lipid metabolism, increasing levels of polyunsaturated phosphatidylcholine and phosphatidylethanolamine, thereby amplifying lipid peroxidation and promoting ferroptosis. The combined effects of magnetic hyperthermia and ferroptotic signaling induced both apoptotic and ferroptotic tumor cell death, suppressed primary tumor growth, and prevented liver metastasis, partly through recruitment and activation of NK cells. This dual‐modal nanoplatform integrates magnetic hyperthermia with lipid metabolism–driven ferroptosis, offering a precision therapeutic strategy for treatment‐resistant cancers.^[^ ^355^ ^]^ In another study, researchers developed two types of anti‐HER2–conjugated immunoliposomes (DSPC/Chol and DOPE/CHEMS) loaded with FASN‐targeting siRNA for breast cancer treatment. These nanosized formulations exhibited enhanced uptake and silencing efficiency in HER2⁺ SK‐BR3 cells, significantly reducing cell viability (to 30% and 20%) and impairing migration, while also downregulating FASN expression. Comparatively lower effects were observed in HER2^−^ MCF‐7 cells, demonstrating the selectivity and therapeutic potential of the targeted liposomes.^[^ ^356^ ^]^

**Table 2:** Representative Strategies for Modulating Key Components Involved in Lipid Metabolism, Nucleotide Metabolism, and Mitochondrial Function.

Metabolism pathway	Targets	Nano type	Nanomodulators	Key components	Treatment Modality	Tumor types	Ref.
Lipid metabolism	Fatty acid synthetase (FASN)	Polymeric nanoparticles	WO@V‐NPs	Orlistat	TT, MT	Breast cancer	[404]
			Tamoxifen/Orli nanocrystals	Orlistat	TT, MT	Solid ehrlich carcinoma	[405]
			ORL/HA‐CBZ/LPN	Orlistat	CT, TT	Prostate cancer	[406]
		Liposomal nanoparticles	LDLR‐OTN	Orlistat	TT	Breast cancer	[407]
			IL‐DSPC/Cholliposomes, IL‐DOPE/CHEMS liposomes	FASN siRNA	GT, MT	Breast Cancer	[356]
		Inorganic‐based nanostructures	FiFe@RBM	IPI‐9119	MHT, IO, MT	Castration‐resistant prostate cancer	[355]
	CPT1	Polymeric nanoparticles	2‐DG/aV‐siCPT1C NC	siCPT1C	GT, TT, MT	Glioblastoma	[358]
			VFETX	Etomoxir (ETX)	TT	Colorectal cancer, Breast cancer	[357]
	HMGCR	Polymeric nanoparticles	DOX‐M@CaP@ATV@HA	Atorvastatin (ATV)	TT	Breast cancer	[360]
			CPBA‐BSA (SOR + SIM) ‐NPs	Simvastatin (SIM)	TT	Breast cancer	[361]
			PLGA‐PEG‐MAN‐SIM	Simvastatin (SIM)	IO, TT	Hepatocellular carcinoma	[408]
			EMPP‐LOV	Lovastatin (LOV)	TT	Colorectal cancer	[409]
		Inorganic‐based nanostructures	ATV@FM@PMPC	Atorvastatin (ATV)	TT	Colon cancer	[359]
			Cu‐SF (RSV) NPs	Rosuvastatin (RSV)	TT, MT	Triple‐Negative Breast Cancer	[362]
	CD36 receptor	Polymeric nanoparticles	PPWQ NPs	Quercetin	IO, RT, MT	Breast cancer	[354]
			LHS NPs	Sulfosuccinimide oleate	CT, IO	Triple‐negative breast cancer	[410]
		Liposomal nanoparticles	CmEVs	Lipid‐lowering drug fenofibrate (FF)	RT	Glioblastoma	[411]
			LNP@E7&Mg	Magnesium ions	IO	Breast cancer	[412]
		Inorganic‐based nanostructures	iF‐CuS‐M/SSO@Gel	SSO	PTT, IO	Triple‐negative breast cancer	[413]
Lipid metabolism	Acyl‐CoA synthetase long‐chain family member (ACSL)	Polymeric nanoparticles	NPs (siACSL3)	siACSL3	GT, MT	Hepatocellular carcinoma	[414]
			OLCaP NP	Oleanolic acid (OA)	CT, NCT	Breast cancer	[415]
		Liposomal nanoparticles	siEIF3F‐LNPs	siEIF3F	GT, IO, MT	Hepatocellular carcinoma	[416]
			LNP‐miR‐211, D‐miR‐211, CNP‐miR‐211	miR‐211	GT, MT	Medulloblastoma	[417]
	Peroxisome proliferator‐activated receptor (PPAR)‐α, fatty acid metabolism‐related genes	Polymeric nanoparticles	aCD3/F/ANs	Fenofibrate	IMT	Melanoma	[353]
	Monoacylglycerol lipase (MGLL)	Polymeric nanoparticles	Reduction‐responsive RNAi NPs	MGLL siRNA	GT, IO, MT	Pancreatic cancer	[364]
Nucleotide metabolism	MTHFD2, γ‐aminobutyric acid (GABA)	Inorganic‐based nanostructures	EMφ‐siMTHFD2‐MnO2@Suni	siMTHFD2, MnO_2_	GT, IMT, CT	Renal Cell Carcinoma	[377]
	Inosine 5′‐monophosphate dehydrogenase (IMPDH)	Polymeric nanoparticles	D‐EMIPs	MIPs	MT, TT	Gastric cancer	[376]
	2′‐deoxyuridine 5′‐triphosphate nucleotidohydrolase (dUTPase)	Peptide‐based nanoparticles	RALA NPs	P4‐SedU2	CT, IO, MT	Colorectal cancer	[418]
	Poly ADP‐ribose polymerase (PARP)	Inorganic‐based nanostructures	CV‐Au NVs	Cisplatin, veliparib	CRT, TT	Lung cancer	[386]
	PARP, Homologous recombination (HR)	Liposomal nanoparticles	Veliparib/2HG‐coloaded liposomes	Veliparib, 2HG	IO, TT	Colon cancer	[419]
Nucleotide metabolism	Hypoxanthine nucleotide dehydrogenase	Polymeric nanoparticles	DMP‐NPs	6‐mercaptopurine (6‐MP)	TT, CT	Lymphoma	[420]
	CD73	Inorganic‐based nanostructures	ACS NPs	Au@Cu_2−x_Se NPs, DSF	CRT, IMT	Glioblastoma	[385]
			aPD‐L1/APCP@CaP	APCP	IMT, IO	Melanoma	[374]
Nucleotide metabolism	DHFR、TYMS and ATIC	Inorganic‐based nanostructures	FHM Nanoparticles	Methotrexate (MTX), histidine	TT, CT, MT	Colon cancer,Breast cancer	[421]
	Adenosine	Polymeric nanoparticles	HPNP	ADA	IO, IMT, SDT	Colon cancer,Breast cancer	[381]
	CD39, AMP‐activated protein kinase (AMPK)	Liposomal nanoparticles	C‐PMet	POM1, metformin	IMT, IO	Melanoma	[380]
	DHODH	Protein‐based nanoparticles	ATO/SRF@BSA	ATO	IMT, MT	Triple‐negative breast cancer	[375]
	A2A adenosine receptors	Polymeric nanoparticles	Pt‐PDA	Pt nanocatalyst, polydopamine	PTT, IO, MT	Triple negative breast cancer	[382]
			D/R@RPsP	SCH58261	IO, CT, MT	Breast cancer	[422]
			PPDAInNPs	SCH58261	PTT, IO, MT	Breast cancer	[423]
		Inorganic‐based nanostructures	Apt@SCH@BPs	SCH442416 (SCH)	PTT, IO, MT	Melanoma	[424]
			D/R/C@SiO_2_‐M	Catalase	CT, MT, IO	Colon cancer, Liver cancer	[425]
Regulation of mitochondrial function	Mitochondria	Liposomal nanoparticles	mtDSN	3‐(aminopropyl)triphenylphosphonium (TPP), fatty acids	IO, MT	Breast cancer, colorectal cancer	[426]
		Polymeric nanoparticles	LPT/HA‐CD NPs	TPP, Cisplatin prodrug (Pt(IV)), lonidamine (LND)	TT, CT, MT	Lung Cancer	[398]
		Protein‐based nanoparticles	SHC4H	Hydroxychloroquine, SMNB	PDT, TT, MT	Melanoma, Breast cancer, Pancreatic cancer, carcinoma	[402]
		Inorganic‐based nanostructures	CuET/ICG NPs	CuET	IO, PDT, MT	Triple‐negative breast cancer	[427]
Regulation of mitochondrial function	Mitochondria	Polymeric nanoparticles	OTA NPs	LND, oleanolic tertiary amine (OTA)	TT, MT	Glioblastoma	[428]
		Peptide‐based nanoparticles	KCKT	mitochondria targeting motif (KLAKLAK)_2_	SDT, TT, MT	Bladder cancer	[429]
		Polymeric nanoparticles	T‐Platin‐M‐NP,T‐Mito‐DCA‐NP.	TPP, Platin‐M, dichloroacetate (DCA)	CT, TT, MT	Breast cancer brain metastasis	[399]
		Inorganic‐based nanostructures	Cu‐Pic/HA NPs	Copper, Pic	IO, MT	Breast cancer	[430]
		Polymeric nanoparticles	TDC	TPP, Curcumin	TT	Hepatocellular cancer	[431]
		Polymeric nanoparticles	TPH/PTX nanomicelles	TPP, Paclitaxel (PTX)	CT, TT	Lung cancer	[403]
		Polymeric nanoparticles	pBAE‐mTOR NPs	Metallic ions (Ca^2+^,Zn^2+^, Li^+^,K^+^,Na^+^, Mg^2+^and Ba^2+^), mTOR siRNA	GT, MT, IO	Melanoma	[400]
		Polymeric nanoparticles	CS‐IR780/3BP@PLGA	IR780, 3‐Bromopyruvate (3BP)	SDT, CT, MT	Pancreatic cancer	[432]
		Inorganic‐based nanostructures	ICG@PM@NP	phenformin (PM)	PTT, MT, IO	Colorectal cancer	[397]
			D@HCC‐CuTH	Cu^2+^, DSF, calcium carbonate (CaCO_3_)	COMET	Breast cancer	[401]
			H‐MnCa/3MA‐ALD	3‐Methyladenine (3MA), H‐MnCa	CDT, TT, MT	Bone tumor	[433]
		Liposomal nanoparticles	αCD276‐Lip@AF	FdUMP, apigenin	CT, IO, MT	Colorectal cancer	[434]
			MiND NPs	Anti‐MFN2 peptide, tunicamycin, Bam7	COMET	Triple negative breast cancer	[435]

CDT: Chemo dynamic Therapy; COMET: Mitochondrial Endoplasmic Reticulum Therapy; CRT: Chemoradiotherapy; CT: Chemo Therapy; GT: Gene Therapy; IMT: Immunometabolic Therapy; IO: Immunotherapy; MHT: Magnetic Hyperthermia Therapy; MT: Metabolic Therapy; PDT: Photodynamic Therapy; PTT: Photothermal Therapy; RT: Radiation Therapy; SDT: Sonodynamic Therapy; TT: Targeted Therapy

Researchers have also developed nanomaterial‐based strategies to modulate CPT1 activity. For instance, a nutrient‐sensing nanodrug, VFETX—composed of vitamin B1 (VB1), ferrous ions, and etomoxir (ETX), an irreversible inhibitor of CPT1, the rate‐limiting enzyme in mitochondrial fatty acid β‐oxidation—was constructed. A fasting‐mimicking diet (FMD) upregulated VB1 transporter expression in tumor cells, thereby enhancing VFETX uptake and selective intratumoral accumulation. By inhibiting CPT1, ETX suppressed FAO and impaired mitochondrial energy production. The combined VFETX and FMD treatment synergistically disrupted tumor metabolic flexibility, resulting in significant tumor growth inhibition in vivo without notable systemic toxicity.^[^ ^357^ ^]^ Leveraging the elevated levels of ROS and GSH in the TME and cytoplasm, researchers developed a cascade‐responsive nanocapsule delivery system for glioblastoma (GBM) therapy. This system encapsulated a disulfide‐linked conjugate of anti‐VEGFR2 monoclonal antibody (aV) and CPT1C‐targeting siRNA (siCPT1C), and was surface‐loaded with the glycolysis inhibitor 2‐deoxy‐D‐glucose (2‐DG), forming the 2‐DG/aV‐siCPT1C nanocapsule (NC). Exploiting the overexpression of GLUT1 on both the blood–brain barrier (BBB) and GBM cells, the nanocapsule achieved effective BBB penetration and tumor targeting. By co‐delivering 2‐DG and siCPT1C, the system concurrently inhibited glycolysis and FAO in GBM, while also reducing angiogenesis. Additionally, the nanocapsule improved the stability and CNS delivery efficiency of both monoclonal antibodies and siRNA.^[^ ^358^ ^]^

Additionally, researchers have explored strategies to inhibit 3‐hydroxy‐3‐methylglutaryl‐CoA reductase (HMGCR)—the rate‐limiting enzyme of the mevalonate pathway—using small‐molecule inhibitors or siRNA to disrupt cholesterol biosynthesis and lipid‐dependent tumor growth.^[^ ^359^, ^360^, ^361^, ^362^, ^363^ ^]^ For instance, biodegradable zwitterionic polymer‐coated ferric metal–organic frameworks (Fe‐MOFs) co‐loaded with atorvastatin (ATV) were developed to induce ferroptosis. ATV inhibited HMGCR, impairing mevalonate and GSH biosynthesis, while Fe^2^⁺ released from disassembled MOFs catalyzed Fenton reactions to generate ROS, triggering lipid peroxidation and ferroptotic cell death. This synergistic strategy demonstrated potent antitumor efficacy and favorable biocompatibility in vitro and in vivo.^[^ ^359^ ^]^

Researchers developed T cell‐targeting nanoparticles (aCD3/F/ANs) encapsulating fenofibrate, a lipid metabolism‐modulating agent that activates peroxisome proliferator‐activated receptor‐α (PPAR‐α) and its downstream fatty acid metabolism‐related genes. In vitro, aCD3/F/ANs demonstrated superior T cell uptake compared to non‐targeted formulations (F/ANs). Treated T cells exhibited well‐defined mitochondrial cristae, increased ΔΨm, and elevated oxygen consumption rates under glucose‐deprived conditions, mimicking the TME. Gene expression analyses confirmed greater upregulation of PPAR‐α and related metabolic genes in aCD3/F/AN‐treated T cells. This metabolic reprogramming enhanced T cell proliferation and survival under nutrient‐limited conditions. Live‐cell imaging revealed increased cytotoxic activity of treated T cells against B16F10 melanoma cells. In vivo, systemic administration of aCD3/F/ANs promoted T cell infiltration into tumors, elevated intratumoral cytokine production, and significantly inhibited tumor growth in murine models. These findings underscore the potential of nanoparticle‐enabled lipid metabolic reprogramming to boost T cell function in immunometabolic cancer therapy.^[^ ^353^ ^]^ Additionally, researchers demonstrated that monoacylglycerol lipase (MGLL), a key lipid‐catabolizing enzyme, and cannabinoid receptor 2 (CB2), a regulator of macrophage polarization, were overexpressed in pancreatic adenocarcinoma (PAC) cells and TAMs, respectively. To simultaneously target these components, they engineered a reduction‐responsive poly(disulfide amide) (PDSA) nanoplatform for the co‐delivery of siRNAs against MGLL (siMGLL) and CB2 (siCB2). In the reductive TME, cleavage of PDSA's disulfide bonds triggered siRNA release. MGLL silencing reduced free fatty acid (FFA) production in PAC cells, limiting lipid availability and metabolic support, while CB2 knockdown reprogrammed TAMs toward an M1‐like proinflammatory phenotype, enhancing TNF‐α and IL‐12 secretion. This dual targeting of tumor metabolism and the immune microenvironment synergistically suppressed tumor growth in both subcutaneous and orthotopic PAC mouse models.^[^ ^364^ ^]^

These strategies underscore the potential of integrating lipid‐targeted therapies with nanotechnology to overcome metabolic plasticity and enhance both direct tumor cell killing and immune‐mediated clearance. However, nanomaterial‐based approaches targeting lipid metabolism, including FASN, CPT1, HMGCR, and lipid signaling mediators, face several lipid‐specific challenges that hinder clinical translation. First, key lipid metabolic enzymes are not tumor‐specific and are critical for maintaining lipid homeostasis in normal tissues, raising the risk of on‐target, off‐tumor toxicity. Second, tumors exhibit considerable metabolic flexibility; upon inhibition of a single lipid pathway, they may upregulate compensatory mechanisms such as exogenous lipid uptake, enhanced FAO, or mevalonate‐independent cholesterol synthesis, which can diminish therapeutic efficacy. Third, intertumoral and intratumoral heterogeneity in lipid metabolism—both in cancer cells and immune cell subsets—complicates treatment uniformity and underscores the need for biomarker‐driven patient selection. Moreover, lipid‐rich and often hypoxic TME present physical and biochemical barriers to nanoparticle penetration and release, complicating the coordinated delivery of multiple agents, including siRNAs and small‐molecule inhibitors. Finally, immune reprogramming strategies that target lipid metabolism in TILs or tumor‐associated macrophages require highly selective delivery systems to avoid suppressing beneficial immune responses. Addressing these challenges will necessitate the rational design of multifunctional, cell‐specific nanocarriers, integration of combinatorial metabolic interventions, and deeper mechanistic understanding of lipid metabolic interactions within the tumor‐immune ecosystem.

### 3.4 Regulation of Nucleotide Metabolism Pathways in Tumors

Nucleotide metabolism is essential for DNA and RNA synthesis, cell cycle progression, and epigenetic regulation. In cancer, the elevated demand for nucleotides to support rapid proliferation and preserve genomic stability drives extensive reprogramming of both de novo and salvage pathways. This metabolic adaptation not only facilitates tumor growth but also contributes to therapeutic resistance, immune evasion, and altered responses to genotoxic stress.^[^ ^31^, ^153^, ^168^ ^]^

Among key targets in nucleotide metabolism, dihydroorotate dehydrogenase (DHODH)—the rate‐limiting enzyme in de novo pyrimidine biosynthesis—has garnered significant attention.^[^ ^153^ ^]^ Inhibitors such as leflunomide and BAY2402234 have demonstrated potent antitumor activity in preclinical models.^[^ ^365^ ^]^ In addition, TYMS and ribonucleotide reductase (RNR), which are essential for the synthesis of deoxythymidine monophosphate (dTMP) and deoxyribonucleotide triphosphates (dNTPs), remain important therapeutic targets.^[^ ^366^, ^367^ ^]^ Conventional agents such as 5‐fluorouracil (5‐FU) and hydroxyurea are widely used,^[^ ^368^, ^369^ ^]^ and newer‐generation inhibitors with greater selectivity and reduced toxicity are under investigation. In the purine biosynthetic pathway, inhibitors like mycophenolate mofetil (MMF) and 6‐mercaptopurine continue to be evaluated in tumors with elevated purine demand.^[^ ^370^, ^371^ ^]^ Notably, dual targeting of purine biosynthesis and immune checkpoints has shown synergistic effects in immunologically “cold” tumors, enhancing the efficacy of ICB. Nucleotide metabolism also plays a critical role in modulating the tumor immune microenvironment. Although extracellular ATP functions as a pro‐inflammatory signal promoting immune activation, its degradation to adenosine by CD39/CD73 ectonucleotidases exerts potent immunosuppressive effects.^[^ ^372^ ^]^ Consequently, inhibitors of adenosine‐generating enzymes or adenosine A2A receptor antagonists are being actively investigated in clinical trials to restore antitumor immune responses and augment immunotherapy.

**Figure 7:**
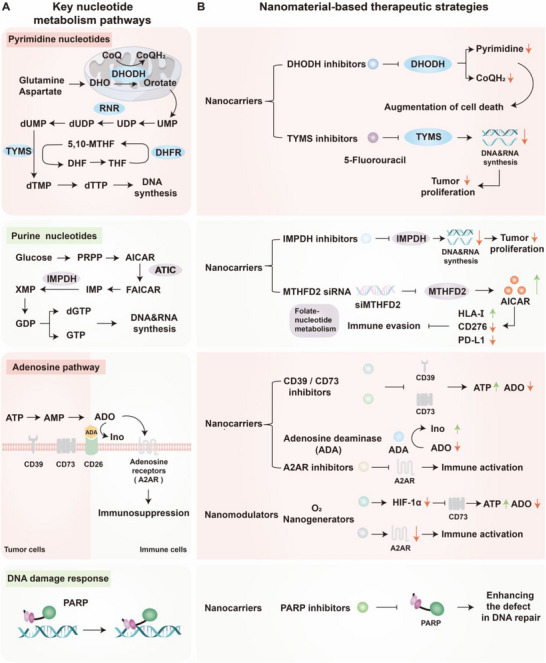
A) Schematic representation of key nucleotide metabolic pathways, highlighting major enzymes and regulatory nodes, including DHODH, TYMS, IMPDH, folate‐nucleotide metabolism, CD39, CD73, A2AR, and PARP. B) Nanomaterial‐based therapeutic strategies are designed to target these nucleotide metabolic components for inhibiting tumor proliferation, modulating the tumor microenvironment, and enhancing anticancer efficacy.

To overcome challenges associated with poor pharmacokinetics and systemic toxicity of nucleotide metabolism inhibitors, nanoparticle‐based delivery systems have been developed (Table 2, **Figure** **7**).^[^ ^373^, ^374^ ^]^ These nanocarriers enhance the bioavailability and tumor specificity of agents such as DHODH and TYMS inhibitors, while also enabling co‐delivery with DNA‐damaging chemotherapeutics or immune modulators.^[^ ^369^, ^375^ ^]^ Preclinical studies have demonstrated that such platforms can improve therapeutic efficacy and reduce off‐target effects. Researchers synthesized dual‐template epitope‐imprinted polymers (D‐EMIPs) via a surface‐imprinting technique, using a HER2‐derived peptide (GSRCWGES) and an IMPDH active‐site peptide (YRGMGSLD) as templates. The resulting nanoparticles selectively bound to HER2 on cancer cell membranes, significantly enhancing their uptake into HER2⁺ cells. Once internalized, D‐EMIPs engaged and inhibited inosine‐5’‐monophosphate dehydrogenase (IMPDH), thereby suppressing guanine nucleotide synthesis, impairing DNA replication, and arresting cell proliferation. In vitro and in vivo studies showed that D‐EMIPs preferentially accumulated in HER2⁺ tumors and markedly suppressed tumor growth with minimal off‐target toxicity. This study demonstrated that dual‐epitope imprinting enabled the integration of active targeting and enzyme inhibition within a single polymeric platform, offering a precise strategy to disrupt nucleotide metabolism in cancer cells.^[^ ^376^ ^]^

In another study, researchers have employed siRNA to modulate folate‐nucleotide metabolism as a strategy to disrupt purine biosynthesis. In sunitinib‐resistant clear cell renal cell carcinoma (ccRCC), CD276—an immune checkpoint overexpressed on cancer stem cells—and MTHFD2—a mitochondrial enzyme in the folate cycle—were identified as promising therapeutic targets. To exploit these vulnerabilities, a multifunctional nanotheranostic platform (EMφ‐siMTHFD2‐MnO_2_@Suni) was developed using CD276‐CD133 single‐chain variable fragment (ScFv)‐modified M1 macrophage‐derived nanovesicles for dual targeting and deep tumor penetration. This biomimetic system co‐delivered siRNA against MTHFD2 and MnO_2_‐loaded sunitinib, with TME‐responsive release. MTHFD2 knockdown disrupted folate and purine metabolism, while MnO_2_ modulated GABA metabolism, collectively contributing to epithelial–mesenchymal transition inhibition and reprogramming of the immunosuppressive TME. In vivo, the platform significantly inhibited tumor growth, metastasis, and recurrence, underscoring its potential for overcoming drug resistance through metabolic and immune modulation.^[^ ^377^ ^]^

Many studies have explored nanomaterial‐based strategies to target adenosine‐generating enzymes, the adenosine A2A receptor, or adenosine itself, aiming to reverse immunosuppression and restore antitumor immune responses.^[^ ^378^, ^379^, ^380^, ^381^, ^382^, ^383^, ^384^ ^]^ For instance, calcium phosphate (CaP) nanoparticles were engineered via a biomineralization strategy to co‐deliver aPD‐L1 and APCP, a small‐molecule CD73 inhibitor. These nanocarriers exhibited high dual‐drug loading and selectively released both agents under acidic conditions. In a murine melanoma model, CaP‐mediated delivery of aPD‐L1 at one‐twentieth the systemic dose of the free antibody achieved superior tumor control while avoiding the off‐target toxicities associated with high‐dose administration. Co‐delivery of APCP further reduced extracellular adenosine, enhanced CD8⁺ T cell infiltration, and induced durable immune memory that prevented tumor recurrence. Collectively, these findings demonstrated that pH‐responsive CaP nanoparticles enabled synergistic blockade of PD‐L1 and CD73, significantly enhancing the efficacy and safety of melanoma immunotherapy.^[^ ^374^ ^]^ In a separate study, investigators leveraged the intrinsic physicochemical properties of bare nanoparticles to downregulate CD73 expression on tumor cells, thereby alleviating adenosine‐mediated immunosuppression without the need for exogenous ectonucleotidase inhibitors. They engineered Au@Cu_2−x_Se nanoparticles (ACS NPs), which accumulated in GBM tumors and generated hydroxyl radicals via a Fenton‐like reaction, relieving hypoxia and downregulating CD73 (ecto‐5’‐nucleotidase) expression. This reduction in CD73 decreased extracellular adenosine levels and mitigated adenosine‐induced immunosuppression. Simultaneously, released Cu^2^⁺ ions chelated intracellular disulfides to form the cytotoxic bis(N,N‐diethyldithiocarbamate)–copper (CuET) complex, which synergized with radiotherapy to amplify antitumor immune responses. In treated mice, this combination significantly increased intratumoral infiltration of CD8⁺ and CD4⁺ T cells and expanded splenic memory T cells, ultimately suppressing GBM growth by 92%. This study demonstrated that nanoparticle‐mediated CD73 inhibition, combined with chemoradiotherapy, offered an effective strategy to overcome adenosine‐driven immune evasion and enhance GBM immunotherapy.^[^ ^385^ ^]^ Additionally, a nanomaterial‐based catalytic immunocomplex was designed to modulate the adenosine pathway through ultrasound‐triggered activation within the acidic TME. This multifunctional nanoplatform co‐delivered adenosine deaminase (ADA), aPD‐L1, and a sonosensitizer (hematoporphyrin), all linked via acid‐cleavable and singlet oxygen–responsive linkers. Upon ultrasound stimulation, the complex simultaneously induced sonodynamic therapy, immune checkpoint blockade, and enzymatic degradation of intratumoral adenosine by ADA. This reduced A2A/A2B receptor signaling, thereby relieving immunosuppression and enhancing effector T cell activation. This strategy exemplifies a programmable and TME‐responsive nanotherapeutic approach to effectively target adenosine‐mediated immune evasion in cancer.^[^ ^381^ ^]^

Additionally, researchers have investigated the use of small‐molecule inhibitors to block poly(ADP‐ribose) polymerase (PARP)‐dependent DNA repair pathways.^[^ ^386^ ^]^ A gold nanoparticle‐based, radiation‐responsive nanovesicle system (CV‐AuNVs), co‐loaded with cisplatin and the PARP inhibitor veliparib, was developed to enhance chemoradiation efficacy in a spatiotemporally controlled manner. Upon X‐ray irradiation, hydroxyl radicals (•OH) generated in situ oxidized the polyphenylene sulfide shell, transforming it from hydrophobic to hydrophilic and triggering vesicle disassembly and drug release. The combination of cisplatin‐induced DNA damage and veliparib‐mediated inhibition of DNA repair synergistically induced tumor cell apoptosis. In vivo studies using an A549 lung cancer model demonstrated that a single administration of CV‐AuNVs with one‐time irradiation markedly suppressed tumor growth.

Although nanotechnology‐enabled strategies targeting nucleotide metabolism hold considerable promise for cancer therapy, several pathway‐specific barriers hinder their clinical translation. A major concern is the high basal activity of enzymes such as DHODH, TYMS, and IMPDH in normal proliferative tissues, including the hematopoietic and gastrointestinal compartments. This widespread expression raises the risk of on‐target toxicity, even with tumor‐directed delivery. Complicating therapeutic precision is the intratumoral and intertumoral heterogeneity in nucleotide demand and pathway activity, which varies with tumor subtype, mutational context, and treatment stage. This necessitates dynamic biomarker development and adaptable therapeutic schedules to identify patients most likely to benefit. From a delivery standpoint, synchronizing the release of nucleotide metabolism modulators—such as siRNAs, nucleoside analogs, and immune checkpoint inhibitors—within nanocarriers remains technically challenging, particularly in the presence of physical barriers like dense stroma and elevated interstitial fluid pressure. These features restrict nanoparticle penetration into immunosuppressive tumor regions enriched in adenosine and CD73. Furthermore, effective targeting of the extracellular nucleotide axis (e.g., CD39/CD73–A2AR signaling) requires delivery platforms that maintain structural and functional stability in acidic and hypoxic tumor microenvironments. Finally, emerging modalities—such as epitope‐imprinted polymers and catalytic immunocomplexes—must be rigorously optimized to prevent off‐target immune activation and ensure long‐term biocompatibility. Addressing these challenges will require context‐sensitive nanocarrier design, real‐time metabolic profiling, and combinatorial regimens tailored to the immunometabolic landscape of individual tumors.

### 3.5 Regulation of Mitochondrial Function and Associated Pathways in Tumors

Mitochondria play a critical role in cellular energy production, homeostasis, and metabolic regulation, all of which are essential for tumor progression. Advances in cancer biology have revealed how mitochondrial dysfunction and metabolic reprogramming contribute to the hallmarks of cancer. Tumor cells often undergo adaptive changes in mitochondrial function to support rapid cell proliferation, survival under hypoxic conditions, and resistance to therapies.^[^ ^35^, ^160^, ^162^, ^387^, ^388^ ^]^

Altered mitochondrial dynamics, including changes in mitochondrial biogenesis, oxidative phosphorylation, and the mitochondrial ETC, are commonly observed in cancer cells.^[^ ^160^, ^161^, ^388^ ^]^ These changes contribute to enhanced ATP production and cellular biosynthesis, supporting tumor growth. Mitochondrial biogenesis in tumor cells is regulated by key factors such as PPARγ coactivator 1α (PGC‐1α) and AMP‐activated protein kinase (AMPK), which help promote mitochondrial function and cell survival under stress.^[^ ^389^ ^]^ Disrupted mitochondrial dynamics, such as imbalances in mitochondrial fusion and fission, are also implicated in metastasis.^[^ ^390^ ^]^ Proteins like mitofusins, which regulate mitochondrial fusion, and Drp1, which regulates mitochondrial fission, play critical roles in maintaining mitochondrial adaptability and bioenergetics in tumor cells.^[^ ^387^, ^391^ ^]^

Despite the prominence of aerobic glycolysis, mitochondria remain crucial for ATP production, particularly in more aggressive tumors or during nutrient deprivation. Key components of the ETC, particularly Complexes I and II, are often upregulated to support oxidative phosphorylation and meet the energy demands of tumor cells. Targeting oxidative phosphorylation regulators, such as ATP synthase or Complex I inhibitors, is emerging as a strategy to induce metabolic stress and sensitize tumors to conventional therapies, including chemotherapy.^[^ ^168^, ^387^ ^]^ Increased mitochondrial ROS production is a natural byproduct of enhanced oxidative phosphorylation in tumor cells. While low ROS levels can promote tumorigenesis by facilitating signaling and genome instability,^[^ ^392^, ^393^ ^]^ excessive ROS can lead to mitochondrial dysfunction and cell death. Tumors with elevated ROS levels have developed antioxidant defense mechanisms involving enzymes like superoxide dismutases (SODs) and glutathione peroxidases, which mitigate oxidative stress and promote cell survival.

Mitochondrial‐targeted therapies are being explored to selectively inhibit mitochondrial function in cancer cells.^[^ ^168^, ^387^ ^]^ For example, mitochondrial kinase inhibitors such as metformin are being studied for their potential to disrupt mitochondrial metabolism. Additionally, compounds targeting the ΔΨm are under investigation, as altering ΔΨm could induce apoptosis or autophagic cell death in tumor cells. Inhibitors of mitochondrial fusion, such as those targeting mitofusins, and Drp1 inhibitors are also in preclinical development, with the aim of selectively impairing tumor cell metabolism and survival.

Preclinical studies have shown that combining mitochondrial inhibitors with conventional therapies can overcome resistance mechanisms that arise from enhanced glycolysis or oxidative phosphorylation.^[^ ^387^ ^]^ Metformin and bortezomib, agents known to influence mitochondrial proteostasis, have been investigated for their potential to sensitize tumors to chemotherapy and immunotherapy. Clinical trials, such as the phase I study combining metformin, nelfinavir, and bortezomib in patients with relapsed and refractory multiple myeloma, are exploring the efficacy of these combinations (NCI‐2019‐00466).^[^ ^394^ ^]^ However, preclinical studies have shown that metformin may induce resistance to bortezomib by impairing the activity and assembly of the 26S proteasome complex,^[^ ^395^ ^]^ highlighting the complexity of their interaction and the need for further research. However, challenges remain, including off‐target toxicity, poor drug delivery efficiency, and tumor heterogeneity, which must be addressed to enable successful clinical translation of mitochondrial‐targeted therapies.

**Figure 8:**
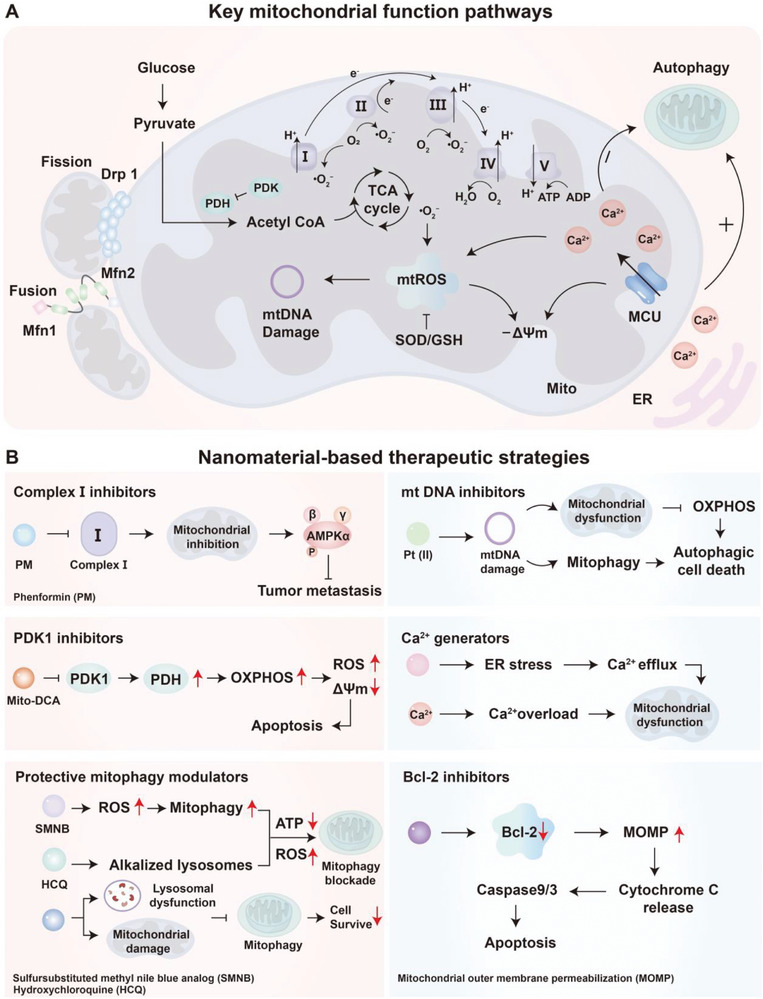
A) Schematic illustration of key mitochondrial functional pathways involved in energy metabolism, redox balance, apoptosis regulation, and biosynthetic processes. B) Nanomaterial‐based therapeutic strategies are designed to target mitochondrial pathways, aiming to impair tumor cell proliferation, induce apoptosis, and enhance overall antitumor efficacy.

Nanoparticles are powerful tools for targeting mitochondrial function in tumor cells. A variety of multifunctional nanoparticles have been developed to optimize mitochondrial‐targeted therapies, offering new strategies for cancer treatment (**Figure** **8**, Table 2).^[^ ^396^ ^]^ A hydrogen peroxide‐responsive manganese dioxide (MnO_2_)‐mineralized albumin nanocomposite was synthesized using a modified two‐step biomineralization method to co‐encapsulate phenformin (PM), a mitochondria‐targeting inhibitor, and indocyanine green (ICG), a near‐infrared photothermal dye. Upon activation in the TME, ICG‐mediated mild PTT and PM‐induced mitochondrial dysfunction synergistically downregulated PD‐L1 expression. This combination therapy elicited robust antitumor immune responses capable of eliminating residual tumor cells. Additionally, the treatment significantly suppressed tumor metastasis by inhibiting invasion‐associated signaling molecules, including TGF‐β and vimentin.^[^ ^397^ ^]^ A self‐assembled nano‐targeted drug delivery system, LND‐SS‐Pt‐TPP/HA‐CD, was constructed using β‐cyclodextrin‐grafted hyaluronic acid (HA‐CD) to encapsulate prodrug nanoparticles for targeted tumor delivery via the CD44 receptor. The system incorporated a triphenylphosphine (TPP⁺) moiety to facilitate mitochondrial localization. Disulfide bonds within the nanoparticles were selectively cleaved by mitochondrial GSH, triggering the release of lonidamine (LND) and a cisplatin prodrug (Pt(IV)). Under mitochondrial reducing conditions, Pt(IV) was further reduced to active cisplatin (Pt(II)) by GSH and ascorbic acid, inducing mitochondrial DNA damage, dysfunction, and mitophagy, thereby disrupting oxidative phosphorylation. Simultaneously, LND inhibited HK2, contributing to mitochondrial impairment and glycolysis suppression, ultimately depriving cancer cells of energy. This dual‐action delivery system effectively overcame cisplatin resistance in lung cancer cells and demonstrated promise for mitochondrial‐targeted cancer therapy.^[^ ^398^ ^]^

To address metabolic plasticity in brain metastases, researchers developed mitochondria‐targeted, blood–brain barrier–penetrating nanoparticles co‐delivering a chemotherapeutic prodrug and a pyruvate dehydrogenase kinase 1 inhibitor. This dual inhibition of oxidative phosphorylation and glycolysis impaired tumor bioenergetics and improved therapeutic efficacy, with in vivo studies confirming safety and reduced chemoresistance through mitochondrial genome targeting.^[^ ^399^ ^]^ Some studies have disrupted mitochondrial Ca^2^⁺ homeostasis in cancer cells to induce mitochondrial dysfunction by triggering a self‐amplifying loop of Ca^2^⁺ overload and ROS accumulation.^[^ ^400^, ^401^ ^]^ Researchers also explored nanomaterial‐based strategies to inhibit mitophagy.^[^ ^402^ ^]^ For instance, they developed a hypoxia‐responsive supramolecular albumin nanoparticle modified with azocalix[4]arene to co‐deliver hydroxychloroquine and a mitochondria‐targeting photosensitizer for the treatment of hypoxic tumors. Under hypoxic conditions, the nanoparticles selectively released their payload within tumor cells. The released hydroxychloroquine inhibited mitophagy, thereby increasing oxidative stress and sensitizing tumor cells. Upon laser irradiation, the photosensitizer generated oxygen‐independent ROS, inducing mitochondrial damage and further activating mitophagy. The combination of mitophagy flux blockade and ROS‐mediated mitochondrial stress led to amplified autophagic disruption and oxidative damage, ultimately enhancing tumor cell death. This approach demonstrated the therapeutic potential of integrating mitophagy inhibition with mitochondria‐targeted PDT in hypoxic tumor environments.^[^ ^402^ ^]^ Additionally, researchers employed nanomaterial‐based strategies to induce mitochondrial outer membrane permeabilization (MOMP) by inhibiting the antiapoptotic protein Bcl‐2, which facilitated cytochrome c release and triggered caspase‐9 and caspase‐3 activation, ultimately leading to apoptosis of tumor cells.^[^ ^403^ ^]^

Despite notable progress, nanomaterial‐based strategies targeting mitochondrial function in tumors face several pathway‐specific challenges that limit clinical translation. A primary concern lies in the lack of tumor specificity for many mitochondrial targets—such as components of the ETC, ΔΨm, and regulators of mitochondrial dynamics including Drp1 and mitofusins—which are also critical for normal cells, particularly in high‐energy‐demand tissues like the heart and brain. This overlap raises the risk of on‐target, off‐tumor toxicity. The compartmentalized and dynamic nature of mitochondrial processes also presents substantial delivery obstacles. Effective targeting requires specialized nanocarriers equipped with organelle‐localizing moieties (e.g., TPP⁺) and environment‐responsive linkers. However, these systems often suffer from limited in vivo stability, inefficient intracellular trafficking, or inadequate tissue penetration. Moreover, mitochondrial plasticity allows tumor cells to adapt to single‐pathway inhibition by activating compensatory mechanisms such as glycolysis, mitochondrial biogenesis, or enhanced antioxidant defenses, thereby reducing therapeutic durability. Efforts to co‐target multiple mitochondrial pathways—such as oxidative phosphorylation, mitophagy, and calcium homeostasis—within a single nanoplatform demand precise control over drug release kinetics to avoid systemic toxicity and unintended immune suppression. The TME further complicates delivery, as hypoxia, elevated interstitial pressure, and dense stroma impede nanoparticle infiltration into mitochondria‐rich tumor regions. Additionally, mitochondrial heterogeneity among tumor subtypes and within cancer stem cell populations necessitates biomarker‐guided patient stratification to improve treatment precision and efficacy. Overcoming these limitations will require the rational design of mitochondria‐targeting nanocarriers, incorporation of feedback‐responsive delivery systems, and development of context‐specific combination therapies tailored to the mitochondrial and immunometabolic landscape of each tumor.

## 4 Conclusions and Outlook

Metabolic reprogramming is a hallmark of cancer, driving tumor progression, immune evasion, and resistance to therapy.^[^ ^8^ ^]^ Tumor cells rewire core metabolic pathways—including glucose, amino acid, lipid, nucleotide, and mitochondrial metabolism—to support rapid proliferation, survive under stress, and resist conventional treatments.^[^ ^436^ ^]^ These metabolic adaptations critically shape the TME, fostering immunosuppression and therapeutic failure. Among these pathways, amino acid metabolism—particularly the tryptophan–Kyn–AhR axis (often driven by IDO1 activity) and glutamine metabolism—is increasingly recognized as a high‐priority target due to its central role in T cell dysfunction and immune suppression within the TME. Glucose metabolism, notably aerobic glycolysis and lactate accumulation, is also critically involved, contributing to CD8⁺ T cell exhaustion and reinforcing an immunosuppressive milieu. Other metabolic pathways, such as those involving lipid, nucleotide, and mitochondrial function, are emerging as additional contributors to tumor immune escape, with growing evidence supporting their relevance in preclinical models and potential therapeutic exploitation.

Targeting key mediators such as GLUTs, LDH, and MCTs represents a promising therapeutic strategy. Similarly, disrupting amino acid metabolism—via GLS, amino acid transporters, and arginine‐metabolizing enzymes—can help restore antitumor immunity. The IDO1–kyn–AhR axis, a central mediator of immune suppression, is under active investigation for its potential to enhance immunotherapy efficacy. Lipid metabolism, including fatty acid synthesis and oxidation as well as cholesterol biosynthesis, supports tumor growth and modulates immune responses.^[^ ^437^ ^]^ Inhibiting these pathways, particularly in combination with ICIs, has shown promising results in preclinical models. Additionally, nucleotide metabolism and mitochondrial function are essential for DNA repair, redox homeostasis, and energy production, making them attractive therapeutic targets.

The emerging field of immunometabolism underscores the bidirectional relationship between tumor metabolic activity and immune cell function.^[^ ^8^ ^]^ Metabolic alterations within tumor cells can either suppress or stimulate immune responses depending on the context. Preclinical evidence suggests that targeting tumor metabolism can enhance antitumor immunity, especially when combined with ICIs. However, clinical translation remains challenging due to issues such as metabolic redundancy, intertumoral and intratumoral heterogeneity, limited tumor specificity, and the potential for off‐target toxicity. Nanotechnology‐based delivery systems offer powerful solutions to address these limitations. By improving the solubility, pharmacokinetics, and tumor specificity of metabolic inhibitors, nanoparticles can enhance therapeutic efficacy while minimizing systemic toxicity.^[^ ^438^ ^]^ Advanced platforms such as stimuli‐responsive nanoparticles—including pH‐sensitive and redox‐responsive systems—facilitate site‐specific drug release within the TME. Ligand‐ and receptor‐targeted nanocarriers further enhance selective uptake by tumor or immune cells. Multifunctional nanoplatforms, such as those co‐delivering IDO1 inhibitors alongside ICIs, have demonstrated synergistic immunometabolic reprogramming in preclinical models. In addition, biomimetic delivery systems, including cell membrane‐coated nanoparticles, offer improved circulation time, tumor targeting, and immune evasion. Despite these advances, challenges remain, including suboptimal delivery efficiency, heterogeneous metabolic phenotypes across tumors and patients, and an incomplete understanding of compensatory metabolic adaptations.^[^ ^439^ ^]^ Continued efforts toward the rational design of nanocarriers, integration of metabolic biomarkers for patient stratification, and development of combination regimens will be essential to fully realize the therapeutic potential of targeting tumor immunometabolism.

To advance the clinical translation of immunometabolic therapies, several priority research directions should be pursued:

*Development of metabolic biomarkers for patient stratification*: Integrating metabolomics, advanced imaging, and circulating metabolic markers into clinical trials will enable real‐time assessment of tumor and immune cell metabolism. This approach will support adaptive treatment strategies and improve the selection of patients most likely to benefit from metabolic interventions.

*Design of personalized nanotherapies*: Rationally engineered nanocarriers incorporating tumor‐specific ligands, metabolic biomarkers, and microenvironment‐responsive elements (e.g., pH, redox, enzymatic triggers) can enhance the precision and selectivity of metabolic inhibitor delivery, minimizing off‐target effects and improving therapeutic outcomes.

*Implementation of translationally relevant preclinical models*: Humanized mouse models and patient‐derived organoids co‐cultured with immune cells are essential for accurately recapitulating tumor‐immune metabolic interactions and evaluating the immunomodulatory effects of metabolic nanotherapies under physiologically relevant conditions.

*Investigation of host metabolic influences*: Systematic studies examining how host‐related factors—such as obesity, insulin resistance, or gut microbiota—affect tumor metabolism and immune responses will inform personalized therapeutic strategies and may uncover new combinatorial targets.

*Overcoming metabolic plasticity and compensation*: Elucidating how tumors adapt to metabolic stress by reprogramming between glycolysis, oxidative phosphorylation, amino acid utilization, and lipid metabolism is crucial for designing effective multi‐targeted or sequential regimens that minimize therapeutic escape.

*Development of organelle‐specific delivery platforms*: Mitochondria‐ or lysosome‐targeted nanocarriers offer a promising avenue to selectively disrupt metabolic pathways central to tumor survival and immune evasion, but require further optimization for stability, trafficking, and safety.

*Combination with next‐generation immunotherapies*: Beyond checkpoint blockade, combining metabolic nanotherapeutics with emerging immunotherapies—such as CAR‐T cells, bispecific antibodies, or oncolytic viruses—may synergistically restore immune surveillance and enhance tumor eradication.

In summary, metabolic reprogramming is a fundamental driver of immune suppression and therapy resistance in cancer. The convergence of advanced nanotechnology, metabolic precision targeting, and immunotherapy holds significant promise for developing next‐generation cancer treatments. Future progress will require interdisciplinary collaboration across bioengineering, immunology, and cancer metabolism to overcome current translational barriers and realize the full potential of immunometabolic nanomedicine.

## Conflict of Interest

The authors declare no conflict of interest.

## References

[1] L. Xue , A. S. Thatte , D. Mai , R. M. Haley , N. Gong , X. Han , K. Wang , N. C. Sheppard , C. H. June , M. J. Mitchell , Nat. Rev. Mater. 2024, 9, 100.

[2] L. H. Butterfield , Y. G. Najjar , Nat. Rev. Immunol. 2024, 24, 399. 38057451 10.1038/s41577-023-00973-8 PMC11460566

[3] A. Fenis , O. Demaria , L. Gauthier , E. Vivier , E. Narni‐Mancinelli , Nat. Rev. Immunol. 2024, 24, 471. 38273127 10.1038/s41577-023-00982-7

[4] X. Li , J. Gao , C. Wu , C. Wang , R. Zhang , J. He , Z. J. Xia , N. Joshi , J. M. Karp , R. Kuai , Sci. Adv. 2024, 10, adl0479. 10.1126/sciadv.adl0479 PMC11095489 38748805

[5] C. Luri‐Rey , Á. Teijeira , S. K. Wculek , C. de Andrea , C. Herrero , A. Lopez‐Janeiro , M. E. Rodríguez‐Ruiz , I. Heras , M. Aggelakopoulou , P. Berraondo , D. Sancho , I. Melero , Nat. Rev. Cancer 2025, 25, 249. 39881005 10.1038/s41568-024-00785-5

[6] L. Tang , Z. Huang , H. Mei , Y. Hu , Signal Transduct. Target. Ther. 2023, 8, 306. 37591844 10.1038/s41392-023-01521-5 PMC10435569

[7] H. Kluger , J. C. Barrett , J. F. Gainor , O. Hamid , M. Hurwitz , T. LaVallee , R. A. Moss , R. Zappasodi , R. J. Sullivan , H. Tawbi , E. Sharon , J. Immunother. Cancer 2023, 11, 005921. 10.1136/jitc-2022-005921 PMC10016305 36918224

[8] R. Su , Y. Shao , M. Huang , D. Liu , H. Yu , Y. Qiu , Cell Death Discov 2024, 10, 236. 38755125 10.1038/s41420-024-02006-2 PMC11099033

[9] K. DePeaux , G. M. Delgoffe , Nat. Rev. Immunol. 2021, 21, 785. 33927375 10.1038/s41577-021-00541-y PMC8553800

[10] T. Hu , C.‐H. Liu , M. Lei , Q. Zeng , L. Li , H. Tang , N. Zhang , Signal Transduct. Target. Ther. 2024, 9, 268. 39379377 10.1038/s41392-024-01954-6 PMC11461632

[11] M. Flory , P. Bravo , A. Alam , Immunometabolism 2024, 6, 00050. 10.1097/IN9.0000000000000050 PMC11608621 39624362

[12] Y. Shi , H. Zhang , C. Miao , Cell Death Discov 2025, 11, 123. 40155378 10.1038/s41420-025-02403-1 PMC11953409

[13] J. Wang , Y. He , F. Hu , C. Hu , Y. Sun , K. Yang , S. Yang , Int. J. Mol. Sci. 2024, 25, 12223.39596288

[14] S. Ma , Y. Ming , J. Wu , G. Cui , Cell. Mol. Immunol. 2024, 21, 419. 38565887 10.1038/s41423-024-01148-8 PMC11061161

[15] L. Soriano‐Baguet , D. Brenner , Trends Immunol. 2023, 44, 231. 36774330 10.1016/j.it.2023.01.002

[16] D. Vijayan , A. Young , M. W. L. Teng , M. J. Smyth , Nat. Rev. Cancer 2017, 17, 709. 29059149 10.1038/nrc.2017.86

[17] R. D. Leone , J. D. Powell , Nat. Rev. Cancer 2020, 20, 516. 32632251 10.1038/s41568-020-0273-y PMC8041116

[18] M. R. Jennings , D. Munn , J. Blazeck , J. Immunother. Cancer 2021, 9, 003013. 10.1136/jitc-2021-003013 PMC8527165 34667078

[19] B. T. Finicle , V. Jayashankar , A. L. Edinger , Nat. Rev. Cancer 2018, 18, 619. 30097614 10.1038/s41568-018-0048-x

[20] C. Corbet , O. Feron , Nat. Rev. Cancer 2017, 17, 577. 28912578 10.1038/nrc.2017.77

[21] R. J. DeBerardinis , N. S. Chandel , Sci. Adv. 2016, 2, 1600200. 10.1126/sciadv.1600200 PMC4928883 27386546

[22] D. O'Sullivan , D. E. Sanin , E. J. Pearce , E. L. Pearce , Nat. Rev. Immunol. 2019, 19, 324. 30820043 10.1038/s41577-019-0140-9

[23] L. Xia , L. Oyang , J. Lin , S. Tan , Y. Han , N. Wu , P. Yi , L. Tang , Q. Pan , S. Rao , J. Liang , Y. Tang , M. Su , X. Luo , Y. Yang , Y. Shi , H. Wang , Y. Zhou , Q. Liao , Mol. Cancer 2021, 20, 28. 33546704 10.1186/s12943-021-01316-8 PMC7863491

[24] B. Faubert , A. Solmonson , R. J. DeBerardinis , Science 2020, 368. 10.1126/science.aaw5473 PMC7227780 32273439

[25] F. Röhrig , A. Schulze , Nat. Rev. Cancer 2016, 16, 732. 27658529 10.1038/nrc.2016.89

[26] M. Certo , C.‐H. Tsai , V. Pucino , P.‐C. Ho , C. Mauro , Nat. Rev. Immunol. 2021, 21, 151. 32839570 10.1038/s41577-020-0406-2

[27] V. Sreeramkumar , M. Fresno , N. Cuesta , Immunol. Cell Biol. 2012, 90, 579. 21946663 10.1038/icb.2011.75 PMC3389798

[28] S. B. Lacher , J. Dörr , G. P. de Almeida , J. Hönninger , F. Bayerl , A. Hirschberger , A.‐M. Pedde , P. Meiser , L. Ramsauer , T. J. Rudolph , N. Spranger , M. Morotti , A. J. Grimm , S. Jarosch , A. Oner , L. Gregor , S. Lesch , S. Michaelides , L. Fertig , D. Briukhovetska , L. Majed , S. Stock , D. H. Busch , V. R. Buchholz , P. A. Knolle , D. Zehn , D. D. Laniti , S. Kobold , J. P. Böttcher , Nature 2024, 629, 417. 38658748 10.1038/s41586-024-07254-x PMC11078747

[29] R. Geiger , J. C. Rieckmann , T. Wolf , C. Basso , Y. Feng , T. Fuhrer , M. Kogadeeva , P. Picotti , F. Meissner , M. Mann , N. Zamboni , F. Sallusto , A. Lanzavecchia , Cell 2016, 167, 829. 27745970 10.1016/j.cell.2016.09.031 PMC5075284

[30] J. T. Noe , B. E. Rendon , A. E. Geller , L. R. Conroy , S. M. Morrissey , L. E. A. Young , R. C. Bruntz , E. J. Kim , A. Wise‐Mitchell , M. B. de , S. Rizzo , E. R. Relich , B. V. Baby , L. A. Johnson , H. C. Affronti , K. M. McMasters , B. F. Clem , M. S. Gentry , J. Yan , K. E. Wellen , R. C. Sun , R. A. Mitchell , Sci. Adv. 2021, 7, abi8602. 10.1126/sciadv.abi8602 PMC8589316 34767443

[31] N. J. Mullen , P. K. Singh , Nat. Rev. Cancer 2023, 23, 275. 36973407 10.1038/s41568-023-00557-7 PMC10041518

[32] Z. E. Stine , Z. T. Schug , J. M. Salvino , C. V. Dang , Nat. Rev. Drug Discovery 2022, 21, 141. 34862480 10.1038/s41573-021-00339-6 PMC8641543

[33] B. Batchuluun , S. L. Pinkosky , G. R. Steinberg , Nat. Rev. Drug Discovery 2022, 21, 283. 35031766 10.1038/s41573-021-00367-2 PMC8758994

[34] M. Cerezo , S. Rocchi , Cell Death Dis. 2020, 11, 964. 33177494 10.1038/s41419-020-03175-5 PMC7658964

[35] M. D. Buck , D. O'Sullivan , R. I. Klein Geltink , J. D. Curtis , C.‐H. Chang , D. E. Sanin , J. Qiu , O. Kretz , D. Braas , G. J. W. van der Windt , Q. Chen , S. C.‐C. Huang , C. M. O'Neill , B. T. Edelson , E. J. Pearce , H. Sesaki , T. B. Huber , A. S. Rambold , E. L. Pearce , Cell 2016, 166, 63. 27293185 10.1016/j.cell.2016.05.035 PMC4974356

[36] R. D. Leone , L. Zhao , J. M. Englert , I.‐M. Sun , M.‐H. Oh , I.‐H. Sun , M. L. Arwood , I. A. Bettencourt , C. H. Patel , J. Wen , A. Tam , R. L. Blosser , E. Prchalova , J. Alt , R. Rais , B. S. Slusher , J. D. Powell , Science 2019, 366, 1013. 31699883 10.1126/science.aav2588 PMC7023461

[37] M. R. Junttila , F. J. de Sauvage , Nature 2013, 501, 346. 24048067 10.1038/nature12626

[38] M. Demicco , X.‐Z. Liu , K. Leithner , S.‐M. Fendt , Nat. Metab. 2024, 6, 18. 38267631 10.1038/s42255-023-00963-z

[39] J. Hu , J. W. Locasale , J. H. Bielas , J. O'Sullivan , K. Sheahan , L. C. Cantley , M. G. V. Heiden , D. Vitkup , Nat. Biotechnol. 2013, 31, 522. 23604282 10.1038/nbt.2530 PMC3681899

[40] F. P. Canale , C. Basso , G. Antonini , M. Perotti , N. Li , A. Sokolovska , J. Neumann , M. J. James , S. Geiger , W. Jin , J.‐P. Theurillat , K. A. West , D. S. Leventhal , J. M. Lora , F. Sallusto , R. Geiger , Nature 2021, 598, 662. 34616044 10.1038/s41586-021-04003-2

[41] L. Kraehenbuehl , C.‐H. Weng , S. Eghbali , J. D. Wolchok , T. Merghoub , Nat. Rev. Clin. Oncol. 2022, 19, 37. 34580473 10.1038/s41571-021-00552-7

[42] S. Daneshmandi , B. Wegiel , P. Seth , Cancers 2019, 11, 450. 30934955 10.3390/cancers11040450 PMC6521327

[43] C. Chelakkot , V. S. Chelakkot , Y. Shin , K. Song , Int. J. Mol. Sci. 2023, 24, 2606. 36768924 10.3390/ijms24032606 PMC9916680

[44] W. Oh , A. M. J. Kim , D. Dhawan , D. W. Knapp , S.‐O. Lim , Mol. Ther. 2025, 33, 723. 40308191 10.1016/j.ymthe.2024.12.044 PMC11852701

[45] X. Wu , Z. Zhou , K. Li , S. Liu , Adv. Sci. 2024, 11, 2308632. 10.1002/advs.202308632 PMC11040387 38380505

[46] K. Yi , H. Kong , Y. Lao , D. Li , R. L. Mintz , T. Fang , G. Chen , Y. Tao , M. Li , J. Ding , Adv. Mater. 2024, 36, 2300665. 10.1002/adma.202300665 37437039

[47] Z. Tu , Y. Zhong , H. Hu , D. Shao , R. Haag , M. Schirner , J. Lee , B. Sullenger , K. W. Leong , Nat. Rev. Mater. 2022, 7, 557. 35251702 10.1038/s41578-022-00426-z PMC8884103

[48] H. Zhang , Y. Li , J. Huang , L. Shen , Y. Xiong , Acta Pharm. Sin. B 2024, 14, 4717. 39664426 10.1016/j.apsb.2024.07.021 PMC11628863

[49] D. Kolb , N. Kolishetti , B. Surnar , S. Sarkar , S. Guin , A. S. Shah , S. Dhar , ACS Nano 2020, 14, 11055. 32706241 10.1021/acsnano.9b10037

[50] J. R. Melamed , R. S. Riley , D. M. Valcourt , E. S. Day , ACS Nano 2016, 10, 10631. 28024339 10.1021/acsnano.6b07673 PMC5348245

[51] J. Liu , J. Liu , Y. Wang , F. Chen , Y. He , X. Xie , Y. Zhong , C. Yang , Biomaterials 2024, 122919. 39481339 10.1016/j.biomaterials.2024.122919

[52] M.‐R. Chiang , C.‐W. Hsu , W.‐C. Pan , N.‐T. Tran , Y.‐S. Lee , W.‐H. Chiang , Y.‐C. Liu , Y.‐W. Chen , S.‐H. Chiou , S.‐H. Hu , ACS Nano 2025, 19, 2117. 39739571 10.1021/acsnano.4c09525 PMC11760334

[53] Y. Zhou , J. Yuan , K. Xu , S. Li , Y. Liu , N. R. M. E. T. Immunotherapy , ACS Nano 2024, 18, 1846. 38180952 10.1021/acsnano.3c11260

[54] X. Hu , D. Ling , Acta Pharm. Sin. B 2024, 14, 4619. 39525576 10.1016/j.apsb.2024.07.015 PMC11544171

[55] Y. Agarwala , T. A. Brauns , A. E. Sluder , M. C. Poznansky , Y. Gemechu , Trends Immunol. 2024, 45, 486. 38876831 10.1016/j.it.2024.05.006

[56] F. Lazure , A. P. Gomes , Nat. Rev. Cancer 2025, 10.1038/s41568-025-00845-4. 40646271

[57] D. Guo , Y. Meng , G. Zhao , Q. Wu , Z. Lu , Nat. Rev. Cancer 2025, 10.1038/s41568-025-00800-3. 40175621

[58] I. Martínez‐Reyes , N. S. Chandel , Nat. Rev. Cancer 2021, 21, 669. 34272515 10.1038/s41568-021-00378-6

[59] S. Liu , Z. Liu , H. Lei , Y. Miao , J. Chen , Adv. Healthcare Mater. 2025, 14, 2403019. 10.1002/adhm.202403019 39529548

[60] X. Li , T. Sun , C. Jiang , Adv. Mater. 2024, 36, 2309582. 10.1002/adma.202309582 38105387

[61] N. J. Coffey , M. C. Simon , Nat. Rev. Nephrol. 2024, 20, 233. 38253811 10.1038/s41581-023-00800-2 PMC11165401

[62] M. D. Martino , J. C. Rathmell , L. Galluzzi , C. Vanpouille‐Box , Nat. Rev. Immunol. 2024, 24, 654. 38649722 10.1038/s41577-024-01026-4 PMC11365797

[63] M. Liao , D. Yao , L. Wu , C. Luo , Z. Wang , J. Zhang , B. Liu , Acta Pharm. Sin. B 2024, 14, 953. 38487001 10.1016/j.apsb.2023.12.003 PMC10935242

[64] Y. Kidani , H. Elsaesser , M. B. Hock , L. Vergnes , K. J. Williams , J. P. Argus , B. N. Marbois , E. Komisopoulou , E. B. Wilson , T. F. Osborne , T. G. Graeber , K. Reue , D. G. Brooks , S. J. Bensinger , Nat. Immunol. 2013, 14, 489. 23563690 10.1038/ni.2570 PMC3652626

[65] X. Zhang , J.‐J. Lu , A. Abudukeyoumu , D.‐Y. Hou , J. Dong , J.‐N. Wu , L.‐B. Liu , M.‐Q. Li , F. Xie , Front. Oncol. 2022, 12, 933827. 35992779 10.3389/fonc.2022.933827 PMC9389465

[66] J.‐Q. Chen , J. Russo , Acta (BBA) – Rev. Cancer 2012, 1826, 370. 10.1016/j.bbcan.2012.06.004 22750268

[67] C. B. Thompson , K. H. Vousden , R. S. Johnson , W. H. Koppenol , H. Sies , Z. Lu , L. W. S. Finley , C. Frezza , J. Kim , Z. Hu , C. R. Bartman , Nat. Metab. 2023, 5, 1840. 37990075 10.1038/s42255-023-00927-3

[68] N. N. Pavlova , C. B. Thompson , Cell Metab. 2016, 23, 27. 26771115 10.1016/j.cmet.2015.12.006 PMC4715268

[69] J. W. Locasale , Nat. Rev. Cancer 2013, 13, 572. 23822983 10.1038/nrc3557 PMC3806315

[70] B. Jin , Z. Miao , J. Pan , Z. Zhang , Y. Yang , Y. Zhou , Y. Jin , Z. Niu , Q. Xu , Cancer Cell Int. 2025, 25, 78. 40045411 10.1186/s12935-025-03698-x PMC11881340

[71] K.‐C. Kao , S. Vilbois , C.‐H. Tsai , P.‐C. Ho , Nat. Cell Biol. 2022, 24, 1574. 36229606 10.1038/s41556-022-01002-x

[72] M. Peng , N. Yin , S. Chhangawala , K. Xu , C. S. Leslie , M. O. Li , Science 2016, 354, 481. 27708054 10.1126/science.aaf6284 PMC5539971

[73] F. Veglia , E. Sanseviero , D. I. Gabrilovich , Nat. Rev. Immunol. 2021, 21, 485. 33526920 10.1038/s41577-020-00490-y PMC7849958

[74] R. Newton , B. Priyadharshini , L. A. Turka , Nat. Immunol. 2016, 17, 618. 27196520 10.1038/ni.3466 PMC5006394

[75] X. Li , Y. Yang , B. Zhang , X. Lin , X. Fu , Y. An , Y. Zou , J.‐X. Wang , Z. Wang , T. Yu , Signal Transduct. Target. Ther. 2022, 7, 305. 36050306 10.1038/s41392-022-01151-3 PMC9434547

[76] J. Chen , Z. Huang , Y. Chen , H. Tian , P. Chai , Y. Shen , Y. Yao , S. Xu , S. Ge , R. Jia , Signal Transduct. Target. Ther. 2025, 10, 38. 39934144 10.1038/s41392-024-02082-x PMC11814237

[77] S. Chen , Y. Xu , W. Zhuo , L. Zhang , Cancer Lett 2024, 590, 216837. 38548215 10.1016/j.canlet.2024.216837

[78] J. C. García‐Cañaveras , L. Chen , J. D. Rabinowitz , Cancer Res. 2019, 79, 3155. 31171526 10.1158/0008-5472.CAN-18-3726 PMC6606343

[79] B. A. Webb , M. Chimenti , M. P. Jacobson , D. L. Barber , Nat. Rev. Cancer 2011, 11, 671. 21833026 10.1038/nrc3110

[80] Y. Gao , H. Zhou , G. Liu , J. Wu , Y. Yuan , A. Shang , T. Microenvironment , J. Immunol. Res. 2022, 2022, 3119375. 35733921 10.1155/2022/3119375 PMC9207018

[81] A. Brand , K. Singer , G. E. Koehl , M. Kolitzus , G. Schoenhammer , A. Thiel , C. Matos , C. Bruss , S. Klobuch , K. Peter , M. Kastenberger , C. Bogdan , U. Schleicher , A. Mackensen , E. Ullrich , S. Fichtner‐Feigl , R. Kesselring , M. Mack , U. Ritter , M. Schmid , C. Blank , K. Dettmer , P. J. Oefner , P. Hoffmann , S. Walenta , E. K. Geissler , J. Pouyssegur , A. Villunger , A. Steven , B. Seliger , et al., Cell Metab. 2016, 24, 657. 27641098 10.1016/j.cmet.2016.08.011

[82] M. J. Watson , P. D. A. Vignali , S. J. Mullett , A. E. Overacre‐Delgoffe , R. M. Peralta , S. Grebinoski , A. V. Menk , N. L. Rittenhouse , K. DePeaux , R. D. Whetstone , D. A. A. Vignali , T. W. Hand , A. C. Poholek , B. M. Morrison , J. D. Rothstein , S. G. Wendell , G. M. Delgoffe , Nature 2021, 591, 645. 33589820 10.1038/s41586-020-03045-2 PMC7990682

[83] M. Fei , H. Zhang , F. Meng , G. An , J. Tang , J. Tong , L. Xiong , Q. Liu , C. Li , View 2024, 5, 20230053.

[84] J. He , X. Chai , Q. Zhang , Y. Wang , Y. Wang , X. Yang , J. Wu , B. Feng , J. Sun , W. Rui , S. Ze , Y. Fu , Y. Zhao , Y. Zhang , Y. Zhang , M. Liu , C. Liu , M. She , X. Hu , X. Ma , H. Yang , D. Li , S. Zhao , G. Li , Z. Zhang , Z. Tian , Y. Ma , L. Cao , B. Yi , D. Li , et al., Nat. Immunol. 2025, 26, 391. 39905201 10.1038/s41590-024-02068-5

[85] X. Zhao , T. Ren , S. Li , X. Wang , R. Hou , Z. Guan , D. Liu , J. Zheng , M. Shi , Int. J. Biol. Sci. 2024, 20, 5109. 39430253 10.7150/ijbs.99680 PMC11489172

[86] H. Tao , X. Zhong , A. Zeng , L. Song , Front. Immunol. 2023, 14, 1208870. 37564659 10.3389/fimmu.2023.1208870 PMC10411982

[87] P. Apostolova , E. L. Pearce , Trends Immunol. 2022, 43, 969. 36319537 10.1016/j.it.2022.10.005 PMC10905416

[88] F. Hirschhaeuser , U. G. A. Sattler , W. Mueller‐Klieser , Cancer Res. 2011, 71, 6921. 22084445 10.1158/0008-5472.CAN-11-1457

[89] M. Liaghat , S. Ferdousmakan , S. H. Mortazavi , S. Yahyazadeh , A. Irani , S. Banihashemi , F. S. S. Asl , A. Akbari , F. Farzam , F. Aziziyan , M. Bakhtiyari , M. J. Arghavani , H. Zalpoor , M. Nabi‐Afjadi , Cell Commun. Signal. 2024, 22, 575. 39623377 10.1186/s12964-024-01957-4 PMC11610171

[90] F. Kitamura , T. Semba , N. Yasuda‐Yoshihara , K. Yamada , A. Nishimura , J. Yamasaki , O. Nagano , T. Yasuda , A. Yonemura , Y. Tong , H. Wang , T. Akiyama , K. Matsumura , N. Uemura , R. Itoyama , L. Bu , L. Fu , X. Hu , F. Wei , K. Mima , K. Imai , H. Hayashi , Y. Yamashita , Y. Miyamoto , H. Baba , T. Ishimoto , JCI Insight 2023, 8, 163022. 10.1172/jci.insight.163022 PMC10619496 37733442

[91] A. Wu , D. Lee , W.‐C. Xiong , Int. J. Mol. Sci. 2023, 24, 13398.37686202

[92] J. M. Macharia , Z. Kaposztas , T. Varjas , F. Budán , A. Zand , I. Bodnar , R. L. Bence , Biomed. Pharmacother. 2023, 160, 114371. 36758316 10.1016/j.biopha.2023.114371

[93] G. Claps , S. Faouzi , V. Quidville , F. Chehade , S. Shen , S. Vagner , C. Robert , Nat. Rev. Clin. Oncol. 2022, 19, 749. 36207413 10.1038/s41571-022-00686-2

[94] P. Swietach , E. Boedtkjer , S. F. Pedersen , Nat. Rev. Cancer 2023, 23, 825. 37884609 10.1038/s41568-023-00628-9

[95] Z.‐N. Ling , Y.‐F. Jiang , J.‐N. Ru , J.‐H. Lu , B. Ding , J. Wu , Signal Transduct. Target. Ther. 2023, 8, 345. 37699892 10.1038/s41392-023-01569-3 PMC10497558

[96] G. Bergers , S.‐M. Fendt , Nat. Rev. Cancer 2021, 21, 162. 33462499 10.1038/s41568-020-00320-2 PMC8733955

[97] M. You , Z. Xie , N. Zhang , Y. Zhang , D. Xiao , S. Liu , W. Zhuang , L. Li , Y. Tao , Target. Ther. 2023, 8, 196. 10.1038/s41392-023-01442-3 PMC10172373 37164974

[98] H. Lemos , L. Huang , G. C. Prendergast , A. L. Mellor , Nat. Rev. Cancer 2019, 19, 162. 30696923 10.1038/s41568-019-0106-z

[99] C. T. Hensley , A. T. Wasti , R. J. DeBerardinis , J. Clin. Investig. 2013, 123, 3678. 23999442 10.1172/JCI69600 PMC3754270

[100] E. L. Lieu , T. Nguyen , S. Rhyne , J. Kim , Exp. Mol. Med. 2020, 52, 15. 31980738 10.1038/s12276-020-0375-3 PMC7000687

[101] C. R. Green , M. Wallace , A. S. Divakaruni , S. A. Phillips , A. N. Murphy , T. P. Ciaraldi , C. M. Metallo , Nat. Chem. Biol. 2016, 12, 15. 26571352 10.1038/nchembio.1961 PMC4684771

[102] M. Butler , L. T. van der Meer , F. N. van Leeuwen , Trends Endocrinol. Metab. 2021, 32, 367. 33795176 10.1016/j.tem.2021.03.003

[103] Y. Zhang , M. Morar , S. E. Ealick , Cell. Mol. Life Sci. 2008, 65, 3699. 18712276 10.1007/s00018-008-8295-8 PMC2596281

[104] O. Shuvalov , A. Petukhov , A. Daks , O. Fedorova , E. Vasileva , N. A. Barlev , Oncotarget 2017, 8, 23955. 28177894 10.18632/oncotarget.15053 PMC5410357

[105] G. K. Balendiran , R. Dabur , D. Fraser , Cell Biochem. Funct. 2004, 22, 343. 15386533 10.1002/cbf.1149

[106] J. Fan , J. Ye , J. J. Kamphorst , T. Shlomi , C. B. Thompson , J. D. Rabinowitz , Nature 2014, 510, 298. 24805240 10.1038/nature13236 PMC4104482

[107] Y. D. Bhutia , E. Babu , S. Ramachandran , V. Ganapathy , Cancer Res. 2015, 75, 1782. 25855379 10.1158/0008-5472.CAN-14-3745

[108] H.‐Q. Ju , J.‐F. Lin , T. Tian , D. Xie , R.‐H. Xu , Signal Transduct. Target. Ther. 2020, 5, 231. 33028807 10.1038/s41392-020-00326-0 PMC7542157

[109] E. N. Arner , J. C. Rathmell , Cancer Cell 2023, 41, 421. 36801000 10.1016/j.ccell.2023.01.009 PMC10023409

[110] G. Ma , Z. Zhang , P. Li , Z. Zhang , M. Zeng , Z. Liang , D. Li , L. Wang , Y. Chen , Y. Liang , H. Niu , Cell Commun. Signal. 2022, 20, 114. 35897036 10.1186/s12964-022-00909-0 PMC9327201

[111] J. Chen , L. Cui , S. Lu , S. Xu , Cell Death Dis. 2024, 15, 42. 38218942 10.1038/s41419-024-06435-w PMC10787762

[112] X. Liu , B. Ren , J. Ren , M. Gu , L. You , Y. Zhao , Cell Commun. Signal. 2024, 22, 380. 39069612 10.1186/s12964-024-01760-1 PMC11285422

[113] L. F. Campesato , S. Budhu , J. Tchaicha , C.‐H. Weng , M. Gigoux , I. J. Cohen , D. Redmond , L. Mangarin , S. Pourpe , C. Liu , R. Zappasodi , D. Zamarin , J. Cavanaugh , A. C. Castro , M. G. Manfredi , K. McGovern , T. Merghoub , J. D. Wolchok , Nat. Commun. 2020, 11, 4011. 32782249 10.1038/s41467-020-17750-z PMC7419300

[114] J. Kim , J. Li , J. Wei , S. A. Lim , Immune Netw 2025, 25, 13. 10.4110/in.2025.25.e13 PMC11896657 40078783

[115] Y. Zheng , Y. Yao , T. Ge , S. Ge , R. Jia , X. Song , A. Zhuang , J. Exp. Clin. Cancer Res. 2023, 42, 291. 37924140 10.1186/s13046-023-02845-4 PMC10623764

[116] S. Pan , M. Fan , Z. Liu , X. Li , H. Wang , Int. J. Oncol. 2020, 58, 158. 33491748 10.3892/ijo.2020.5158 PMC7864012

[117] M. Laplante , D. M. Sabatini , Cell 2012, 149, 274. 22500797 10.1016/j.cell.2012.03.017 PMC3331679

[118] A. V. Belikov , B. Schraven , L. Simeoni , J. Biomed. Sci. 2015, 22, 85. 26471060 10.1186/s12929-015-0194-3 PMC4608155

[119] X. Chen , M. Song , B. Zhang , Y. Zhang , Oxidative Med. Cell. Longev. 2016, 2016, 1580967. 10.1155/2016/1580967 PMC4980531 27547291

[120] H. Kang , H. Kim , S. Lee , H. Youn , B. Youn , Int. J. Mol. Sci. 2019, 20, 2042. 31027222 10.3390/ijms20082042 PMC6514888

[121] T. Shiomi , Y. Okada , Cancer Metastasis Rev. 2003, 22, 145. 12784993 10.1023/a:1023039230052

[122] Y. Li , F. Liu , Q. Cai , L. Deng , Q. Ouyang , X. H.‐F. Zhang , J. Zheng , Signal Transduct. Target. Ther. 2025, 10, 57. 39979279 10.1038/s41392-025-02148-4 PMC11842613

[123] E. Xu , B. Ji , K. Jin , Y. Chen , Front. Oncol. 2023, 13, 1220638. 37637065 10.3389/fonc.2023.1220638 PMC10448767

[124] E. J. Kay , K. Paterson , C. Riera‐Domingo , D. Sumpton , J. H. M. Däbritz , S. Tardito , C. Boldrini , J. R. Hernandez‐Fernaud , D. Athineos , S. Dhayade , E. Stepanova , E. Gjerga , L. J. Neilson , S. Lilla , A. Hedley , G. Koulouras , G. McGregor , C. Jamieson , R. M. Johnson , M. Park , K. Kirschner , C. Miller , J. J. Kamphorst , F. Loayza‐Puch , J. Saez‐Rodriguez , M. Mazzone , K. Blyth , M. Zagnoni , S. Zanivan , Nat. Metab. 2022, 4, 693. 35760868 10.1038/s42255-022-00582-0 PMC9236907

[125] R. E. Oberkersch , M. M. Santoro , Vasc. Pharmacol. 2019, 112, 17. 10.1016/j.vph.2018.11.001 30423448

[126] J. K. C. Kreß , C. Jessen , A. Hufnagel , W. Schmitz , T. N. X. da Silva , A. F. dos Santos , L. Mosteo , C. R. Goding , J. P. F. Angeli , S. Meierjohann , Cell Rep. 2023, 42, 112724. 37410595 10.1016/j.celrep.2023.112724

[127] L. Yang , Z. Chu , M. Liu , Q. Zou , J. Li , Q. Liu , Y. Wang , T. Wang , J. Xiang , B. Wang , J. Hematol. Oncol. 2023, 16, 59. 37277776 10.1186/s13045-023-01453-1 PMC10240810

[128] W. Khan , D. Augustine , R. S. Rao , S. Patil , K. H. Awan , S. V. Sowmya , V. C. Haragannavar , K. Prasad , J. Carcinog. 2021, 20, 4. 34321955 10.4103/jcar.JCar_15_20 PMC8312377

[129] M. Martin‐Perez , U. Urdiroz‐Urricelqui , C. Bigas , S. A. Benitah , Cell Metab. 2022, 34, 1675. 36261043 10.1016/j.cmet.2022.09.023

[130] D. D. Shi , M. R. Savani , K. G. Abdullah , S. K. McBrayer , Trends Cancer 2023, 9, 624. 37173188 10.1016/j.trecan.2023.04.008 PMC10967252

[131] Y. Liu , Y. Sun , Y. Guo , X. Shi , X. Chen , W. Feng , L.‐L. Wu , J. Zhang , S. Yu , Y. Wang , Y. Shi , Int. J. Biol. Sci. 2023, 19, 897. 36778129 10.7150/ijbs.81609 PMC9910000

[132] I. San‐Millán , Antioxidants 2023, 12, 782. 37107158 10.3390/antiox12040782 PMC10135185

[133] L. A. Broadfield , A. A. Pane , A. Talebi , J. V. Swinnen , S.‐M. Fendt , Dev. Cell 2021, 56, 1363. 33945792 10.1016/j.devcel.2021.04.013

[134] J. A. Menendez , R. Lupu , Nat. Rev. Cancer 2007, 7, 763. 17882277 10.1038/nrc2222

[135] Q. Long , Y. Yi , J. Qiu , C. Xu , P. Huang , Tumor Biol 2014, 35, 3855. 10.1007/s13277-013-1510-8 24430360

[136] S. F. Jones , J. R. Infante , Clin. Cancer Res. 2015, 21, 5434. 26519059 10.1158/1078-0432.CCR-15-0126

[137] T. Mashima , S. Sato , S. Okabe , S. Miyata , M. Matsuura , Y. Sugimoto , T. Tsuruo , H. Seimiya , Cancer Sci. 2009, 100, 1556. 19459852 10.1111/j.1349-7006.2009.01203.x PMC11158289

[138] M. P. Wymann , R. Schneiter , Nat. Rev. Mol. Cell Biol. 2008, 9, 162. 18216772 10.1038/nrm2335

[139] Y. Guri , M. Colombi , E. Dazert , S. K. Hindupur , J. Roszik , S. Moes , P. Jenoe , M. H. Heim , I. Riezman , H. Riezman , M. N. Hall , Cancer Cell 2017, 32, 807. 29232555 10.1016/j.ccell.2017.11.011

[140] H.‐R. Jin , J. Wang , Z.‐J. Wang , M.‐J. Xi , B.‐H. Xia , K. Deng , J.‐L. Yang , J. Hematol. Oncol. 2023, 16, 103. 37700339 10.1186/s13045-023-01498-2 PMC10498649

[141] J. Saravia , H. Chi , Oncogene 2025, 44, 2011. 40468052 10.1038/s41388-025-03458-1 PMC12167712

[142] G. Marelli , N. Morina , F. Portale , M. Pandini , M. Iovino , G. D. Conza , P.‐C. Ho , D. D. Mitri , J. Immunother. Cancer 2022, 10, 004584. 10.1136/jitc-2022-004584 PMC9263925 35798535

[143] K. M. Nieman , H. A. Kenny , C. V. Penicka , A. Ladanyi , R. Buell‐Gutbrod , M. R. Zillhardt , I. L. Romero , M. S. Carey , G. B. Mills , G. S. Hotamisligil , S. D. Yamada , M. E. Peter , K. Gwin , E. Lengyel , Nat. Med. 2011, 17, 1498. 22037646 10.1038/nm.2492 PMC4157349

[144] J. Gong , Y. Lin , H. Zhang , C. Liu , Z. Cheng , X. Yang , J. Zhang , Y. Xiao , N. Sang , X. Qian , L. Wang , X. Cen , X. Du , Y. Zhao , Cell Death Dis. 2020, 11, 267. 32327627 10.1038/s41419-020-2434-z PMC7181758

[145] D. Mossmann , S. Park , M. N. Hall , Nat. Rev. Cancer 2018, 18, 744. 30425336 10.1038/s41568-018-0074-8

[146] C. Yang , Y. Zhao , L. Wang , Z. Guo , L. Ma , R. Yang , Y. Wu , X. Li , J. Niu , Q. Chu , Y. Fu , B. Li , Nat. Cell Biol. 2023, 25, 836. 37291265 10.1038/s41556-023-01146-4

[147] W. B. Parker , Chem. Rev. 2009, 109, 2880. 19476376 10.1021/cr900028p PMC2827868

[148] J. Liu , S. Hong , J. Yang , X. Zhang , Y. Wang , H. Wang , J. Peng , L. Hong , J. Ovarian Res. 2022, 15, 93. 35964092 10.1186/s13048-022-01022-z PMC9375293

[149] W. Wang , J. Cui , H. Ma , W. Lu , J. Huang , Oncol 2021, 11, 684961. 10.3389/fonc.2021.684961 PMC8194085 34123854

[150] Y.‐C. Nieh , Y.‐T. Chou , Y.‐T. Chou , C.‐Y. Wang , S.‐X. Lin , S.‐C. Ciou , C.‐H. Yuh , H.‐D. Wang , Int. J. Mol. Sci. 2022, 23, 7883.35887232

[151] A. Formentini , S. Sander , S. Denzer , J. Straeter , D. Henne‐Bruns , M. Kornmann , Int. J. Color. Dis. 2007, 22, 49. 10.1007/s00384-006-0111-z 16538493

[152] Y. Zheng , R. Xu , X. Chen , Y. Lu , J. Zheng , Y. Lin , P. Lin , X. Zhao , L. Cui , Cell Death Dis. 2024, 15, 775. 39461979 10.1038/s41419-024-07122-6 PMC11513100

[153] H. Wu , Y. Gong , P. Ji , Y. Xie , Y.‐Z. Jiang , G. Liu , J. Hematol. Oncol. 2022, 15, 45. 35477416 10.1186/s13045-022-01263-x PMC9044757

[154] X. Xu , Z. Chen , C. R. Bartman , X. Xing , K. Olszewski , J. D. Rabinowitz , Cell Chem. Biol. 2024, 31, 932. 38759619 10.1016/j.chembiol.2024.04.007 PMC12118570

[155] J. Wu , Y. Rong , T. Li , C. M. Wilson , Y. He , D. Chen , J. Han , X. Zhang , Front. Immunol. 2024, 15, 1412057. 38715612 10.3389/fimmu.2024.1412057 PMC11074342

[156] C. Xia , S. Yin , K. K. W. To , L. Fu , Mol. Cancer 2023, 22, 44. 36859386 10.1186/s12943-023-01733-x PMC9979453

[157] P. Gralewska , A. Gajek , A. Marczak , A. Rogalska , J. Hematol. Oncol. 2020, 13, 39. 32316968 10.1186/s13045-020-00874-6 PMC7175546

[158] F. F. Diehl , T. P. Miettinen , R. Elbashir , C. S. Nabel , A. M. Darnell , B. T. Do , S. R. Manalis , C. A. Lewis , M. G. V. Heiden , Nat. Cell Biol. 2022, 24, 1252. 35927450 10.1038/s41556-022-00965-1 PMC9359916

[159] W.‐X. Zong , J. D. Rabinowitz , E. White , Mol. Cell 2016, 61, 667. 26942671 10.1016/j.molcel.2016.02.011 PMC4779192

[160] G. S. Gorman , P. F. Chinnery , S. DiMauro , M. Hirano , Y. Koga , R. McFarland , A. Suomalainen , D. R. Thorburn , M. Zeviani , D. M. Turnbull , Nat. Rev. Dis. Prim. 2016, 2, 16080. 27775730 10.1038/nrdp.2016.80

[161] D. C. Wallace , Nat. Rev. Cancer 2012, 12, 685. 23001348 10.1038/nrc3365 PMC4371788

[162] P. K. Kopinski , L. N. Singh , S. Zhang , M. T. Lott , D. C. Wallace , Nat. Rev. Cancer 2021, 21, 431. 34045735 10.1038/s41568-021-00358-w

[163] W. Gamal , M. Mediavilla‐Varela , V. Kunta , E. Sahakian , J. Pinilla‐Ibarz , Cell Dev. Biol. 2025, 13, 1577081. 10.3389/fcell.2025.1577081 PMC12043688 40313718

[164] L. Simula , M. Fumagalli , L. Vimeux , I. Rajnpreht , P. Icard , G. Birsen , D. An , F. Pendino , A. Rouault , N. Bercovici , D. Damotte , A. Lupo‐Mansuet , M. Alifano , M.‐C. Alves‐Guerra , E. Donnadieu , Nat. Commun. 2024, 15, 2203. 38467616 10.1038/s41467-024-46377-7 PMC10928223

[165] E. M. Steinert , B. F. Bruza , V. D. Danchine , R. A. Grant , K. Vasan , A. Kharel , Y. Zhang , W. Cui , M. Szibor , S. E. Weinberg , N. S. Chandel , Nat. Immunol. 2025, 26, 1267. 40670617 10.1038/s41590-025-02202-x PMC12307223

[166] Q. Wang , X. Yin , X. Huang , L. Zhang , H. Lu , Front. Immunol. 2024, 15, 1428596. 39464876 10.3389/fimmu.2024.1428596 PMC11502362

[167] J. Xu , Y. Zheng , Y. Zhao , Y. Zhang , H. Li , A. Zhang , X. Wang , W. Wang , Y. Hou , J. Wang , Front. Immunol. 2022, 13, 817572. 35273600 10.3389/fimmu.2022.817572 PMC8901997

[168] S. Fulda , L. Galluzzi , G. Kroemer , Nat. Rev. Drug Discovery 2010, 9, 447. 20467424 10.1038/nrd3137

[169] J. Wang , W. Cao , J. Huang , Y. Zhou , R. Zheng , Y. Lou , J. Yang , J. Tang , M. Ye , Z. Hong , J. Wu , H. Ding , Y. Zhang , J. Sheng , X. Lu , P. Xu , X. Lu , X. Bai , T. Liang , Q. Zhang , 2025, arXiv preprint arXiv:2503, 17738.

[170] A. N. Lane , R. M. Higashi , T. W.‐M. Fan , Genes Dis 2020, 7, 185. 32215288 10.1016/j.gendis.2019.10.007 PMC7083762

[171] F. Sanchez‐Vega , M. Mina , J. Armenia , W. K. Chatila , A. Luna , K. C. La , S. Dimitriadoy , D. L. Liu , H. S. Kantheti , S. Saghafinia , D. Chakravarty , F. Daian , Q. Gao , M. H. Bailey , W.‐W. Liang , S. M. Foltz , I. Shmulevich , L.i Ding , Z. Heins , A. Ochoa , B. Gross , J. Gao , H. Zhang , R. Kundra , C. Kandoth , I. Bahceci , L. Dervishi , U. Dogrusoz , W. Zhou , H. Shen , et al., Cell 2018, 173, 321.29625050

[172] S. K. Biswas , Immunity 2015, 43, 435. 26377897 10.1016/j.immuni.2015.09.001

[173] H. Wu , Y. Han , Y. R. Sillke , H. Deng , S. Siddiqui , C. Treese , F. Schmidt , M. Friedrich , J. Keye , J. Wan , Y. Qin , A. A. Kühl , Z. Qin , B. Siegmund , R. Glauben , EMBO Mol. Med. 2019, 11, EMMM201910698. 10.15252/emmm.201910698 PMC6835560 31602788

[174] P. Yang , H. Qin , Y. Li , A. Xiao , E. Zheng , H. Zeng , C. Su , X. Luo , Q. Lu , M. Liao , L. Zhao , L. Wei , Z. Varghese , J. F. Moorhead , Y. Chen , X. Z. Ruan , Nat. Commun. 2022, 13, 5782. 36184646 10.1038/s41467-022-33349-y PMC9527239

[175] V. K. Pandey , K. Premkumar , P. Kundu , B. S. Shankar , Life Sci. 2024, 350, 122751. 38797363 10.1016/j.lfs.2024.122751

[176] G. J. Yoshida , J. Exp. Clin. Cancer Res. 2015, 34, 111. 26445347 10.1186/s13046-015-0221-y PMC4595070

[177] K. Wang , L. Li , G. Liang , H. Xiao , L. Zhang , T. Liu , Biomaterials 2025, 319, 123178. 39978048 10.1016/j.biomaterials.2025.123178

[178] D.‐W. Wang , X.‐H. Ren , Y.‐J. Ma , F.‐Q. Wang , X.‐W. He , W.‐Y. Li , Y.‐K. Zhang , J. Colloid Interface Sci. 2025, 683, 890. 10.1016/j.jcis.2024.12.227 39755015

[179] X. Wu , J. Shen , X. Jiang , H. Han , Z. Li , Y. Xiang , D. Yuan , J. Shi , Chem. Eng. J. 2024, 493, 152479.

[180] C. Caro , J. M. Paez‐Muñoz , M. P. Leal , M. Carayol , M. Feijoo‐Cuaresma , M. L. García‐Martín , Adv. Healthcare Mater. 2025, 14, 2404391. 10.1002/adhm.202404391 39578332

[181] Y. Wang , L. Zang , L. Guan , X. Ren , Z. Xia , Z. Li , Z. Meng , H. Lian , Chem. Eng. J. 2025, 503, 158442.

[182] J. Yan , R. Bhadane , M. Ran , X. Ma , Y. Li , D. Zheng , O. M. H. Salo‐Ahen , H. Zhang , Nat. Commun. 2024, 15, 3684. 38693181 10.1038/s41467-024-48149-9 PMC11063048

[183] B. Wu , Z. Wang , J. Liu , N. Li , X. Wang , H. Bai , C. Wang , J. Shi , S. Zhang , J. Song , Y. Li , G. Nie , Nat. Commun. 2024, 15, 10526. 39627234 10.1038/s41467-024-54963-y PMC11615375

[184] G. Chen , L. Lin , Z. Mai , Y. Tang , Q. Zhang , G. Chen , Z. Li , J. Zhang , Y. Wang , Y. Yang , Z. Yu , ACS Nano 2024, 18, 19875. 10.1021/acsnano.4c07213 39034461

[185] X. Han , D. Xiang , J. Li , S. Liao , D. Tang , Y. Han , M. Xu , W. Bi , H. Xiao , Nano Today 2024, 54, 102057.

[186] Y. Liu , H. Li , Y. Hao , L. Huang , X. Li , J. Zou , S. Zhang , X. Yang , H. Chen , Y. Guo , Y. Guan , Z. Zhang , Small 2025, 21, 2406870. 10.1002/smll.202406870 39390849

[187] R. Liu , L. Guo , D. Shi , X. Sun , M. Shang , Y. Zhao , X. Wang , Y. Yang , S. Xiao , J. Li , J. Controlled Release 2025, 383, 113797. 10.1016/j.jconrel.2025.113797 40318807

[188] J. Wang , W. Jiang , M. Fang , C. Du , X. Guo , X. Qiu , X. Wang , Y. Luo , P. Tu , C. Cheng , P. Li , H. Ran , J. Ren , Chem. Eng. J. 2023, 477, 147163.

[189] L. Hu , S. Huang , G. Chen , B. Li , T. Li , M. Lin , Y. Huang , Z. Xiao , X. Shuai , Z. Su , ACS Appl. Mater. Interfaces 2022, 14, 31625. 35796429 10.1021/acsami.2c05841

[190] Z. Zhang , X. Li , W. Liu , G. Chen , J. Liu , Q. Ma , P. Hou , L. Liang , C. Liu , Drug Resist. Updat. 2024, 73, 101060. 38309140 10.1016/j.drup.2024.101060

[191] L. Chen , J. Yang , L. Jia , X. Wei , H. Wang , Z. Liu , S. Jiang , P. Li , Y. Zhou , H. Wang , N. Si , B. Bian , Q. Zhao , H. Zhao , J. Nanobiotechnology 2025, 23, 19. 39819479 10.1186/s12951-024-03084-1 PMC11740360

[192] Z. Pan , X. Lu , X. Hu , R. Yu , Y. Che , J. Wang , L. Xiao , J. Chen , X. Yi , Z. Tan , F. Li , D. Ling , P. Huang , M. Ge , J. Controlled Release 2024, 369, 517. 10.1016/j.jconrel.2024.03.057 38569942

[193] Z. Wang , M. Wu , Y. Jiang , J. Zhou , S. Chen , Q. Wang , H. Sun , Y. Deng , Z. Zhou , M. Sun , J. Controlled Release 2025, 380, 362. 10.1016/j.jconrel.2025.01.046 39832746

[194] Z. Sun , Y. Liu , T. Zeng , H. Zuo , Q. Hu , Z. Tian , Q. Wang , B. Zhang , Z. Tang , W. Chen , Adv. Funct. Mater. 2025, 35, 2412705.

[195] L. Kou , X. Jiang , Y. Tang , X. Xia , Y. Li , A. Cai , H. Zheng , H. Zhang , V. Ganapathy , Q. Yao , R. Chen , Bioact. Mater. 2022, 9, 15. 34820552 10.1016/j.bioactmat.2021.07.009 PMC8586589

[196] Y. Wang , Q. Chen , Y. Luo , Y. Qu , X. Li , H. Song , C. Li , Y. Zhang , T. Sun , C. Jiang , ACS Nano 2024, 18, 34996. 39666893 10.1021/acsnano.4c13425

[197] S.‐Y. Yoo , H. Y. Kim , D. H. Kim , W. S. Shim , S. M. Lee , D. H. Lee , J. M. Koo , J. H. Yoo , S. Koh , J. C. Park , J. Yu , J. S. Jeon , M.‐J. Baek , D.‐D. Kim , J.‐Y. Lee , S. J. Oh , S. K. Kim , J.‐Y. Lee , K. W. Kang , J. Controlled Release 2024, 375, 574. 10.1016/j.jconrel.2024.09.029 39293529

[198] Y. Liu , S. Dong , W. Hu , Q. Chen , S. Zhang , K. Song , Z. Han , M. Li , Z. Han , W. Liu , X. Zhang , Bioact. Mater. 2024, 36, 157. 38463554 10.1016/j.bioactmat.2024.02.007 PMC10924166

[199] S. Huang , H. Le , G. Hong , G. Chen , F. Zhang , L. Lu , X. Zhang , Y. Qiu , Z. Wang , Q. Zhang , G. Ouyang , J. Shen , Acta Biomater. 2022, 148, 244. 35709941 10.1016/j.actbio.2022.06.017

[200] L. Zhou , W. Feng , Y. Mao , Y. Chen , X. Zhang , Bioact. Mater. 2023, 24, 26. 36582345 10.1016/j.bioactmat.2022.11.020 PMC9761609

[201] N. Zhang , W. Ping , K. Rao , Z. Zhang , R. Huang , D. Zhu , G. Li , S. Ning , J. Controlled Release 2024, 371, 204. 10.1016/j.jconrel.2024.05.045 38810704

[202] Z. Li , X. Li , Y. Lu , X. Zhu , W. Zheng , K. Chen , S. Liu , J. Wu , W. Guan , Small 2023, 2305174. 10.1002/smll.202305174 37875654

[203] Y. Yang , B. Zhang , Y. Xu , W. Zhu , Z. Zhu , X. Zhang , W. Wu , J. Chen , Z. Yu , Bioact. Mater. 2024, 42, 178. 39285910 10.1016/j.bioactmat.2024.08.028 PMC11402546

[204] S. Chen , Y. Jiang , J. Zheng , P. Li , M. Liu , Y. Zhu , S. Zhu , S. Chang , J. Controlled Release 2025, 379, 89. 10.1016/j.jconrel.2024.12.073 39756690

[205] F. Martins , R. Arada , H. Barros , P. Matos , J. Ramalho , V. Ceña , V. D. B. Bonifácio , L. G. Gonçalves , J. Serpa , Cancer Gene Ther. 2025, 32, 690. 40289180 10.1038/s41417-025-00906-8 PMC12245715

[206] Y. Xu , Z. Yu , H. Fu , Y. Guo , P. Hu , J. Shi , ACS Appl. Mater. Interfaces 2022, 14, 21836. 35512029 10.1021/acsami.2c00111

[207] Y. Lv , M. Li , L. Weng , H. Huang , Y. Mao , D. A. Yang , Q. Wei , M. Zhao , Q. Wei , K. Rui , X. Han , W. Fan , X. Cai , P. Cao , M. Cao , J. Exp. Clin. Cancer Res. 2023, 42, 322. 38012650 10.1186/s13046-023-02888-7 PMC10683135

[208] N. U. Deshpande , A. Bianchi , H. Amirian , I. D. C. Silva , C. I. Rafie , B. Surnar , K. Rajkumar , A. Ashokan , I. C. Ogobuiro , M. Patel , E. Stelekati , S. Dhar , J. Datta , Cancer Immunol Immunother 2025, 74, 247. 40549154 10.1007/s00262-025-04096-y PMC12185796

[209] Y. Chen , X. Shu , J.‐Y. Guo , Y. Xiang , S.‐Y. Liang , J.‐M. Lai , J.‐Y. Zhou , L.‐H. Liu , P. Wang , J. Controlled Release 2024, 367, 248. 10.1016/j.jconrel.2024.01.045 38272398

[210] H. Wang , X. Jin , Y. Gao , X. He , Y. Xu , H. Mu , Y. Jiang , Z. Wang , C. Yu , T. Zhang , Y. Hua , Z. Cai , J. Xu , X. Ma , W. Sun , Cancer Nanotechnol. 2023, 14, 50.

[211] S. Paudyal , F. A. Vallejo , E. K. Cilingir , Y. Zhou , K. J. Mintz , Y. Pressman , J. Gu , S. Vanni , R. M. Graham , R. M. Leblanc , ACS Appl. Bio Mater. 2022, 5, 3300. 10.1021/acsabm.2c00309 35771033

[212] X. Xie , Y. Feng , H. Zhang , Q. Su , T. Song , G. Yang , N. Li , X. Wei , T. Li , X. Qin , S. Li , C. Wu , X. Zhang , G. Wang , Y. Liu , H. Yang , Bioact. Mater. 2022, 16, 107. 35386322 10.1016/j.bioactmat.2022.03.008 PMC8958467

[213] W. Hu , B. Ye , G. Yu , H. Yang , H. Wu , Y. Ding , F. Huang , W. Wang , Z. Mao , Adv. Sci. 2024, 11, 2305382. 10.1002/advs.202305382 PMC11132052 38493499

[214] M. Li , Y. Liu , Y. Zhang , N. Yu , J. Li , Adv. Sci. 2023, 10, 2305150. 10.1002/advs.202305150 PMC10724419 37870196

[215] J. Li , Y. Dai , T. Wang , X. Zhang , P. Du , Y. Dong , Z. Jiao , J. Controlled Release 2025, 380, 615. 10.1016/j.jconrel.2025.02.021 39947402

[216] Z. Li , Q. Pei , M. Zhao , Z. Xie , M. Zheng , Adv. Funct. Mater. 2024, 34, 2312500.

[217] J. Song , M. Cheng , Y. Xie , K. Li , X. Zang , J. Nanobiotechnology 2023, 21, 93. 36927803 10.1186/s12951-023-01842-1 PMC10018933

[218] D. Wu , J. Zhou , Z. Zhang , Y. Cao , K. Ping , S. Qi , J. Du , G. Yu , Adv. Sci. 2025, 12, 2408518. 10.1002/advs.202408518 PMC11923969 39887941

[219] J. Choi , B. Park , J. Y. Park , D. Shin , S. Lee , H. Y. Yoon , K. Kim , S. H. Kim , Y. Kim , Y. Yang , M. K. Shim , Adv. Mater. 2024, 36, 2405475. 10.1002/adma.202405475 38898702

[220] Y. Wang , L. Yang , C. Yan , Y. Du , T. Li , W. Yang , L. Lei , B. He , H. Gao , N. A. Peppas , J. Cao , Sci. Adv. 2024, 10, adn8079. 10.1126/sciadv.adn8079 PMC11192078 38905336

[221] L. Lei , S. Cai , Y. Zhang , L. Yang , J. Deng , H. Mei , X. Zhang , K. Zhang , B. He , J. Cao , Adv. Funct. Mater. 2022, 32.

[222] M. Han , S. Zhou , Z. Liao , C. Zishan , X. Yi , C. Wu , D. Zhang , Y. He , K. W. Leong , Y. Zhong , Biomaterials 2024, 122934. 39509856 10.1016/j.biomaterials.2024.122934

[223] J. Du , T. Jia , F. Li , Y. Li , Q. Wang , L. He , H. Ågren , G. Chen , Adv. Funct. Mater. 2024, 34, 2401272.

[224] R. Cillari , R. C. Acúrcio , A. Barateiro , H. F. Florindo , N. Mauro , G. Cavallaro , J. Controlled Release 2025, 381, 113575. 10.1016/j.jconrel.2025.02.071 40024343

[225] L. Du , H. He , Z. Xiao , H. Xiao , Y. An , H. Zhong , M. Lin , X. Meng , S. Han , X. Shuai , Small 2022, 18, 2107732. 10.1002/smll.202107732 35218310

[226] W. Wu , Y. Pu , B. Zhou , Y. Shen , S. Gao , M. Zhou , J. Shi , J. Am. Chem. Soc. 2022, 144, 19038. 36215038 10.1021/jacs.2c07872

[227] Y. Qin , N. Rouatbi , J. T.‐W. Wang , R. Baker , J. Spicer , A. A. Walters , K. T. Al‐Jamal , J. Controlled Release 2024, 369, 251. 10.1016/j.jconrel.2024.03.018 PMC11464404 38493950

[228] L. N. M. Nguyen , W. Ngo , Z. P. Lin , S. Sindhwani , P. MacMillan , S. M. Mladjenovic , W. C. W. Chan , Nat. Rev. Bioeng. 2024, 2, 201. 10.1038/s41563-023-01630-0 37592029

[229] M. Han , Z. Chen , S. Zhou , Z. Liao , X. Yi , Y. He , Y. Zhong , K. W. Leong , Nano Res. 2025, 18, 94907080.

[230] M. Ma , Y. Zhang , K. Pu , W. Tang , Chem. Soc. Rev. 2024, 54, 653. 10.1039/d4cs00679h 39620588

[231] L. Wu , Y. Jin , X. Zhao , K. Tang , Y. Zhao , L. Tong , X. Yu , K. Xiong , C. Luo , J. Zhu , F. Wang , Z. Zeng , D. Pan , Cell Metab. 2023, 35, 1580. 37506695 10.1016/j.cmet.2023.07.001

[232] C.‐H. Tsai , Y.‐M. Chuang , X. Li , Y.‐R. Yu , S.‐F. Tzeng , S. T. Teoh , K. E. Lindblad , M. D. Matteo , W.‐C. Cheng , P.‐C. Hsueh , K.‐C. Kao , H. Imrichova , L. Duan , H. Gallart‐Ayala , P.‐W. Hsiao , M. Mazzone , J. Ivanesevic , X. Liu , K. E. de Visser , A. Lujambio , S. Y. Lunt , S. M. Kaech , P.‐C. Ho , Cell Metab. 2023, 35, 118. 36599297 10.1016/j.cmet.2022.12.003 PMC10375941

[233] M. Mueckler , B. Thorens , Mol. Asp. Med. 2013, 34, 121. 10.1016/j.mam.2012.07.001 PMC4104978 23506862

[234] L. Szablewski , Acta (BBA) – Rev. Cancer 2013, 1835, 164. 10.1016/j.bbcan.2012.12.004 23266512

[235] F. R. R. Ayala , R. M. Rocha , K. C. Carvalho , A. L. Carvalho , I. W. D. Cunha , S. V. Lourenço , F. A. Soares , Molecules 2010, 15, 2374. 20428049 10.3390/molecules15042374 PMC6257354

[236] O. A. Ojelabi , K. P. Lloyd , A. H. Simon , J. K. D. Zutter , A. Carruthers , J. Biol. Chem. 2016, 291, 26762. 27836974 10.1074/jbc.M116.759175 PMC5207184

[237] M. Hayashi , K. Nakamura , S. Harada , M. Tanaka , A. Kobayashi , H. Saito , T. Tsuji , D. Yamamoto , H. Moriyama , J. Kinoshita , N. Inaki , BMC Cancer 2025, 25, 716. 40247224 10.1186/s12885-025-14141-9 PMC12004878

[238] K. Olszewski , A. Barsotti , X.‐J. Feng , M. Momcilovic , K. G. Liu , J.‐I. Kim , K. Morris , C. Lamarque , J. Gaffney , X. Yu , J. P. Patel , J. D. Rabinowitz , D. B. Shackelford , M. V. Poyurovsky , Cell Chem. Biol. 2022, 29, 423. 34715056 10.1016/j.chembiol.2021.10.007

[239] D. A. Chan , P. D. Sutphin , P. Nguyen , S. Turcotte , E. W. Lai , A. Banh , G. E. Reynolds , J.‐T. Chi , J. Wu , D. E. Solow‐Cordero , M. Bonnet , J. U. Flanagan , D. M. Bouley , E. E. Graves , W. A. Denny , M. P. Hay , A. J. Giaccia , Sci. Transl. Med. 2011, 3, 94ra70. 10.1126/scitranslmed.3002394 PMC3683134 21813754

[240] K. Tilekar , N. Upadhyay , C. V. Iancu , V. Pokrovsky , J. Choe , C. S. Ramaa , Biochim. Biophys. Acta (BBA) – Rev. Cancer 2020, 1874, 188457. 10.1016/j.bbcan.2020.188457 PMC7704680 33096154

[241] J. Yang , T. Su , Q. Wang , R. Shi , J. Ding , X. Chen , Adv. Mater. 2025, 37, 2419033. 10.1002/adma.202419033 39950419

[242] B. Kang , H. Wang , H. Jing , Y. Dou , S. Krizkova , Z. Heger , V. Adam , N. Li , J. Controlled Release 2024, 371, 338. 10.1016/j.jconrel.2024.05.025 38789089

[243] C.‐F. Xu , Y. Liu , S. Shen , Y.‐H. Zhu , J. Wang , Biomaterials 2015, 51, 1. 25770992 10.1016/j.biomaterials.2015.01.068

[244] S. Wu , K. Zhang , Y. Liang , Y. Wei , J. An , Y. Wang , J. Yang , H. Zhang , Z. Zhang , J. Liu , J. Shi , Adv. Sci. 2022, 9, 2103534. 10.1002/advs.202103534 PMC8895132 34913610

[245] A. Boudreau , H. E. Purkey , A. Hitz , K. Robarge , D. Peterson , S. Labadie , M. Kwong , R. Hong , M. Gao , C. D. Nagro , R. Pusapati , S. Ma , L. Salphati , J. Pang , A. Zhou , T. Lai , Y. Li , Z. Chen , B. Wei , I. Yen , S. Sideris , M. McCleland , R. Firestein , L. Corson , A. Vanderbilt , S. Williams , A. Daemen , M. Belvin , C. Eigenbrot , P. K. Jackson , et al., Nat. Chem. Biol. 2016, 12, 779. 27479743 10.1038/nchembio.2143

[246] N. Sun , M. Kabir , Y. Lee , L. Xie , X. Hu , J. Velez , X. Chen , H. U. Kaniskan , J. Jin , J. Med. Chem. 2023, 66, 596. 36538511 10.1021/acs.jmedchem.2c01505 PMC9969998

[247] A. Le , C. R. Cooper , A. M. Gouw , R. Dinavahi , A. Maitra , L. M. Deck , R. E. Royer , D. L. V. Jagt , G. L. Semenza , C. V. Dang , Proc. Natl. Acad. Sci. USA 2010, 107, 2037. 20133848 10.1073/pnas.0914433107 PMC2836706

[248] G. Bononi , V. D. Bussolo , T. Tuccinardi , F. Minutolo , C. Granchi , Expert Opin. Ther. Pat. 2024, 34, 1121. 39358962 10.1080/13543776.2024.2412575

[249] J. H. Han , E.‐J. Lee , W. Park , K.‐T. Ha , H.‐S. Chung , Front. Pharmacol. 2023, 14, 1275000. 37915411 10.3389/fphar.2023.1275000 PMC10616500

[250] Y.‐X. Zhang , Y.‐Y. Zhao , J. Shen , X. Sun , Y. Liu , H. Liu , Y. Wang , J. Wang , Nano Lett. 2019, 19, 2774. 30943039 10.1021/acs.nanolett.8b04296

[251] Q. Cheng , X. Shi , Q. Li , L. Wang , Z. Wang , Adv. Sci. 2024, 11, 2305662. 10.1002/advs.202305662 PMC10797484 37941489

[252] X. Han , X. Sheng , H. M. Jones , A. L. Jackson , J. Kilgore , J. E. Stine , M. N. Schointuch , C. Zhou , V. L. Bae‐Jump , J. Hematol. Oncol. 2015, 8, 2. 25631326 10.1186/s13045-014-0097-x PMC4316809

[253] X. Wu , S. Zhang , M. Feng , H. Sun , X. Lan , W. Liang , C. Liu , Y. Li , Adv. Healthcare Mater. 2025, 14, 2403380. 10.1002/adhm.202403380 39686828

[254] M. Kopp , S. Kollenda , M. Epple , Acc. Chem. Res. 2017, 50, 1383. 28480714 10.1021/acs.accounts.7b00051

[255] D. Jiang , D. Ni , Z. T. Rosenkrans , P. Huang , X. Yan , W. Cai , Chem. Soc. Rev. 2019, 48, 3683. 31119258 10.1039/c8cs00718g PMC6696937

[256] A. Reddy , S. Winther , N. Tran , H. Xiao , J. Jakob , R. Garrity , A. Smith , M. Ordonez , D. Laznik‐Bogoslavski , J. D. Rothstein , E. L. Mills , E. T. Chouchani , Nat. Metab. 2024, 6, 567. 38378996 10.1038/s42255-024-00981-5 PMC12540966

[257] R. L. Floch , J. Chiche , I. Marchiq , T. Naiken , T. Naïken , K. Ilc , K. Ilk , C. M. Murray , S. E. Critchlow , D. Roux , M.‐P. Simon , J. Pouysségur , Proc. Natl. Acad. Sci. USA 2011, 108, 16663. 21930917 10.1073/pnas.1106123108 PMC3189052

[258] J. R. Doherty , C. Yang , K. E. N. Scott , M. D. Cameron , M. Fallahi , W. Li , M. A. Hall , A. L. Amelio , J. K. Mishra , F. Li , M. Tortosa , H. M. Genau , R. J. Rounbehler , Y. Lu , C. V. Dang , K. G. Kumar , A. A. Butler , T. D. Bannister , A. T. Hooper , K. Unsal‐Kacmaz , W. R. Roush , J. L. Cleveland , Cancer Res. 2014, 74, 908. 24285728 10.1158/0008-5472.CAN-13-2034 PMC3946415

[259] R. Polański , C. L. Hodgkinson , A. Fusi , D. Nonaka , L. Priest , P. Kelly , F. Trapani , P. W. Bishop , A. White , S. E. Critchlow , P. D. Smith , F. Blackhall , C. Dive , C. J. Morrow , Clin. Cancer Res. 2014, 20, 926. 24277449 10.1158/1078-0432.CCR-13-2270 PMC3929348

[260] R. A. Noble , N. Bell , H. Blair , A. Sikka , H. Thomas , N. Phillips , S. Nakjang , S. Miwa , R. Crossland , V. Rand , D. Televantou , A. Long , H. C. Keun , C. M. Bacon , S. Bomken , S. E. Critchlow , S. R. Wedge , Haematologica 2017, 102, 1247. 28385782 10.3324/haematol.2016.163030 PMC5566036

[261] S. Halford , G. J. Veal , S. R. Wedge , G. S. Payne , C. M. Bacon , P. Sloan , I. Dragoni , K. Heinzmann , S. Potter , B. M. Salisbury , M. Chénard‐Poirier , A. Greystoke , E. C. Howell , W. A. Innes , K. Morris , C. Plummer , M. Rata , G. Petrides , H. C. Keun , U. Banerji , R. Plummer , Clin. Cancer Res. 2023, 29, 1429. 36652553 10.1158/1078-0432.CCR-22-2263 PMC7614436

[262] S. E. R. Halford , P. Jones , S. Wedge , S. Hirschberg , S. Katugampola , G. Veal , G. Payne , C. Bacon , S. Potter , M. Griffin , M. Chenard‐Poirier , G. Petrides , G. Holder , H. C. Keun , U. Banerji , E. R. Plummer , J. Clin. Oncol. 2017, 35, 2516.

[263] M. Guo , Y. Gou , X. Dong , J. Zhong , A. Li , A. Hao , T.‐C. He , J. Fan , Genes Dis 2024, 11, 101169. 38434753 10.1016/j.gendis.2023.101169 PMC10909599

[264] N. Babl , S.‐M. Decking , F. Voll , M. Althammer , A. Sala‐Hojman , R. Ferretti , C. Korf , C. Schmidl , L. Schmidleithner , B. Nerb , C. Matos , G. E. Koehl , P. Siska , C. Bruss , F. Kellermeier , K. Dettmer , P. J. Oefner , M. Wichland , I. Ugele , C. Bohr , W. Herr , S. Ramaswamy , T. Heinrich , C. Herhaus , M. Kreutz , K. Renner , J. Immunother. Cancer 2023, 11, 007349. 10.1136/jitc-2023-007349 PMC10603342 37880183

[265] Y. Qian , A. Galan‐Cobo , I. Guijarro , M. Dang , D. Molkentine , A. Poteete , F. Zhang , Q. Wang , J. Wang , E. Parra , A. Panda , J. Fang , F. Skoulidis , I. I. Wistuba , S. Verma , T. Merghoub , J. D. Wolchok , K.‐K. Wong , R. J. DeBerardinis , J. D. Minna , N. I. Vokes , C. B. Meador , J. F. Gainor , L. Wang , A. Reuben , J. V. Heymach , Cancer Cell 2023, 41, 1363. 37327788 10.1016/j.ccell.2023.05.015 PMC11161201

[266] V. L. Payen , E. Mina , V. F. V. Hée , P. E. Porporato , P. Sonveaux , Mol. Metab. 2020, 33, 48. 31395464 10.1016/j.molmet.2019.07.006 PMC7056923

[267] M. Beloueche‐Babari , S. Wantuch , T. C. Galobart , M. Koniordou , H. G. Parkes , V. Arunan , Y.‐L. Chung , T. R. Eykyn , P. D. Smith , M. O. Leach , Cancer Res. 2017, 77, 5913. 28923861 10.1158/0008-5472.CAN-16-2686 PMC5669455

[268] E. Lopez , R. Karattil , F. Nannini , G. W.‐K. Cheung , L. Denzler , F. Galvez‐Cancino , S. Quezada , M. A. Pule , J. Immunother. Cancer 2023, 11, 006287. 10.1136/jitc-2022-006287 PMC10314680 37399358

[269] J. Chen , Y. Zhu , C. Wu , J. Shi , Chem. Soc. Rev. 2023, 52, 973. 36597879 10.1039/d2cs00479h

[270] A. A. H. Abdellatif , A. Bouazzaoui , H. M. Tawfeek , M. A. Younis , Colloids Surf. B: Biointerfaces 2024, 238, 113930. 38692174 10.1016/j.colsurfb.2024.113930

[271] Y. Gao , A. Li , Y. Li , H. Guo , L. He , K. Li , D. Shcharbin , X. Shi , M. Shen , Biomacromolecules 2024, 25, 7995. 39570391 10.1021/acs.biomac.4c01249

[272] T. M. Grzywa , A. Sosnowska , P. Matryba , Z. Rydzynska , M. Jasinski , D. Nowis , J. Golab , Front. Immunol. 2020, 11, 938. 32499785 10.3389/fimmu.2020.00938 PMC7242730

[273] X. Zhang , X. Liu , W. Zhou , Q. Du , M. Yang , Y. Ding , R. Hu , Cell. Mol. Gastroenterol. Hepatol. 2021, 12, 1179. 34087454 10.1016/j.jcmgh.2021.05.018 PMC8445903

[274] G. C. Prendergast , W. P. Malachowski , J. B. DuHadaway , A. J. Muller , Cancer Res. 2017, 77, 6795. 29247038 10.1158/0008-5472.CAN-17-2285 PMC6021761

[275] W. Wang , W. Zou , A. Acids , Mol. Cell 2020, 80, 384. 32997964 10.1016/j.molcel.2020.09.006 PMC7655528

[276] C. Han , M. Ge , P.‐C. Ho , L. Zhang , Cancer Immunol. Res. 2021, 9, 1373. 34716193 10.1158/2326-6066.CIR-21-0459

[277] F. Castellano , V. Molinier‐Frenkel , Front. Cell Dev. Biol. 2020, 8, 613416. 33392202 10.3389/fcell.2020.613416 PMC7773816

[278] D. Nan , W. Yao , L. Huang , R. Liu , X. Chen , W. Xia , H. Sheng , H. Zhang , X. Liang , Y. Lu , Cell Commun. Signal. 2025, 23, 45. 39856712 10.1186/s12964-024-02018-6 PMC11760113

[279] Q. Yu , H. Tu , X. Yin , C. Peng , C. Dou , W. Yang , W. Wu , X. Guan , J. Li , H. Yan , Y. Zang , H. Jiang , Q. Xia , Front. Immunol. 2022, 13, 880262. 35663990 10.3389/fimmu.2022.880262 PMC9160195

[280] E. Martinenaite , S. M. Ahmad , S. K. Bendtsen , M. A. Jørgensen , S. E. Weis‐Banke , I. M. Svane , M. H. Andersen , Cancer Immunol., Immunother 2019, 68, 1901. 31690955 10.1007/s00262-019-02425-6 PMC11028269

[281] A. Werner , M. Koschke , N. Leuchtner , C. Luckner‐Minden , A. Habermeier , J. Rupp , C. Heinrich , R. Conradi , E. I. Closs , M. Munder , Front. Immunol. 2017, 8, 864. 28791021 10.3389/fimmu.2017.00864 PMC5523021

[282] D. M. Hardbower , M. Asim , P. B. Luis , K. Singh , D. P. Barry , C. Yang , M. A. Steeves , J. L. Cleveland , C. Schneider , M. B. Piazuelo , A. P. Gobert , K. T. Wilson , Proc. Natl. Acad. Sci. USA 2017, 114, E751. 28096401 10.1073/pnas.1614958114 PMC5293075

[283] C. Ye , Z. Geng , D. Dominguez , S. Chen , J. Fan , L. Qin , A. Long , Y. Zhang , T. M. Kuzel , B. Zhang , J. Immunol. 2016, 196, 915. 26663722 10.4049/jimmunol.1500729 PMC4707077

[284] J. E. Cheong , L. Sun , Trends Pharmacol. Sci. 2018, 39, 307. 29254698 10.1016/j.tips.2017.11.007

[285] M. Platten , E. A. A. Nollen , U. F. Röhrig , F. Fallarino , C. A. Opitz , Nat. Rev. Drug Discovery 2019, 18, 379. 30760888 10.1038/s41573-019-0016-5

[286] K. Hayashi , P. Jutabha , H. Endou , H. Sagara , N. Anzai , J. Immunol. 2013, 191, 4080. 24038088 10.4049/jimmunol.1300923

[287] S. Jakobsen , C. U. Nielsen , Pharmaceutics 2024, 16, 197. 38399253 10.3390/pharmaceutics16020197 PMC10893028

[288] C. S. Kim , S.‐H. Cho , H. S. Chun , S.‐Y. Lee , H. Endou , Y. Kanai , D. K. Kim , Biol. Pharm. Bull. 2008, 31, 1096. 18520037 10.1248/bpb.31.1096

[289] N. Muhammad , H. M. Lee , J. Kim , Cells 2020, 9, 1904.32824193

[290] S. Bröer , Int. J. Mol. Sci. 2020, 21, 6156.32859034

[291] Y. Hara , Y. Minami , S. Yoshimoto , N. Hayashi , A. Yamasaki , S. Ueda , K. Masuko , T. Masuko , Cancer Med. 2020, 9, 302. 31709772 10.1002/cam4.2689 PMC6943164

[292] K. Hushmandi , B. Einollahi , S. H. Saadat , E. H. C. Lee , M. R. Farani , E. Okina , Y. S. Huh , N. Nabavi , S. Salimimoghadam , A. P. Kumar , Mol. Metab. 2024, 84, 101952. 38705513 10.1016/j.molmet.2024.101952 PMC11112377

[293] L. Yang , A. Achreja , T.‐L. Yeung , L. S. Mangala , D. Jiang , C. Han , J. Baddour , J. C. Marini , J. Ni , R. Nakahara , S. Wahlig , L. Chiba , S. H. Kim , J. Morse , S. Pradeep , A. S. Nagaraja , M. Haemmerle , N. Kyunghee , M. Derichsweiler , T. Plackemeier , I. Mercado‐Uribe , G. Lopez‐Berestein , T. Moss , P. T. Ram , J. Liu , X. Lu , S. C. Mok , A. K. Sood , D. Nagrath , Cell Metab. 2016, 24, 685. 27829138 10.1016/j.cmet.2016.10.011 PMC7329194

[294] Z. Wang , F. Liu , N. Fan , C. Zhou , D. Li , T. Macvicar , Q. Dong , C. J. Bruns , Y. Zhao , Front. Oncol. 2020, 10, 589508. 33194749 10.3389/fonc.2020.589508 PMC7649373

[295] L. Xu , D. Zhou , F. Li , L. Ji , Cancer Res. 2020, 9, 4906. 10.21037/tcr-20-2246 PMC8798858 35117852

[296] Y. Xiang , Z. E. Stine , J. Xia , Y. Lu , R. S. O'Connor , B. J. Altman , A. L. Hsieh , A. M. Gouw , A. G. Thomas , P. Gao , L. Sun , L. Song , B. Yan , B. S. Slusher , J. Zhuo , L. L. Ooi , C. G. L. Lee , A. Mancuso , A. S. McCallion , A. Le , M. C. Milone , S. Rayport , D. W. Felsher , C. V. Dang , J. Clin. Investig. 2015, 125, 2293. 25915584 10.1172/JCI75836 PMC4497742

[297] L. Tenora , J. Alt , R. P. Dash , A. J. Gadiano , K. Novotná , V. Veeravalli , J. Lam , Q. R. Kirkpatrick , K. M. Lemberg , P. Majer , R. Rais , B. S. Slusher , J. Med. Chem. 2019, 62, 3524. 30892035 10.1021/acs.jmedchem.8b02009 PMC8025739

[298] C.‐H. Lee , R. Motzer , H. Emamekhoo , M. Matrana , I. Percent , J. J. Hsieh , A. Hussain , U. Vaishampayan , S. Liu , S. McCune , V. Patel , M. Shaheen , J. Bendell , A. C. Fan , B. A. Gartrell , O. B. Goodman , P. G. Nikolinakos , A. R. Kalebasty , Y. Zakharia , Z. Zhang , H. Parmar , L. Akella , K. Orford , N. M. Tannir , Clin. Cancer Res. 2022, 28, 3248. 35576438 10.1158/1078-0432.CCR-22-0061 PMC10202043

[299] F. Meric‐Bernstam , N. M. Tannir , O. Iliopoulos , R. J. Lee , M. L. Telli , A. C. Fan , A. DeMichele , N. B. Haas , M. R. Patel , J. J. Harding , M. H. Voss , T. K. Owonikoko , B. Carthon , R. Srinivasan , J. C. Bendell , Y. Jenkins , S. H. Whiting , K. Orford , M. K. Bennett , T. M. Bauer , Clin. Cancer Res. 2022, 28, 1540. 35140121 10.1158/1078-0432.CCR-21-2972 PMC9164172

[300] N. M. Tannir , N. Agarwal , C. Porta , N. J. Lawrence , R. Motzer , B. McGregor , R. J. Lee , R. K. Jain , N. Davis , L. J. Appleman , O. Goodman , W. M. Stadler , S. Gandhi , D. M. Geynisman , R. Iacovelli , B. Mellado , J. M. S. Sánchez , R. Figlin , T. Powles , L. Akella , K. Orford , B. Escudier , JAMA Oncol 2022, 8, 1411. 36048457 10.1001/jamaoncol.2022.3511 PMC9437824

[301] B. Li , Y. Cao , G. Meng , L. Qian , T. Xu , C. Yan , O. Luo , S. Wang , J. Wei , Y. Ding , D. Yu , EBioMedicine 2019, 39, 239. 30555042 10.1016/j.ebiom.2018.11.063 PMC6355660

[302] S. Akar , H. D. Altuntaþ , Z. Hamurcu , ERCÝYES Méd. J. 2022, 0.

[303] J. Shi , W. Han , J. Wang , X. Kong , Adv. Mater. 2025, 37, 2415550. 10.1002/adma.202415550 39895165

[304] A. Sosnowska , J. Chlebowska‐Tuz , P. Matryba , Z. Pilch , A. Greig , A. Wolny , T. M. Grzywa , Z. Rydzynska , O. Sokolowska , T. P. Rygiel , M. Grzybowski , P. Stanczak , R. Blaszczyk , D. Nowis , J. Golab , OncoImmunology 2021, 10, 1956143. 34367736 10.1080/2162402X.2021.1956143 PMC8312619

[305] V. Vonwirth , Y. Bülbül , A. Werner , H. Echchannaoui , J. Windschmitt , A. Habermeier , S. Ioannidis , N. Shin , R. Conradi , M. Bros , S. Tenzer , M. Theobald , E. I. Closs , M. Munder , Front. Immunol. 2021, 11, 617699. 33717053 10.3389/fimmu.2020.617699 PMC7952869

[306] S. M. Steggerda , M. K. Bennett , J. Chen , E. Emberley , T. Huang , J. R. Janes , W. Li , A. L. MacKinnon , A. Makkouk , G. Marguier , P. J. Murray , S. Neou , A. Pan , F. Parlati , M. L. M. Rodriguez , L.‐A. V. de Velde , T. Wang , M. Works , J. Zhang , W. Zhang , Cancer 2017, 5, 101. 10.1186/s40425-017-0308-4 PMC5735564 29254508

[307] A. Naing , K. P. Papadopoulos , M. J. Pishvaian , O. Rahma , G. J. Hanna , E. Garralda , O. Saavedra , S. Gogov , H. Kallender , L. Cheng , M. Smith , X. Chen , E. Kuriakose , T. Bauer , BMJ Oncol 2024, 3, 000249. 10.1136/bmjonc-2023-000249 PMC11235002 39886141

[308] R. S. Hesterberg , J. L. Cleveland , P. K. Epling‐Burnette , Méd. Sci. 2018, 6, 22. 10.3390/medsci6010022 PMC5872179 29517999

[309] C. E. Holbert , M. T. Cullen , R. A. Casero , T. M. Stewart , Nat. Rev. Cancer 2022, 22, 467. 35477776 10.1038/s41568-022-00473-2 PMC9339478

[310] R. A. Casero , T. M. Stewart , A. E. Pegg , Nat. Rev. Cancer 2018, 18, 681. 30181570 10.1038/s41568-018-0050-3 PMC6487480

[311] B. Escriche‐Navarro , A. Escudero , E. Lucena‐Sánchez , F. Sancenón , A. García‐Fernández , R. Martínez‐Máñez , Adv. Sci. 2022, 9, 2200756. 10.1002/advs.202200756 PMC9475525 35866466

[312] A. Zhang , H. Wu , X. Chen , Z. Chen , Y. Pan , W. Qu , H. Hao , D. Chen , S. Xie , Sci. Adv. 2023, 9, adg9116. 10.1126/sciadv.adg9116 PMC10348676 37450586

[313] E. Markova , C. Wolowczyk , A. Mohamed , A. M. Sofias , M. Martin‐Armas , R. Sundset , J. Berndtsson , S. Hak , N. Škalko‐Basnet , Eur. J. Pharm. Sci. 2025, 204, 106959. 39521192 10.1016/j.ejps.2024.106959

[314] K. Tang , Y.‐H. Wu , Y. Song , B. Yu , J. Hematol. Oncol. 2021, 14, 68. 33883013 10.1186/s13045-021-01080-8 PMC8061021

[315] N. Kotecki , P. Vuagnat , B. H. O'Neil , S. Jalal , S. Rottey , H. Prenen , K. A. Benhadji , M. Xia , A. M. Szpurka , A. Saha , J. Wallin , S. Suriyapperuma , V. R. Galvao , S. Geeganage , T. N. Doman , L. Gandhi , X. Xu , J. Bendell , J. Immunother. 2021, 44, 264. 33928928 10.1097/CJI.0000000000000368

[316] B. W. Labadie , R. Bao , J. J. Luke , Clin. Cancer Res. 2018, 25, 2882. 10.1158/1078-0432.CCR-18-2882 PMC6397695 30377198

[317] I. Kang , G. Theodoropoulos , M. Wangpaichitr , Front. Oncol. 2025, 14, 1524651. 39911818 10.3389/fonc.2024.1524651 PMC11794083

[318] R. Nadal , B. P. Valderrama , J. Bellmunt , Nat. Rev. Clin. Oncol. 2024, 21, 8. 37945764 10.1038/s41571-023-00826-2

[319] J. W. Kjeldsen , C. L. Lorentzen , E. Martinenaite , E. Ellebaek , M. Donia , R. B. Holmstroem , T. W. Klausen , C. O. Madsen , S. M. Ahmed , S. E. Weis‐Banke , M. O. Holmström , H. W. Hendel , E. Ehrnrooth , M.‐B. Zocca , A. W. Pedersen , M. H. Andersen , I. M. Svane , Nat. Med. 2021, 27, 2212. 34887574 10.1038/s41591-021-01544-x PMC8904254

[320] C. L. Lorentzen , J. W. Kjeldsen , E. Ehrnrooth , M. H. Andersen , I. M. Svane , J. Immunother. Cancer 2023, 11, 006755. 10.1136/jitc-2023-006755 PMC10230976 37217243

[321] F. Peyraud , J.‐P. Guegan , D. Bodet , S. Cousin , A. Bessede , A. Italiano , Front. Immunol. 2022, 13, 807271. 35173722 10.3389/fimmu.2022.807271 PMC8841724

[322] L. Zhai , A. Bell , E. Ladomersky , K. L. Lauing , L. Bollu , B. Nguyen , M. Genet , M. Kim , P. Chen , X. Mi , J. D. Wu , M. J. Schipma , B. Wray , J. Griffiths , R. D. Unwin , S. J. Clark , R. Acharya , R. Bao , C. Horbinski , R. V. Lukas , G. E. Schiltz , Clin. Cancer Res. 2021, 27, 6514. 34479957 10.1158/1078-0432.CCR-21-1392 PMC8639612

[323] R. Endo , T. Nakamura , K. Kawakami , Y. Sato , H. Harashima , Sci. Rep. 2019, 9, 11335. 31383907 10.1038/s41598-019-47799-w PMC6683295

[324] Y. Zhou , Q. Tao , C. Luo , J. Chen , G. Chen , J. Sun , Cancer Sci. 2025, 70057. 10.1111/cas.70057 PMC12127106 40103010

[325] Y. Guo , Y. Liu , W. Wu , D. Ling , Q. Zhang , P. Zhao , X. Hu , Biomaterials 2021, 276, 121018. 34284200 10.1016/j.biomaterials.2021.121018

[326] H. Wu , X. Sun , K. Li , J. Li , H. Jiang , D. Yan , Y. Lin , Y. Ding , Y. Lu , X. Zhu , X. Chen , X. Li , G. Liang , H. Xu , Adv. Sci. 2025, 12, 2409790. 10.1002/advs.202409790 PMC11831488 39716923

[327] M. Han , S. Zhou , Z. Liao , C. Zishan , X. Yi , C. Wu , D. Zhang , Y. He , K. W. Leong , Y. Zhong , Biomaterials 2025, 315, 122934. 39509856 10.1016/j.biomaterials.2024.122934

[328] A. Sadik , L. F. S. Patterson , S. Öztürk , S. R. Mohapatra , V. Panitz , P. F. Secker , P. Pfänder , S. Loth , H. Salem , M. T. Prentzell , B. Berdel , M. Iskar , E. Faessler , F. Reuter , I. Kirst , V. Kalter , K. I. Foerster , E. Jäger , C. R. Guevara , M. Sobeh , T. Hielscher , G. Poschet , A. Reinhardt , J. C. Hassel , M. Zapatka , U. Hahn , A. von Deimling , C. Hopf , R. Schlichting , B. I. Escher , et al., Cell 2020, 182, 1252. 32818467 10.1016/j.cell.2020.07.038

[329] X. Luo , C. Cheng , Z. Tan , N. Li , M. Tang , L. Yang , Y. Cao , Mol. Cancer 2017, 16, 76. 28399876 10.1186/s12943-017-0646-3 PMC5387196

[330] S. A. Lim , W. Su , N. M. Chapman , H. Chi , Nat. Chem. Biol. 2022, 18, 470. 35484263 10.1038/s41589-022-01017-3 PMC11103273

[331] E. Cuyàs , S. Pedarra , S. Verdura , M. A. Pardo , R. E. Garcia , E. Serrano‐Hervás , À. Llop‐Hernández , E. Teixidor , J. Bosch‐Barrera , E. López‐Bonet , B. Martin‐Castillo , R. Lupu , M. A. Pujana , J. Sardanyès , T. Alarcón , J. A. Menendez , Cell Death Discov. 2024, 10, 417. 39349429 10.1038/s41420-024-02184-z PMC11442875

[332] J. Hou , Y. Wang , L. Shi , Y. Chen , C. Xu , A. Saeedi , K. Pan , R. Bohat , N. A. Egan , J. A. McKenzie , R. M. Mbofung , L. J. Williams , Z. Yang , M. Sun , X. Liang , J. R. Ahnert , N. Varadarajan , C. Yee , Y. Chen , P. Hwu , W. Peng , J. Immunother. Cancer 2021, 9, 001819. 10.1136/jitc-2020-001819 PMC7887353 33589527

[333] S. J. Kridel , F. Axelrod , N. Rozenkrantz , J. W. Smith , Cancer Res. 2004, 64, 2070. 15026345 10.1158/0008-5472.can-03-3645

[334] C. Rae , G. I. Fragkoulis , A. J. Chalmers , Adv. Radiat. Oncol. 2020, 5, 994. 33083663 10.1016/j.adro.2020.06.022 PMC7557210

[335] G. Falchook , J. Infante , H.‐T. Arkenau , M. R. Patel , E. Dean , E. Borazanci , A. Brenner , N. Cook , J. Lopez , S. Pant , A. Frankel , P. Schmid , K. Moore , W. McCulloch , K. Grimmer , M. O'Farrell , G. Kemble , H. Burris , EClinicalMedicine 2021, 34, 100797. 33870151 10.1016/j.eclinm.2021.100797 PMC8040281

[336] S. D. Sardesai , A. Thomas , C. Gallagher , F. Lynce , Y. L. Ottaviano , T. J. Ballinger , B. P. Schneider , A. M. Storniolo , A. Bauchle , S. K. Althouse , S. M. Perkins , A. R. Masters , R. E. Stratford , Z. Dong , J.‐Y. Liu , J.‐T. Zhang , K. D. Miller , Clin. Cancer Res. 2021, 27, 5810. 34400413 10.1158/1078-0432.CCR-21-0493

[337] J. Huang , W. Y. Tsang , X.‐N. Fang , Y. Zhang , J. Luo , L.‐Q. Gong , B.‐F. Zhang , C. N. Wong , Z.‐H. Li , B.‐L. Liu , J.‐L. Huang , Y.‐M. Yang , S. Liu , L.‐X. Ban , Y. H. Chan , X.‐Y. Guan , Cancer Res. 2024, 84, 855. 38486485 10.1158/0008-5472.CAN-23-0966

[338] S. Kant , P. Kesarwani , A. Prabhu , S. F. Graham , K. L. Buelow , I. Nakano , P. Chinnaiyan , Cell Death Dis. 2020, 11, 253. 32312953 10.1038/s41419-020-2449-5 PMC7170895

[339] Q. Qu , F. Zeng , X. Liu , Q. J. Wang , F. Deng , Cell Death Dis. 2016, 7, 2226. 10.1038/cddis.2016.132 PMC4917665 27195673

[340] Y. Ma , S. M. Temkin , A. M. Hawkridge , C. Guo , W. Wang , X.‐Y. Wang , X. Fang , Cancer Lett. 2018, 435, 92. 30102953 10.1016/j.canlet.2018.08.006 PMC6240910

[341] Z. Wang , Y. Wang , Z. Li , W. Xue , S. Hu , X. Kong , Front. Pharmacol. 2023, 14, 1274335. 37841917 10.3389/fphar.2023.1274335 PMC10571713

[342] F. Pagliari , J. Jansen , J. Knoll , R. Hanley , J. Seco , L. Tirinato , Cell Div 2024, 19, 14. 38643120 10.1186/s13008-024-00116-y PMC11031927

[343] Y. Wang , H. Pan , D. chen , D. Guo , X. Wang , J. Funct. Foods 2021, 83, 104570.

[344] Z. Li , H. Liu , X. Luo , Am. J. Cancer Res. 2020, 10, 4112. 33414989 PMC7783747

[345] N. Ricco , S. J. Kron , Cancers 2023, 15, 3948. 37568764 10.3390/cancers15153948 PMC10417177

[346] A. F. Hassanabad , Transl. Lung Cancer Res. 2019, 8, 692. 31737505 10.21037/tlcr.2019.09.08 PMC6835101

[347] Y. Zhou , J. Tashiro , S. Kamatani , N. Irie , A. Suzuki , T. Ishikawa , K. Warita , Z. N. Oltvai , T. Warita , Biophys. Res. Commun. 2023, 677, 13. 10.1016/j.bbrc.2023.07.056 37541087

[348] W. Mao , Y. Cai , D. Chen , G. Jiang , Y. Xu , R. Chen , F. Wang , X. Wang , M. Zheng , X. Zhao , J. Mei , JCI Insight 2022, 7, 161940. 10.1172/jci.insight.161940 PMC9675559 35943796

[349] M. Takai , S. Mori , K. Honoki , T. Tsujiuchi , J. Bioenerg. Biomembr. 2024, 56, 475. 38886303 10.1007/s10863-024-10028-9

[350] Y. Ping , Q. Fan , Y. Zhang , Cancer 2025, 13, 010824. 10.1136/jitc-2024-010824 PMC11795363 39904563

[351] B. Tu , Y. Gao , F. Sun , M. Shi , Y. Huang , Front. Pharmacol. 2022, 13, 840440. 35392570 10.3389/fphar.2022.840440 PMC8980325

[352] J. Wu , X. Zhang , D. Sun , X. Shi , J. Sun , C. Luo , Z. He , S. Zhang , Chem. Eng. J. 2024, 495, 153366.

[353] D. Kim , Y. Wu , Q. Li , Y.‐K. Oh , Nano‐Micro Lett. 2021, 13, 31. 10.1007/s40820-020-00555-6 PMC8006499 34138236

[354] G. Wang , D. Wang , L. Xia , J. Lian , Q. Zhang , D. Shen , Z. Wang , Y. Dai , ACS Appl. Mater. Interfaces 2025, 17, 7478. 39871538 10.1021/acsami.4c21028 PMC11803545

[355] X. Cheng , J. Xu , Y. Cui , J. Liu , Y. Chen , C. He , L. Cui , Y. Liu , B. Song , C. Gong , P. Mi , ACS Nano 2025, 19, 7213. 39928515 10.1021/acsnano.4c16981

[356] A. Khan , A. N. Aljarbou , Y. H. Aldebasi , K. S. Allemailem , M. A. Alsahli , S. Khan , A. M. Alruwetei , M. A. Khan , Int. J. Nanomed. 2020, 15, 5575. 10.2147/IJN.S256022 PMC7415462 32801705

[357] J. Zhang , Y. Yin , J. Zhang , J. Zhang , W. Su , H. Ma , F. Jia , G. Zhao , H. Wang , Nano Lett. 2022, 22, 2514. 35285648 10.1021/acs.nanolett.2c00356

[358] Y. Zhang , Y. Ren , H. Xu , L. Li , F. Qian , L. Wang , A. Quan , H. Ma , H. Liu , R. Yu , ACS Appl. Mater. Interfaces 2023, 15, 10356. 36787514 10.1021/acsami.2c19285

[359] M. Zhang , X. Yao , J. Xu , J. Song , S. Mai , W. Zhu , Y. Zhang , L. Zhu , W. Yang , Int. J. Pharm. 2024, 655, 124032. 38521374 10.1016/j.ijpharm.2024.124032

[360] J. Zhang , Y. Zhou , J. Guo , M. Yan , C. Liu , B. Du , ACS Appl. Mater. Interfaces 2025, 17, 6689. 39813326 10.1021/acsami.4c17858

[361] K. Kuche , V. Yadav , M. Dharshini , R. Ghadi , D. Chaudhari , T. Date , S. Jain , Int. J. Biol. Macromol. 2023, 253, 127254. 37813219 10.1016/j.ijbiomac.2023.127254

[362] J. Yang , Z. Jia , J. Zhang , X. Pan , Y. Wei , S. Ma , N. Yang , Z. Liu , Q. Shen , Adv. Healthcare Mater. 2022, 11, 2102799. 10.1002/adhm.202102799 35395704

[363] A. Göbel , S. Pählig , A. Motz , D. Breining , S. Traikov , L. C. Hofbauer , T. D. Rachner , Biochem. Biophys. Res. Commun. 2024, 710, 149841. 38588613 10.1016/j.bbrc.2024.149841

[364] S. Cao , P. E. Saw , Q. Shen , R. Li , Y. Liu , X. Xu , Biomaterials 2022, 280, 121264. 34823884 10.1016/j.biomaterials.2021.121264

[365] D. Teng , K. D. Swanson , R. Wang , A. Zhuang , H. Wu , Z. Niu , L. Cai , F. R. Avritt , L. Gu , J. M. Asara , Y. Zhang , B. Zheng , Nat. Commun. 2025, 16, 3867. 40274823 10.1038/s41467-025-59307-y PMC12022163

[366] M. G. Rose , M. P. Farrell , J. C. Schmitz , Clin. Color. Cancer 2002, 1, 220. 10.3816/CCC.2002.n.003 12450420

[367] Z. Zuo , Z. Zhou , Y. Chang , Y. Liu , Y. Shen , Q. Li , L. Zhang , Genes Dis 2024, 11, 218. 37588202 10.1016/j.gendis.2022.11.022 PMC10425756

[368] M. W. Musiałek , D. Rybaczek , Genes 2021, 12, 1096. 34356112 10.3390/genes12071096 PMC8304116

[369] A. A. Valencia‐Lazcano , D. Hassan , M. Pourmadadi , A. shamsabadipour , R. Behzadmehr , A. Rahdar , D. I. Medina , Eur. J. Med. Chem. 2023, 246, 114995. 36493619 10.1016/j.ejmech.2022.114995

[370] M. Ueno , H. Takabatake , A. Hata , T. Kayahara , Y. Morimoto , K. Notohara , M. Mizuno , Cancer Rep 2022, 5, 1624. 10.1002/cnr2.1624 PMC9458512 35575047

[371] B. Singh , V. N. Sarli , A. Lucci , Oncotarget 2021, 12, 626. 33868584 10.18632/oncotarget.27922 PMC8021029

[372] C. Mao , S. Yeh , J. Fu , M. Porosnicu , A. Thomas , G. L. Kucera , K. I. Votanopoulos , S. Tian , X. Ming , Sci. Transl. Med. 2022, 14, abh1261. 10.1126/scitranslmed.abh1261 PMC9499735 35675434

[373] A. Naaz , H. R. Turnquist , V. S. Gorantla , S. R. Little , Adv. Drug Delivery Rev. 2024, 213, 115429. 10.1016/j.addr.2024.115429 39142608

[374] P. Liu , J. Guo , Z. Xie , Y. Pan , B. Wei , Y. Peng , S. Hu , J. Ding , X. Chen , J. Su , H. Liu , W. Zhou , Adv. Sci. 2024, 2410545. 10.1002/advs.202410545 PMC11831434 39716993

[375] T.‐J. Zhou , M.‐M. Zhang , D.‐M. Liu , L.‐L. Huang , H.‐Q. Yu , Y. Wang , L. Xing , H.‐L. Jiang , Biomaterials 2024, 305, 122447. 38154441 10.1016/j.biomaterials.2023.122447

[376] X.‐H. Ren , L. Shi , Z.‐B. Ma , D.‐W. Wang , X.‐W. He , W.‐Y. Li , Y.‐K. Zhang , Chem. Eng. J. 2024, 481, 148739.

[377] M. Lv , B. Liu , Y. Duan , J. Lin , L. Dai , Y. Li , J. Yu , J. Liao , J. Zhang , Y. Duan , ACS Nano 2024, 18, 27487. 39329191 10.1021/acsnano.4c08055

[378] C.‐S. Yuan , Z. Teng , S. Yang , Z. He , L.‐Y. Meng , X.‐G. Chen , Y. Liu , J. Controlled Release 2022, 351, 255. 10.1016/j.jconrel.2022.09.029 36165836

[379] Y. Chen , Y. Song , C. Zhang , P. Jin , Y. Fu , G. Wang , L. Tang , J. Chen , X. Xu , P. Huang , J. Controlled Release 2025, 383, 113819. 10.1016/j.jconrel.2025.113819 40345625

[380] L. Wu , W. Xie , Y. Li , Q. Ni , P. Timashev , M. Lyu , L. Xia , Y. Zhang , L. Liu , Y. Yuan , X. Liang , Q. Zhang , Adv. Sci. 2022, 9, 2105376. 10.1002/advs.202105376 PMC9189650 35396800

[381] C. Zhang , J. Huang , Z. Zeng , S. He , P. Cheng , J. Li , K. Pu , Nat. Commun. 2022, 13, 3468. 35710545 10.1038/s41467-022-31044-6 PMC9203767

[382] W. Yu , J. Sun , X. Wang , S. Yu , M. Yan , F. Wang , X. Liu , Adv. Mater. 2022, 34, 2106967. 10.1002/adma.202106967 34910838

[383] L. Giuffrida , K. Sek , M. A. Henderson , J. Lai , A. X. Y. Chen , D. Meyran , K. L. Todd , E. V. Petley , S. Mardiana , C. Mølck , G. D. Stewart , B. J. Solomon , I. A. Parish , P. J. Neeson , S. J. Harrison , L. M. Kats , I. G. House , P. K. Darcy , P. A. Beavis , Nat. Commun. 2021, 12, 3236. 34050151 10.1038/s41467-021-23331-5 PMC8163771

[384] Z. Chen , X. Yi , Z. Liao , S. Zhou , M. Han , C. Wu , D. Zhang , Y. He , K. W. Leong , Y. Zhong , Cell Biomater 2025, 1, 100104. 10.1016/j.biomaterials.2024.122934 39509856

[385] H. Zhang , L. Yang , M. Han , Y. Han , Z. Jiang , Q. Zheng , J. Dong , T. Wang , Z. Li , ACS Nano 2024, 18, 23001. 39150454 10.1021/acsnano.4c04553

[386] F. Kang , M. Niu , Z. Zhou , M. Zhang , H. Xiong , F. Zeng , J. Wang , X. Chen , Adv. Healthcare Mater. 2024, 13, 2400908. 10.1002/adhm.202400908 38598819

[387] P. Ghosh , C. Vidal , S. Dey , L. Zhang , Int. J. Mol. Sci. 2020, 21, 3363. 32397535 10.3390/ijms21093363 PMC7247703

[388] J. S. Carew , P. Huang , Mol. Cancer 2002, 1, 9. 12513701 10.1186/1476-4598-1-9 PMC149412

[389] F. Bost , L. Kaminski , Am. J. Cancer Res. 2018, 9, 198. PMC6405967 30906622

[390] D. P. Boulton , M. C. Caino , Front. Cell Dev. Biol. 2022, 10, 849962. 35356277 10.3389/fcell.2022.849962 PMC8959575

[391] H. Chen , S. A. Detmer , A. J. Ewald , E. E. Griffin , S. E. Fraser , D. C. Chan , J. Cell Biol. 2003, 160, 189. 12527753 10.1083/jcb.200211046 PMC2172648

[392] S. S. Sabharwal , P. T. Schumacker , Nat. Rev. Cancer 2014, 14, 709. 25342630 10.1038/nrc3803 PMC4657553

[393] L. B. Sullivan , N. S. Chandel , Cancer Metab 2014, 2, 17. 25671107 10.1186/2049-3002-2-17 PMC4323058

[394] I. Alodhaibi , S. Ailawadhi , G. P. Burbano , P. J. O'Brien , F. K. Buadi , S. Hayman , S. K. Kumar , W. I. Gonsalves , Clin. Lymphoma Myeloma Leuk. 2024, 24, 298. 38220589 10.1016/j.clml.2024.01.002 PMC11045312

[395] C. Schlesser , T. Meul , G. Stathopoulos , S. Meiners , Biomolecules 2022, 12, 756. 35740881 10.3390/biom12060756 PMC9221333

[396] X. Long , M. Liu , Y. Nan , Q. Chen , Z. Xiao , Y. Xiang , X. Ying , J. Sun , Q. Huang , K. Ai , Adv. Mater. 2024, 36, 2308239. 10.1002/adma.202308239 38224339

[397] Z. Zhou , N. Jiang , J. Chen , C. Zheng , Y. Guo , R. Ye , R. Qi , J. Shen , J. Nanobiotechnol. 2021, 19, 375. 10.1186/s12951-021-01124-8 PMC8600872 34794446

[398] H. Lu , W. Tong , M. Jiang , H. Liu , C. Meng , K. Wang , X. Mu , ACS Nano 2024, 18, 21156. 39088743 10.1021/acsnano.4c04024

[399] A. Ashokan , S. Sarkar , M. Z. Kamran , B. Surnar , A. A. Kalathil , A. Spencer , S. Dhar , Proc. Natl. Acad. Sci. USA 2024, 121, 2318119121. 10.1073/pnas.2318119121 PMC11098113 38709930

[400] J. Fernandez‐Alarcon , M. A. Cladera , N. Rodriguez‐Camenforte , G. Sitia , M. Guerra‐Rebollo , S. Borros , C. Fornaguera , Biomaterials 2025, 318, 123164. 39923537 10.1016/j.biomaterials.2025.123164

[401] W. Xu , A. Suo , A. J. M. Aldai , Y. Wang , J. Fan , Y. Xia , J. Xu , Z. Chen , H. Zhao , M. Zhang , J. Qian , ACS Nano 2024, 18, 30053. 39412236 10.1021/acsnano.4c11455

[402] W. Wang , S.‐Y. Yao , J. Luo , C. Ding , Q. Huang , Y. Yang , Z. Shi , J. Lin , Y.‐C. Pan , X. Zeng , D.‐S. Guo , H. Chen , Nat. Commun. 2025, 16, 596. 39799105 10.1038/s41467-025-55905-y PMC11724902

[403] H. Wang , F. Zhang , H. Wen , W. Shi , Q. Huang , Y. Huang , J. Xie , P. Li , J. Chen , L. Qin , Y. Zhou , J. Nanobiotechnol. 2020, 18, 8. 10.1186/s12951-019-0562-3 PMC6950814 31918714

[404] Y. Tong , P. An , P. Tang , R. Mu , Y. Zeng , H. Sun , M. Zhao , Z. Lv , P. Wang , W. Han , C. Gui , X. Zhen , L. Han , Acta Pharm. Sin. B 2024, 14, 2716. 38828148 10.1016/j.apsb.2024.03.024 PMC11143535

[405] T. A. El‐Masry , M. M. F. El‐Nagar , G. A. Oriquat , B. S. Alotaibi , H. M. Saad , E. I. E. Zahaby , H. A. Ibrahim , Biomed. Pharmacother. 2024, 180, 117429. 39293373 10.1016/j.biopha.2024.117429

[406] Z. Qu , Y. Ren , H. Shen , H. Wang , L. Shi , D. Tong , Dev. Ther. 2021, 15, 3605. 10.2147/DDDT.S306684 PMC8384126 34447241

[407] G. Bebawy , P. Collier , P. M. Williams , J. C. Burley , D. Needham , Int. J. Pharm. 2025, 676, 125574. 40239877 10.1016/j.ijpharm.2025.125574

[408] Z. Yu , J. Guo , Y. Liu , M. Wang , Z. Liu , Y. Gao , L. Huang , J. Nanobiotechnol. 2022, 20, 9. 10.1186/s12951-021-01205-8 PMC8725360 34983554

[409] H. Tan , Z. Shen , X. Wang , S. Shu , J. Deng , L. Lu , Z. Fan , D. Hu , P. Cheng , X. Cao , Q. Huang , J. Controlled Release 2024, 375, 422. 10.1016/j.jconrel.2024.09.018 39278355

[410] T. Yang , Z. Liu , Z. Fu , X. Zhang , Y. Cao , Q. Liang , J. Miao , H. Yang , T. Zhang , J. Hei , W. Ni , Y. Liu , Asian J. Pharm. Sci. 2025, 20, 100970. 40213381 10.1016/j.ajps.2024.100970 PMC11985007

[411] B. Alkotub , L. Bauer , A. B. Dezfouli , K. Hachani , V. Ntziachristos , G. Multhoff , M. H. Kafshgari , Redox Biol. 2025, 79, 103452. 39667305 10.1016/j.redox.2024.103452 PMC11697781

[412] Y. Xie , J. Guo , J. Hu , Y. Li , Z. Zhang , Y. Zhu , F. Deng , J. Qi , Y. Zhou , W. Chen , Mater. Today Bio 2025, 32, 101703. 10.1016/j.mtbio.2025.101703 PMC11994397 40230646

[413] J. Ma , D. Guo , X. Ji , Y. Zhou , C. Liu , Q. Li , J. Zhang , C. Fan , H. Song , Adv. Mater. 2023, 35, 2211579. 10.1002/adma.202211579 36637436

[414] L. Huang , R. Xu , S. Chen , C. Lin , W. Li , S. Li , P. E. Saw , L. Zhang , X. Xu , Mol. Cancer 2025, 24, 73. 40059153 10.1186/s12943-025-02274-1 PMC11892139

[415] X. Zhuge , R. Tang , Y. Jiang , L. Lin , D. Xi , H. Yang , Acta Biomater. 2024, 184, 419. 38936754 10.1016/j.actbio.2024.06.029

[416] S. Zhou , L. Zhang , Y. You , K. Yu , X. Tie , Y. Gao , Y. Chen , F. Yao , R. Zhang , X. Hao , C. Fang , X. Li , Q. Li , X. Wang , J. Hepatol. 2025. 10.1016/j.jhep.2025.02.045 40154622

[417] M. Yuan , I. Mahmud , K. Katsushima , K. Joshi , O. Saulnier , R. Pokhrel , B. Lee , W. Liyanage , H. Kunhiraman , S. Stapleton , I. Gonzalez‐Gomez , R. M. Kannan , T. Eisemann , E. Kolanthai , S. Seal , T. J. Garrett , S. Abbasi , K. Bockley , J. Hanes , P. Chapagain , G. Jallo , R. J. Wechsler‐Reya , M. D. Taylor , C. G. Eberhart , A. Ray , R. J. Perera , Acta Neuropathol. Commun. 2023, 11, 203. 38115140 10.1186/s40478-023-01684-w PMC10729563

[418] J. J. Wilson , L. Bennie , O. Eguaogie , A. Elkashif , P. F. Conlon , L. Jena , E. McErlean , N. Buckley , K. Englert , N. J. Dunne , J. H. R. Tucker , J. S. Vyle , H. O. McCarthy , J. Controlled Release 2024, 369, 63. 10.1016/j.jconrel.2024.03.036 38513729

[419] Z. Luo , Y. Huang , S. Chen , B. Zhang , H. Huang , S. Dabiri , Y. Chen , A. Zhang , A. R. Andreas , S. Li , Cancer Lett 2024, 604, 217268. 39321912 10.1016/j.canlet.2024.217268

[420] L. Wang , C. Dai , Y. Fang , X. You , J. Wu , Nano Res. 2022, 15, 4544.

[421] X. Wang , W. Su , Y. Jiang , F. Jia , W. Huang , J. Zhang , Y. Yin , H. Wang , Adv. Sci. 2022, 9, 2200482. 10.1002/advs.202200482 PMC9284143 35508896

[422] P. Yan , Y. Luo , X. Li , Y. Li , Y. Wang , J. Wu , S. Zhou , Adv. Healthcare Mater. 2021, 10, 2101222. 10.1002/adhm.202101222 34494380

[423] Y. Liu , Y. Liu , D. Xu , J. Zang , X. Zheng , Y. Zhao , Y. Li , R. He , S. Ruan , H. Dong , J. Gu , Y. Yang , Q. Cheng , Y. Li , Adv. Sci. 2022, 9, 2104182. 10.1002/advs.202104182 PMC9108638 35306759

[424] Y. Zhao , Z. Xie , Y. Deng , A. Huang , Y. He , B. Wen , X. Liao , R. Chang , G. Zhang , L. Zhu , Y. Wang , T. Li , Y. Zhong , J. Zuo , H. Zhang , M. Chen , J. Liu , X. Chen , H. Liu , Chem. Eng. J. 2022, 450, 138139.

[425] X. Wen , X. Xiong , G. Yang , W. Xiao , J. Hou , T. Pan , Y. Hu , S. Zhou , J. Controlled Release 2023, 353, 535. 10.1016/j.jconrel.2022.12.001 36481693

[426] L. Ren , J. Wan , X. Li , J. Yao , Y. Ma , F. Meng , S. Zheng , W. Han , H. Wang , Nat. Commun. 2024, 15, 7664. 39227567 10.1038/s41467-024-51945-y PMC11372058

[427] Z. Zhang , Q. Zhao , Q. Xu , Q. Deng , A. Hua , X. Wang , X. Yang , Z. Li , Biomaterials 2025, 317, 123094. 39799701 10.1016/j.biomaterials.2025.123094

[428] X. Gao , X. Tang , Z. Tu , J. Yu , Y. Bao , G. Long , W. C. Sheu , H. Wu , J. Liu , J. Zhou , Biomaterials 2025, 317, 123035. 39731842 10.1016/j.biomaterials.2024.123035 PMC11827167

[429] Y. Lv , B. Song , G. Yang , Y. Wang , Z. Wu , M. Si , Z. Yang , H. Chen , C. Liu , M. Li , Y. Zhang , Z. Qiao , L. Wang , W. Xu , Adv. Sci. 2025, 12, 2409425. 10.1002/advs.202409425 PMC11791963 39651805

[430] G. Zhu , Y. Xie , J. Wang , M. Wang , Y. Qian , Q. Sun , Y. Dai , C. Li , Adv. Mater. 2024, 36, 2409066. 10.1002/adma.202409066 39285820

[431] S. Kianamiri , A. Dinari , M. Sadeghizadeh , M. Rezaei , B. Daraei , N. E.‐H. Bahsoun , A. Nomani , Mol. Pharmaceutics 2020, 17, 4483. 10.1021/acs.molpharmaceut.0c00566 33205974

[432] L. Wang , J. Li , Y. Zheng , Y. Li , Q. Zhu , J. Cao , L. Sun , Chem. Eng. J. 2025, 507, 160180.

[433] Q. Zhang , H. Zhuang , X. Wen , Y. Su , J. Wang , H. Qin , J. Wang , Z. Shangguan , Y. Ma , J. Dong , B. Tian , X. Li , Chem. Eng. J. 2025, 506, 159825.

[434] W. Cao , X. Zhang , J. Chen , L. Sun , H. He , F. Yu , Asian J. Pharm. Sci. 2025, 20, 101016. 40224726 10.1016/j.ajps.2025.101016 PMC11987651

[435] L. S. Milane , S. Dolare , G. Ren , M. Amiji , J. Controlled Release 2023, 363, 435. 10.1016/j.jconrel.2023.09.023 37717658

[436] H. Zhang , S. Li , D. Wang , S. Liu , T. Xiao , W. Gu , H. Yang , H. Wang , M. Yang , P. Chen , Biomark. Res. 2024, 12, 96. 39227970 10.1186/s40364-024-00646-1 PMC11373140

[437] Y. Tang , Z. Chen , Q. Zuo , Y. Kang , Cell. Mol. Immunol. 2024, 21, 1215. 39402302 10.1038/s41423-024-01224-z PMC11527989

[438] T. Khan , M. Nagarajan , I. Kang , C. Wu , M. Wangpaichitr , J. Pers. Med. 2025, 15, 50. 39997327 10.3390/jpm15020050 PMC11856717

[439] K. Huang , Y. Han , Y. Chen , H. Shen , S. Zeng , C. Cai , Mol. Cancer 2025, 24, 7. 39789606 10.1186/s12943-024-02205-6 PMC11716519

